# Supplementary material for: Associations between community health workers’ home visits and education-based inequalities in institutional delivery and perinatal mortality in rural Uttar Pradesh, India: a cross-sectional study
Source: BMJ Open. 2021 Jul 12;11(7):e044835. doi: 10.1136/bmjopen-2020-044835 (PMC8276308; doi:10.1136/bmjopen-2020-044835)
Supplement: Supplementary data [file bmjopen-2020-044835supp002.pdf]

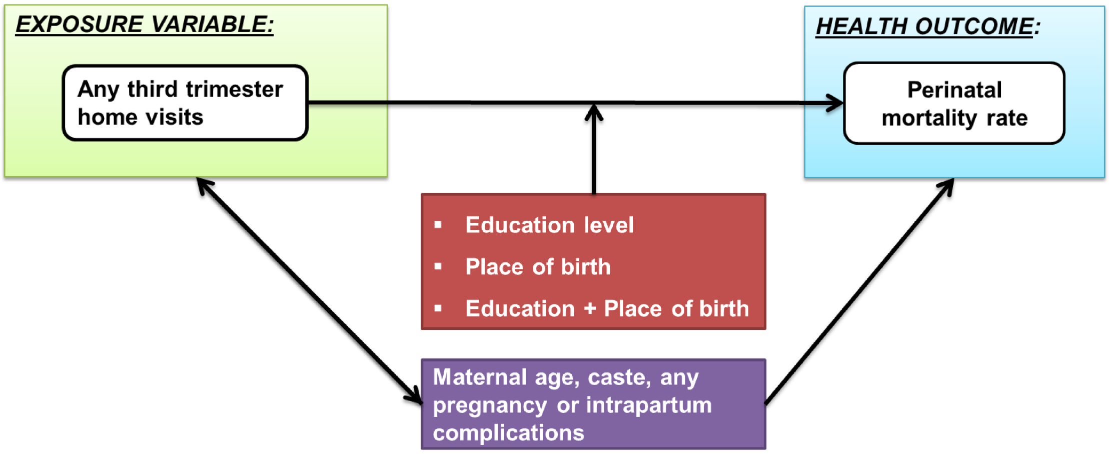

Supplementary figure 2: Conceptual model of the associations between women's exposure to third trimester home visits and perinatal mortality
