# Supplementary figures and images for: Associations between community health workers’ home visits and education-based inequalities in institutional delivery and perinatal mortality in rural Uttar Pradesh, India: a cross-sectional study
Source: BMJ Open. 2021 Jul 12;11(7):e044835. doi: 10.1136/bmjopen-2020-044835 (PMC8276308; doi:10.1136/bmjopen-2020-044835)

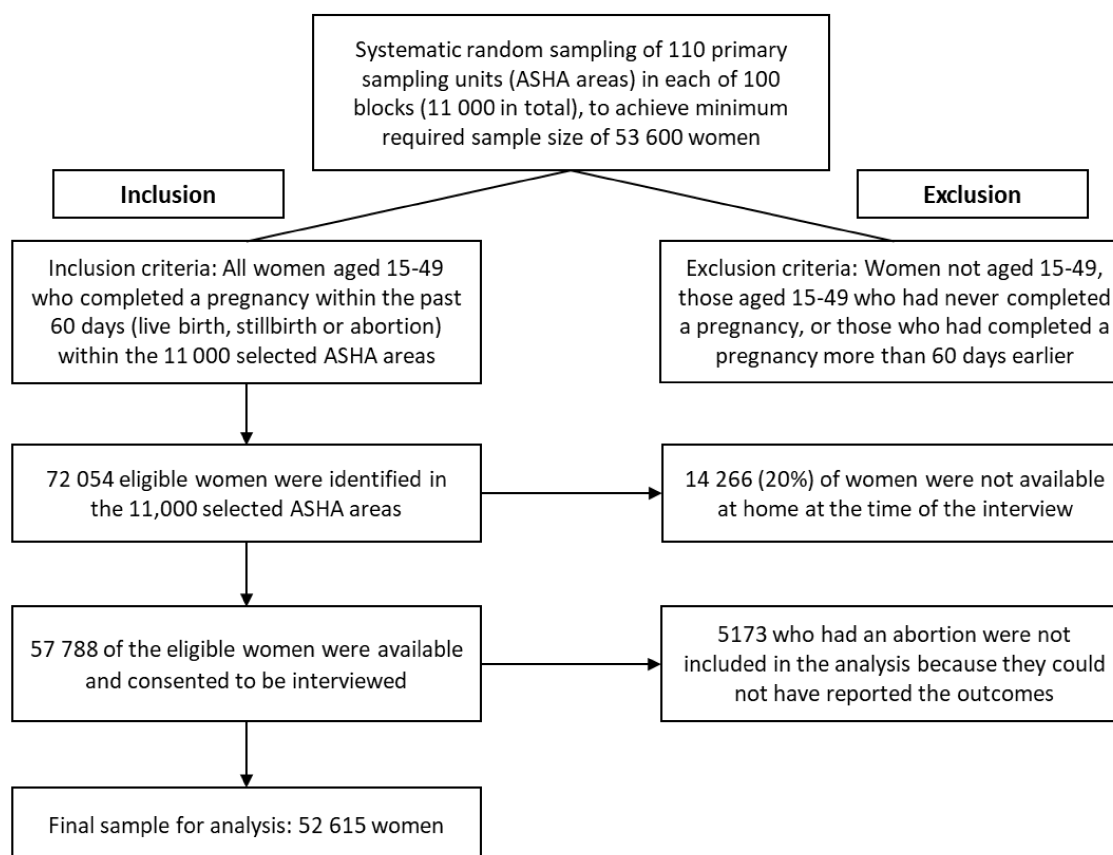

**Supplementary figure 3: Flow diagram of the CBTS-1 sampling process**

Supplement: Supplementary data [file bmjopen-2020-044835supp003.pdf]
