# Supplementary material for: Associations between community health workers’ home visits and education-based inequalities in institutional delivery and perinatal mortality in rural Uttar Pradesh, India: a cross-sectional study
Source: BMJ Open. 2021 Jul 12;11(7):e044835. doi: 10.1136/bmjopen-2020-044835 (PMC8276308; doi:10.1136/bmjopen-2020-044835)
Supplement: Supplementary data [file bmjopen-2020-044835supp005.pdf]

Supplementary table 1: Unadjusted associations between institutional delivery and any third trimester home visits, overall and by education level

Third trimester home visits	All education groups (n=52615)		None / illiterate (n= 30614)		1-5 standards (n=5693)		6-10 standards (n=9895)		>10 standards (n=6413)	
	Unadjusted % (95% CI)	Unadjusted RR (95% CI)	Unadjusted % (95% CI)	Unadjusted RR (95% CI)	Unadjusted % (95% CI)	Unadjusted RR (95% CI)	Unadjusted % (95% CI)	Unadjusted RR (95% CI)	Unadjusted % (95% CI)	Unadjusted RR (95% CI)
Institution (public and private) vs. home										
None (n=23914)	54.2 (53.6, 54.9)	Ref	45.6 (44.8, 46.4)	Ref	56.6 (54.6, 58.5)	Ref	66.0 (64.6, 67.5)	Ref	81.0 (79.5, 82.5)	Ref
Any (n=28701)	71.1 (70.6, 71.6)	1.31 (1.29, 1.33)	64.8 (64.0, 65.5)	1.42 (1.39, 1.45)	72.2 (70.6, 73.7)	1.28 (1.23, 1.33)	78.2 (77.1, 79.3)	1.18 (1.15, 1.22)	86.0 (84.9, 87.1)	1.06 (1.04, 1.09)
Public facility vs. home										
None (n=20997)	47.9 (47.2, 48.6)	Ref	41.0 (40.2, 41.8)	Ref	50.5 (48.3, 52.6)	Ref	59.3 (57.7, 61.0)	Ref	73.2 (71.2, 75.2)	Ref
Any (n=25682)	67.7 (67.1, 68.3)	1.41 (1.39, 1.44)	61.8 (61.0, 62.6)	1.51 (1.47, 1.54)	69.4 (67.7, 71.0)	1.37 (1.31, 1.44)	75.1 (73.9, 76.3)	1.27 (1.23, 1.31)	82.5 (81.1, 83.9)	1.13 (1.09, 1.16)
Private facility vs. home										
None (n=13860)	21.0 (20.4, 21.7)	Ref	12.7 (12.0, 13.4)	Ref	22.0 (19.8, 24.3)	Ref	32.6 (30.6, 34.7)	Ref	60.6 (57.9, 63.2)	Ref
Any (n=11318)	26.7 (25.9, 27.5)	1.27 (1.21, 1.32)	18.3 (17.4, 19.2)	1.44 (1.34, 1.55)	24.8 (22.3, 27.2)	1.12 (0.98, 1.29)	36.4 (34.3, 38.5)	1.11 (1.02, 1.21)	58.8 (56.1, 61.6)	0.97 (0.91, 1.04)
