# Supplementary material for: Associations between community health workers’ home visits and education-based inequalities in institutional delivery and perinatal mortality in rural Uttar Pradesh, India: a cross-sectional study
Source: BMJ Open. 2021 Jul 12;11(7):e044835. doi: 10.1136/bmjopen-2020-044835 (PMC8276308; doi:10.1136/bmjopen-2020-044835)
Supplement: Supplementary data [file bmjopen-2020-044835supp006.pdf]

Supplementary table 2: Unadjusted associations between perinatal mortality rate and any third trimester home visits, overall and by education and place of birth

Third trimester home visits	All education groups (n=52588)		None / illiterate (n= 30600)		1-5 standards (n=5689)		6-10 standards (n=9887)		>10 standards (n=6412)	
	Unadjusted PMR (95% CI)	Unadjusted RR (95% CI)	Unadjusted PMR (95% CI)	Unadjusted RR (95% CI)	Unadjusted PMR (95% CI)	Unadjusted RR (95% CI)	Unadjusted PMR (95% CI)	Unadjusted RR (95% CI)	Unadjusted PMR (95% CI)	Unadjusted RR (95% CI)
All births										
Any (n=28686)	40.8 (38.5, 43.1)	Ref	45.4 (42.2, 48.7)	Ref	37.3 (30.8, 43.8)	Ref	35.1 (30.4, 39.9)	Ref	32.7 (27.0, 38.4)	Ref
None (n=23902)	49.5 (46.8, 52.3)	1.21 (1.12, 1.31)	51.3 (47.7, 54.8)	1.13 (1.02, 1.26)	57.1 (47.9, 66.4)	1.53 (1.21, 1.94)	47.1 (40.7, 53.6)	1.34 (1.11, 1.63)	37.0 (29.8, 44.1)	1.13 (87.3, 1.47)
Home delivery										
Any (n=8295)	36.5 (32.5, 40.6)	Ref	39.9 (34.8, 45.1)	Ref	23.1 (13.3, 32.8)	Ref	34.3 (24.2, 44.4)	Ref	28.9 (14.4, 43.0)	Ref
None (n=10935)	40.7 (37.0, 44.4)	1.11 (0.96, 1.29)	42.4 (37.9, 46.8)	1.06 (0.90, 1.25)	36.2 (24.9, 47.5)	1.57 (0.93, 2.65)	39.1 (29.0, 49.3)	1.14 (0.77, 1.69)	27.6 (13.3, 41.8)	0.96 (0.47, 1.97)
Public facility delivery										
Any (n=17426)	38.0 (35.1, 40.8)	Ref	43.5 (39.3, 47.7)	Ref	35.4 (27.4, 43.4)	Ref	31.5 (25.9, 37.1)	Ref	30.0 (23.4, 36.8)	Ref
None (n=10052)	51.3 (47.0, 55.6)	1.35 (1.21, 1.51)	55.2 (49.2, 61.3)	1.27 (1.10, 1.47)	63.6 (48.9, 78.2)	1.80 (1.30, 2.48)	44.8 (35.9, 53.8)	1.42 (1.09, 1.86)	36.0 (26.2, 45.8)	1.20 (0.84, 1.71)
Private facility delivery										
Any (n=3017)	68.6 (59.6, 77.6)	Ref	84.5 (69.1, 99.9)	Ref	93.3 (60.4, 126.3)	Ref	55.9 (39.0, 72.7)	Ref	44.2 (29.4, 58.9)	Ref
None (n=2915)	76.5 (66.9, 86.1)	1.11 (0.92, 1.34)	93.3 (76.5, 110.0)	1.10 (0.85, 1.42)	107.7 (72.5, 143.0)	1.15 (0.71, 1.87)	70.5 (51.3, 89.8)	1.26 (0.84, 1.90)	44.9 (30.3, 59.4)	1.02 (0.64, 1.62)
