# Supplementary material for: Associations between community health workers’ home visits and education-based inequalities in institutional delivery and perinatal mortality in rural Uttar Pradesh, India: a cross-sectional study
Source: BMJ Open. 2021 Jul 12;11(7):e044835. doi: 10.1136/bmjopen-2020-044835 (PMC8276308; doi:10.1136/bmjopen-2020-044835)
Supplement: Author's manuscript [file bmjopen-2020-044835.draft_revisions.pdf]

BMJ Open

BMJ Open is committed to open peer review. As part of this commitment we make the peer review history of every article we publish publicly available.

When an article is published we post the peer reviewers' comments and the authors' responses online. We also post the versions of the paper that were used during peer review. These are the versions that the peer review comments apply to.

The versions of the paper that follow are the versions that were submitted during the peer review process. They are not the versions of record or the final published versions. They should not be cited or distributed as the published version of this manuscript.

BMJ Open is an open access journal and the full, final, typeset and author-corrected version of record of the manuscript is available on our site with no access controls, subscription charges or pay-per-view fees (http://bmjopen.bmj.com).

If you have any questions on BMJ Open's open peer review process please email info.bmjopen@bmj.com

BMJ Open

The effects of community health workers' home visits on education-based inequalities in perinatal health in rural Uttar Pradesh, India

Journal:	*BMJ Open*
Manuscript ID	bmjopen-2020-044835
Article Type:	Original research
Date Submitted by the Author:	15-Sep-2020

Complete List of Authors:	Blanchard, Andrea; University of Manitoba Faculty of Health Sciences, Community Health Sciences<br>Colbourn, Timothy; UCL Institute for Global Health,<br>Prost, Audrey; University College London Institute of Child Health,<br>Ramesh, Banadakoppa Manjappa; University of Manitoba Faculty of Health Sciences, Department of Community Health Sciences;<br>Isac, Shajy; India Health Action Trust<br>Anthony, John; University of Manitoba, Department of Community Health Sciences; India Health Action Trust<br>Dehuri, Bidyadhar; India Health Action Trust<br>Houweling, TA; Erasmus Medical Center, Department of Public Health

Keywords:	EPIDEMIOLOGY, International health services < HEALTH SERVICES ADMINISTRATION & MANAGEMENT, PUBLIC HEALTH, Maternal medicine < OBSTETRICS, PERINATOLOGY

SCHOLARONE™
Manuscripts

I, the Submitting Author has the right to grant and does grant on behalf of all authors of the Work (as defined in the below author licence), an exclusive licence and/or a non-exclusive licence for contributions from authors who are: i) UK Crown employees; ii) where BMJ has agreed a CC-BY licence shall apply, and/or iii) in accordance with the terms applicable for US Federal Government officers or employees acting as part of their official duties; on a worldwide, perpetual, irrevocable, royalty-free basis to BMJ Publishing Group Ltd ("BMJ") its licensees and where the relevant Journal is co-owned by BMJ to the co-owners of the Journal, to publish the Work in this journal and any other BMJ products and to exploit all rights, as set out in our licence.

The Submitting Author accepts and understands that any supply made under these terms is made by BMJ to the Submitting Author unless you are acting as an employee on behalf of your employer or a postgraduate student of an affiliated institution which is paying any applicable article publishing charge ("APC") for Open Access articles. Where the Submitting Author wishes to make the Work available on an Open Access basis (and intends to pay the relevant APC), the terms of reuse of such Open Access shall be governed by a Creative Commons licence – details of these licences and which Creative Commons licence will apply to this Work are set out in our licence referred to above.

Other than as permitted in any relevant BMJ Author's Self Archiving Policies, I confirm this Work has not been accepted for publication elsewhere, is not being considered for publication elsewhere and does not duplicate material already published. I confirm all authors consent to publication of this Work and authorise the granting of this licence.

For peer review only

The effects of community health workers' home visits on education-based inequalities in perinatal health in rural Uttar Pradesh, India

Andrea K. Blanchard[1], Timothy Colbourn[2], Audrey Prost[2], B.M. Ramesh[1], Shajy Isac[1,3], John Anthony[1,3], Bidyadhar Dehury[3], Tanja A.J. Houweling[4]

1. Institute for Global Public Health, University of Manitoba, Winnipeg, Canada
2. Institute for Global Health, University College London, London, United Kingdom
3. India Health Action Trust, New Delhi & Lucknow, India
4. Department of Public Health, Erasmus MC, University Medical Center Rotterdam, The Netherlands

Correspondence to: Andrea Katryn Blanchard, Andrea.Blanchard@umanitoba.ca

For peer review only

Word count: 4142

Number of references: 54

For peer review only - http://bmjopen.bmj.com/site/about/guidelines.xhtml

Abstract

Objectives: India's National Health Mission has trained community health workers called Accredited Social Health Activists (ASHAs) to visit and counsel women before and after birth. Little is known about the extent to which exposure to ASHAs' home visits has reduced perinatal health inequalities. This study aimed to examine the extent to which ASHAs' third trimester home visits may have contributed to equitable improvements in institutional deliveries and reductions in perinatal mortality rates (PMR) between women with lower to higher education levels in 25 districts of Uttar Pradesh state, India.

Methods: We examined education-based inequalities in institutional delivery and PMR using a cross-sectional survey of a representative sample of 52,615 women who had given birth in the two months prior to interview in rural areas of 25 districts of Uttar Pradesh in 2014/15. We conducted generalised linear modelling.

Results: Third trimester home visits were associated with higher institutional delivery rates, in particular public facility delivery rates (RR 1.32, 95% CI 1.30-1.34), and to a lesser extent private facility delivery rates (RR 1.09, 95% CI 1.04-1.13), after adjusting for confounders. Associations were stronger among women with lower education levels. Having none compared to any third trimester home visits was associated with higher PMR (RR 1.18, 95% CI: 1.09-1.28). Having any versus no visits was more greatly associated with lower PMR among women with lower education levels than those with the most education, and most notably among public facility births.

Conclusions: The study's findings indicated that ASHA home visits in the third trimester contribute to equitable improvements in institutional deliveries and lower PMR, particularly within the public sector. This reinforces the need to support and integrate ASHAs' home visits as a valuable part of India's efforts to achieve the Sustainable Development Goals of improving maternal and newborn health and leaving no one behind.

Abstract word count: 300

Article summary

Strengths and limitations

- This study analysed a large population-based survey that allowed the detection of differences in both institutional delivery rates (and at public and private institutions separately), as well as perinatal

For peer review only

mortality rates at each location, in relation to women's exposure to community health workers' third trimester home visits overall and by education level.

- The cross-sectional survey design precluded conclusions about causality, and the results were not directly generalisable to districts in UP or states in India not included in the survey.
- While important to analyse the evidently large education-based gradients in the outcomes, the survey did not include wealth or asset indices because it was created primarily for programme monitoring.
- The survey responses were self-reported, which could lead to recall, social desirability or misclassification biases.
- The survey included women who gave birth within the last 60 days, which likely improved their recall and classification of perinatal compared to late neonatal deaths.

Background

Since the establishment of the Sustainable Development Goals (SDGs), global health leaders have renewed their pledge to improve maternal and newborn health, with an emphasis on reducing high mortality rates among families who are most socio-economically disadvantaged (1-3). In 2015, 2.7 million infants died in the first month of life, accounting for 45% of child deaths globally, while a further 2.6 million were stillborn (4). In South Asia, over one million neonatal deaths and one million stillbirths are estimated to occur every year, despite considerable progress in the past decades (5). A high proportion of these deaths in South Asia occur in India (6, 7). The greatest number of deaths occur during the perinatal period, i.e. between 28 completed weeks of gestation and seven days after birth (8, 9). Uttar Pradesh (UP) is India's most populous state and has had the highest estimated PMR (10). The uptake of health services for reproductive, maternal, newborn and child health (RMNCH) in many regions of UP have historically been low and inequitable between socio-economic groups (11, 12).

Community-level interventions are a promising approach to equitably improve maternal and newborn health, with most relying on Community Health Workers (CHWs) (13-15). CHWs have been defined as people who live and work in their communities, are selected by and accountable to them, and may or may not be part of the health system (16-18). Recently, the expanded pursuit of primary health care and universal health coverage, including for RMNCH, have given CHW programmes renewed importance in public health programmes (3, 19, 20). Global health leaders have endorsed their potential to improve accessibility to health facilities for the poorest communities where resources are most limited (15, 21, 22). There is also evidence that CHWs can promote safe newborn care practices and prevent PMR (23-27).

For peer review only

For peer review only - http://bmjopen.bmj.com/site/about/guidelines.xhtml

India's National Health Mission (NHM) has recruited and trained a large cadre of CHWs, called Accredited Social Health Activists (ASHAs), to improve RMNCH. ASHAs contact and counsel women and their families on antenatal, delivery, and postnatal care through home visits. ASHAs and the women they counsel normally receive monetary incentives for giving birth at a health facility, and these services are meant to be free at public health facilities. ASHAs' training modules provide guidance on how to reach geographically and socio-economically marginalised families (28). Despite the scale of the ASHA home visiting programme, few studies have examined whether it has equitably improved neonatal health outcomes, and none have examined its effects on perinatal mortality overall or comparing between public and private facility births (29-31). The present study aimed to examine the extent to which ASHAs' home visits in the third trimester of pregnancy may have contributed to equitable improvements in institutional delivery rates at public and private facilities, and reductions in perinatal mortality rates (PMR) between women with lower to higher education levels in 25 districts of Uttar Pradesh state, India.

Methods

Study design and setting

The Government of UP's branch of the NHM has committed substantial resources to reducing the burden of maternal and neonatal mortality. In 2012, UP's NHM programme established a partnership with the India Health Action Trust and the University of Manitoba, Canada, under the auspices of the Uttar Pradesh Technical Support Unit (UP TSU) funded by the Bill and Melinda Gates Foundation. This partnership is embedded within UP's NHM programmes in its 25 high priority districts (HPDs), covering a population of over 83 million people (32). The government chose the HPDs by ranking districts using a composite health index combining six health indicators from the state's Annual Health Survey data (33). This study used data from the first round of UP TSU's cross-sectional Community Behavioural Tracking Survey (CBTS-1) collected in April 2014 to February 2015, which included 100 blocks (sub-districts) of the 25 HPDs.

Sampling and participants

The CBTS-1 primary sampling unit (PSU) was an ASHA area, which covers approximately 1000 people, and is the smallest unit of health service delivery. A required sample size of 536 per block was calculated to detect a minimum percentage change of 7.5% in the institutional delivery rate within six months (a key indicator expected to change between survey rounds), assuming an estimated non-response rate of 15%. This was considered an ideal sample size given the resources for data collection compared to what was required for minimum detectable changes of 5% (n=1209) and 10% (n=300). The average number of PSUs

For peer review only - http://bmjopen.bmj.com/site/about/guidelines.xhtml

needed to meet this sample size was estimated to be 110 based on the crude birth rate of UP in the most recent available data (29 per 1000 population in the Annual Health Survey in 2011/12). All PSUs in the 100 blocks of 25 districts were listed, and 110 were chosen through systematic random sampling. Every eligible woman in each PSU was enumerated and invited to participate, and PSUs were of nearly uniform size. Eligible participants were women aged 15 to 49 who had delivered in the past sixty days. 72,054 women were identified and 57,788 were included, leading to an 80% response rate. There were no refusals to participate; non-response occurred largely when women were repeatedly unavailable at home when the interviewers visited on different days. We analysed data from 52,615 participants, excluding 5,173 women who responded that their pregnancy ended in an abortion.

Data collection

Field interviewers received ten days of training on data collection methods. The interviewers first determined the PSU boundary. With a random start, they visited all households and gave each household a number. They used a screening questionnaire in each household to identify women who recently completed a pregnancy, and then described the study and asked for their informed consent. The survey tool included close-ended questions related to selected background characteristics, pregnancy outcome, antenatal care, birth preparedness, delivery, postnatal and newborn care, and reproductive health. The interviewers collected the data anonymously using mobile applications, which was quality checked routinely by field supervisors.

Variables

Independent variables

Equity in health has been defined as the absence of systematic disparities in health or its social determinants between groups with differing positions in a social hierarchy (34). A number of socio-economic position characteristics are used to describe inequities in health, in terms of resource-based dimensions like wealth or asset indices, education levels, or occupation, as well as identity-based dimensions such as caste, ethnicity or religion (34, 35). Wealth or asset-based inequalities would have been valuable to assess, but were not available in the first round of the CBTS with the largest sample. We examined caste inequalities but there were more consistent caste differences in institutional delivery rates than PMR, which were inequitable among private but not public deliveries. Thus, we focused on education as the main indicator of socio-economic position in this study as there were consistent inequalities in both outcomes. Higher maternal education has been associated with child survival and

For peer review only - http://bmjopen.bmj.com/site/about/guidelines.xhtml

utilisation of maternal health services in previous research (36). Education levels represent socio-economic opportunities that are achieved early in life and remain fairly stable over the lifetime, relatively uninfluenced by later health status. It is also an ordinal variable and enables the examination of gradients in health outcomes (37). We categorised education levels into four groups from least to most years of study (standards) completed based on common Indian school system divisions: none/illiterate, 1 to 5 (lower primary school), 6 to 10 (higher primary to lower secondary), or more than 10 standards (upper secondary and college) (37).

Receipt of ASHA home visits in the third trimester of pregnancy was the main intervention exposure variable. The third trimester is when ASHAs are meant to focus on counselling women about birth preparedness (identification of transportation and desired place of birth), having an institutional delivery, and essential newborn care practices associated with reducing perinatal mortality (38). We considered exposure to home visits earlier in pregnancy, but found that only 9.8% of women who had any home visits did not have one during the third trimester.

Outcome variables

The outcomes of interest were institutional delivery and perinatal mortality rates. Due to differing patterns in women's exposure to third trimester home visits and education at each delivery place, we compared outcomes of institutional delivery (public and private) versus home, as well as separately conducting analyses for births at a public facility versus home and a private facility versus home. The PMR was defined as the number of stillbirths (occurring between 28 weeks of gestation and delivery) and deaths during the early neonatal period (first seven days of life) out of 1000 births. These were self-reported and not confirmed by verbal autopsies.

Covariate and confounding variables

Covariates that were initially thought to be related to the exposure and/or outcome variables were included if they were found to be significant at $p<0.05$ in bivariable models. Maternal age, caste and religion were adjusted for as confounders in all multivariable models. We also included any antenatal check-ups and any pregnancy complications for both outcomes, as well as intrapartum complications and then place of delivery for perinatal mortality, as potential mediator variables. Conceptual models are shown in Supplementary figures 1 and 2, indicating the hypothesised relationships between exposure and outcome variables, and the coviariates included in each analysis.

For peer review only - http://bmjopen.bmj.com/site/about/guidelines.xhtml

Statistical analyses

Analyses were conducted in STATA 13.0. We first used cross-tabulations to describe the exposure variable, outcomes and covariates (shown in Table 1). We produced figures to visualise education group differences in the proportion of births at home, public and private facilities, and PMR at each of these locations, by exposure to third trimester home visits (Figures 1 and 2). Then we used bivariable and multivariable generalised linear models (GLM) to assess whether the outcomes were independently associated with having any third trimester home visits. Lastly, we compared the associations between the institutional delivery and PMR outcomes and women's exposure to third trimester home visits by education group and then place of birth using stratified analyses. Interaction terms between exposure to home visits and the outcomes were initially also examined. These were significant for institutional delivery in the adjusted models, but not for perinatal mortality rates; the latter showed uneven gradients by education groups that were likely obscured in the interaction term. Thus, we focused on the stratified results as they were more interpretable and transparent about where uneven patterns of inequalities and associations existed.

In the multivariable models with institutional delivery as the outcome (shown in the Table 2), first the model was adjusted for maternal age, caste, religion, any pregnancy complications and any antenatal check-ups. Second, the model was adjusted for the same variables but stratified by education. Thirdly, we ran adjusted models for institutional delivery separately for births at public facilities versus home, in relation to women's exposure to home visits overall and then comparing each education group.

In the multivariable models with PMR as the outcome (results in Table 3), all models were adjusted for maternal age, caste, any pregnancy complications and any intrapartum complications. Religion and any antenatal check-ups were not significantly associated with PMR in the bivariable models and therefore not included. First, we produced the model without place of birth and then adjusted for place of birth. We also stratified the model by education and then by place of birth separately, and finally by education and place of birth at once, to assess how the relationships between PMR and third trimester home visits may be different.

In the GLM, we used both absolute (rate difference, RD) and relative (rate ratio, RR) effect measures. These were calculated using a binomial distribution, with an identity link function and a log link function respectively (39). Some adjusted GLM for institutional delivery as outcome failed to converge. Others have found that this can occur with variables having few data points in some categories, and have addressed this by using a Poisson distribution with robust standard errors to produce the RR (40). Our robust Poisson

For peer review only - http://bmjopen.bmj.com/site/about/guidelines.xhtml

models with a log link function produced estimates that were almost identical to our analyses using a log binomial distribution in models where convergence did not fail. For institutional delivery as outcome, the RD and RR (using robust Poisson distribution) are presented. For PMR, we also obtained the adjusted average rates to present alongside the adjusted RR from the GLM models (using the log binomial distribution, as there was no failed convergence).

All data collection protocols and analysis plans were approved by the Institutional Review Board of Sigma Research and Consulting in New Delhi, and the University College London Research Ethics Committee.

Patient and public involvement statement

No patients or members of the public were involved in this study.

Results

Over 80% of women were 15 to 29 years old (Table 1). The majority of women belonged to Other Backward Class groups, followed by Scheduled Caste and General Caste groups. Nearly 60% of women were illiterate or had no formal education. Over half of women (55%) received any home visits during the third trimester, and nearly 50% had gone for any ANC check-up. Less than 20% of women reported having any pregnancy complications, while almost 40% reported intrapartum complications.

Table 1: Descriptive socio-demographic and health-related characteristics of the sample

Variable	Total, n (%)	Any third trimester home visit (%)	Institutional delivery			Perinatal mortality rate (per 1000 births)
			Total (%)	Public facility (%)	Private facility (%)	
**Overall**	52615 (100)	55	63	52	11	45
**Age**						
15-24 years	21079 (40)	55	70	56	14	49
25-29 years	21400 (41)	55	62	51	11	39
30+ years	10136 (19)	53	55	47	8	49
**Religion**						
Hindu	42720 (81)	55	64	53	11	45
Muslim/Other	9895 (19)	51	59	46	13	42
**Caste**						
Scheduled Tribe	2216 (4)	40	55	49	6	43
Scheduled Caste	12334 (23)	55	62	54	8	44
Other Backward Class	24579 (47)	55	63	52	11	45

For peer review only - http://bmjopen.bmj.com/site/about/guidelines.xhtml

Variable	Total, n (%)	Any third trimester home visit (%)	Institutional delivery			Perinatal mortality rate (per 1000 births)
			Total (%)	Public facility (%)	Private facility (%)	
General Caste	6323 (12)	53	72	53	19	48
Don't know	7163 (14)	57	61	49	12	44
**Education**						
None/ illiterate	30614 (58)	52	56	48	8	48
1-5 standards	5693 (11)	57	66	55	11	46
6-10 standards	9895 (19)	58	73	59	14	40
>10 standards	6413 (12)	58	84	60	24	35
**Any pregnancy complications[a.]**						
No	43102 (82)	55	63	52	11	38
Yes	9513 (18)	51	67	53	14	76
**Any intrapartum complications[b.]**						
No	32000 (61)	55	60	51	9	32
Yes	20615 (39)	53	69	54	15	65
**Any ANC check-ups**						
No	27179 (52)	44	54	45	9	45
Yes	25436 (48)	66	73	59	14	45
**Any third trimester home visits**						
No	23914 (45)	NA	54	42	12	50
Yes	28701 (55)	NA	71	61	10	41

[a.] Pregnancy complications were defined as experiencing any of the following: excessive bleeding before delivery, convulsions, high blood pressure, or sepsis/fever.
[b.] Intrapartum complications were defined as experiencing any of the following: premature labour, preterm/premature rupture of the membrane, prolonged labour (>12 hours), obstructed labour, breach/malpresentation, or excessive bleeding immediately after delivery.

For peer review only

Institutional delivery

Almost two-thirds (63%) of women gave birth at a health facility (52% in a public facility, 11% in a private facility). Institutional delivery rates were higher for women with more education, ranging from 56% among women who were illiterate or uneducated to 84% among those with more than 10 standards. These educational inequalities were apparent for both public and private facilities.

For peer review only - http://bmjopen.bmj.com/site/about/guidelines.xhtml

A greater proportion of women having any versus no third trimester home visits reported giving birth in a public facility (61% vs. 42% respectively). While lower overall, an equal proportion had private facility births without or with any third trimester home visits (12% vs. 10% respectively).

Women who had received any third trimester home visit compared to none had a higher institutional delivery rate overall (71% vs. 54%) (RD 16.8 [95%CI: 16.0, 17.7], RR 1.31 [95%CI: 1.29, 1.33]). This association remained significant after adjusting for confounders (aRD 13.0 [95%CI: 12.8-14.5], aRR 1.23 [95%CI: 1.22, 1.25]) (Table 2). The adjusted associations between having an institutional birth and any third trimester home visit was stronger with each step down the educational gradient.

[Figure 1]

Figure 1 illustrates that there was a noticeably higher proportion of women giving birth at public facilities compared to home when they had any compared to no third trimester visits from an ASHA, and particularly among the least educated groups. In Table 2, the adjusted association between having any third trimester home visit and an institutional birth was stronger for births at a public facility (aRR 1.32 [95% CI: 1.30-1.34]) than those at a private facility (aRR 1.08 [95% CI: 1.04-1.13]) compared to home. This was observed for all educational groups, but particularly so for less educated women.

For peer review only - http://bmjopen.bmj.com/site/about/guidelines.xhtml

For peer review only

Table 2: Associations between place of birth and any third trimester home visits, overall and by education level

Exposure to third trimester home visits	All education groups		None / illiterate		1-5 standards		6-10 standards		>10 standards	
	RD (95% CI)	RR (95% CI)	RD (95% CI)	RR (95% CI)	RD (95% CI)	RR (95% CI)	RD (95% CI)	RR (95% CI)	RD (95% CI)	RR (95% CI)
Institution (public or private) vs. home										
Any vs. none, unadjusted	16.8 (16.0, 17.7)	1.31 (1.29, 1.33)	19.2 (18.1, 20.2)	1.42 (1.39, 1.45)	15.6 (13.1, 18.1)	1.28 (1.23, 1.33)	12.2 (10.4, 14.0)	1.18 (1.15, 1.22)	5.0 (3.1, 6.8)	1.06 (1.04, 1.09)
Any vs. none, adjusted*	13.0 (12.8, 14.5)[a.]	1.23 (1.22, 1.25)	15.6 (14.4, 16.7)	1.33 (1.30, 1.36)	13.5 (10.9, 16.0)	1.24 (1.19, 1.29)	9.9 (8.1, 11.7)	1.15 (1.12, 1.18)	3.5 (1.7, 5.4)	1.05 (1.03, 1.07)
Public facility vs. home										
Any vs. none, unadjusted	19.8 (18.9, 20.7)	1.41 (1.39, 1.44)	20.8 (19.7, 21.9)	1.51 (1.47, 1.54)	18.9 (16.2, 21.6)	1.37 (1.31, 1.44)	15.8 (13.7, 17.8)	1.27 (1.23, 1.31)	9.3 (6.8, 11.7)	1.13 (1.09, 1.16)
Any vs. none, adjusted*	15.4 (14.5, 16.3)[a.]	1.32 (1.30, 1.34)	17.4 (16.3, 18.6)	1.41 (1.38, 1.45)	16.7 (13.9, 19.5)	1.33 (1.26, 1.39)	13.4 (11.3, 15.4)	1.22 (1.18, 1.26)	7.7 (5.2, 10.1)	1.11 (1.07, 1.14)
Private facility vs. home										
Any vs. none, unadjusted	5.6 (4.6, 6.7)	1.27 (1.21, 1.32)	5.6 (4.5, 6.7)	1.44 (1.34, 1.55)	2.7 (-0.6, 6.0)	1.12 (0.98, 1.29)	3.7 (0.8, 6.7)	1.11 (1.02, 1.21)	-1.7 (-5.5, 2.1)	0.97 (0.91, 1.04)
Any vs. none, adjusted*	2.4 (1.7, 3.1)[a.]	1.09 (1.04, 1.13)	2.5 (1.4, 3.6)	1.21 (1.12, 1.31)	1.2 (-2.0, 4.4)	1.05 (0.91, 1.20)	1.2 (-1.6, 4.0)	1.04 (0.95, 1.13)	-3.5 (-7.2, 0.2)	0.94 (88.7, 1.00)

*Models adjusted for maternal age, caste, religion, any pregnancy complications and ANC check-ups.

[a.] Convergence failure occurred with this identity link model, therefore estimates taken after 50 iterations. All RR models run using a robust Poisson distribution and log link for consistency.

For peer review only - http://bmjopen.bmj.com/site/about/guidelines.xhtml

Perinatal mortality rate

The overall perinatal mortality rate was 45 per 1000 births (Table 1). The greatest number of early neonatal deaths occurred on the day of birth, and steadily decreased in number on subsequent days (supplementary Figure 3). Perinatal mortality reduced with increasing educational attainment, from 48 to 35 per 1000 births among women with no education compared to more than ten standards respectively. The PMR was 39 per 1000 births among women giving birth at home, 43 per 1000 births at public facilities, and 73 per 1000 births at private facilities overall.

The PMR was higher among women without compared to with any third trimester home visit, at 50 compared to 41 per 1000 births respectively (RR 1.21 [95% CI: 1.12, 1.31]) (supplementary Table 1). The association remained after adjustment for socio-demographic characteristics, any pregnancy or intrapartum complications, and any ANC visits (aRR 1.17 [95% CI: 1.98, 1.27]), and after additional adjustment for place of birth (aRR 1.18 [95% CI: 1.09, 1.28]) (Table 3).

The association between having no third trimester home visits and higher PMR was stronger for those with one to five standards of education (aRR 1.53 [95% CI: 1.20, 1.94]), and to a lesser extent 5 to 10 standards (aRR 1.27 [95% CI: 1.05, 1.54]). The association was weaker for women with no education (aRR 1.13 [95% CI: 1.02, 1.25]), and absent among women who had an education over ten standards.

The inverse association between any third trimester home visits and PMR was stronger for births at public facilities (aRR 1.31 [95% CI: 1.17, 1.47]). There was no association for births at private facilities (aRR 1.12 [95% CI: 0.93, 1.34]) nor for home births (aRR 1.04 [95% CI: 0.91, 1.21]).

[Figure 2]

Figure 2 shows that for women who had any versus no third trimester home visits, educational differences in PMR appeared to be smaller among public facility births and to a lesser extent private facility births. In adjusted analyses, PMR was significantly higher among those without any home visits compared to those with any home visits for public facility births, except in the most educated group (Table 3). However, differences in PMR between those with and without third trimester home visits were not significant among women who delivered at home or in private facilities, across all education groups. Confidence intervals were wide in some of the comparisons based on lower numbers of perinatal deaths.

For peer review only - http://bmjopen.bmj.com/site/about/guidelines.xhtml

Table 3: Adjusted associations between perinatal mortality rate and any third trimester home visits, overall and by education and place of birth

Third trimester home visits	All education groups		None/ illiterate		1-5 standards		6-10 standards		>10 standards	
	PMR (95% CI)	RR (95% CI)	PMR (95% CI)	RR (95% CI)	PMR (95% CI)	RR (95% CI)	PMR (95% CI)	RR (95% CI)	PMR (95% CI)	RR (95% CI)
All births, not adjusting for place of birth										
Any	41.5 (39.2, 43.8)	Ref	45.9 (42.7, 49.2)	Ref	37.8 (21.2, 44.3)	Ref	35.6, 30.8, 40.4)	Ref	33.1 (27.3, 38.9)	Ref
None	48.6 (45.9, 51.3)	1.17 (1.08, 1.27)	50.7 (47.2, 54.2)	1.10 (1.00, 1.22)	56.1 (47.0, 65.1)	1.48 (1.17, 1.88)	46.3 (40.0, 52.6)	1.30 (1.07, 1.58)	36.3 (29.3, 43.3)	1.10 (0.84, 1.42)
All births, adjusting for place of birth										
Any	41.3 (39.0, 43.6)	Ref	45.5 (42.2, 48.7)	Ref	37.3 (30.8, 43.8)	Ref	36.0 (31.1, 40.9)	Ref	33.3 (27.5, 39.2)	Ref
None	48.9 (46.1, 51.6)	1.18 (1.09, 1.28)	51.2 (47.7, 54.8)	1.13 (1.02, 1.25)	56.9 (47.7, 66.2)	1.53 (1.20, 1.94)	45.7 (39.4, 52.0)	1.27 (1.05, 1.54)	35.9 (28.9, 42.9)	1.07 (0.83, 1.40)
Home delivery										
Any	37.8 (33.7, 42.0)	Ref	41.1 (35.8, 46.4)	Ref	23.7 (13.7, 33.7)	Ref	35.5 (25.1, 45.9)	Ref	30.0 (14.9, 45.1)	Ref
None	39.7 (36.1, 43.3)	1.04 (0.91, 1.21)	41.6 (37.2, 45.9)	1.01 (0.86, 1.19)	35.4 (24.4, 46.5)	1.50 (0.88, 2.53)	38.0 (28.2, 47.8)	1.07 (0.72, 1.58)	26.3 (12.6, 40.0)	0.88 (0.42, 1.82)
Public facility delivery										
Any	38.4 (35.5, 41.3)	Ref	43.8 (39.6, 48.0)	Ref	35.6 (27.6, 43.6)	Ref	31.8 (26.2, 37.4)	Ref	30.4 (23.6, 37.2)	Ref
None	50.4 (46.2, 54.6)	1.31 (1.17, 1.47)	54.5 (48.5, 60.4)	1.24 (1.08, 1.44)	62.7 (48.3, 43.6)	1.76 (1.28, 2.43)	44.0 (35.3 (52.8)	1.38 (1.06, 1.81)	35.3 (25.7, 44.9)	1.16 (0.82, 1.65)
Private facility delivery										
Any	68.5 (59.6, 77.4)	Ref	84.8 (69.6, 100.0)	Ref	96.0 (62.4, 129.6)	Ref	56.9 (40.0, 73.8)	Ref	43.5 (29.0, 58.0)	Ref
None	76.5 (67.0, 86.1)	1.12 (0.93, 1.34)	93.1 (76.6, 109.5)	1.10 (0.85, 1.41)	104.5 (70.8, 138.8)	1.09 (0.68, 1.76)	69.4 (50.7, 88.0)	1.22 (0.82, 1.82)	45.5 (30.8, 60.2)	1.05 (0.66, 1.66)

For peer review only - http://bmjopen.bmj.com/site/about/guidelines.xhtml

Discussion

This study sought to examine the effects of women's exposure to ASHAs' third trimester home visits on education-based inequalities in institutional delivery and perinatal mortality rates in 25 districts of UP, India. The results of this study indicate that having any ASHA home visits during women's third trimester of pregnancy was associated with giving birth in public facilities, and this association was increasingly strong with decreasing education level. Perinatal mortality rates were found to be highest for those delivering at private facilities, and among the least educated women in particular. Women's receipt of any ASHA home visit in the third trimester was significantly associated with lower PMR, and most strongly for women giving birth in public facilities among all but the most educated.

There were some limitations to the analyses. The CBTS-1 survey responses were self-reported, and therefore prone to recall, misclassification and social desirability biases. The survey included women who delivered in the past 60 days to improve recall. The measure of an 'early' neonatal death could have been over- or under-reported, though combining stillbirths and early neonatal deaths to measure PMR can avoid the common misclassification of early neonatal deaths as stillbirths. We did not observe heaping of reported neonatal deaths within the first six days after birth, and the proportion of neonatal deaths at various times after delivery was similar across socio-economic groups. The CBTS-1 offered a sufficiently large population-based sample to measure PMR, and to stratify the analyses by education and place of delivery. However, its purpose as an efficient programme monitoring methodology resulted in lack of data on some relevant factors or confounders, such as women's wealth, parity, newborn's sex, or having a singleton or twin birth that might have attenuated the strength of observed associations if included (41). The cross-sectional nature of the analysis precluded conclusions about causality. Moreover, we assumed that the associations between exposure and outcome variables could involve reverse or multidirectional, rather than linear, pathways of causation (42). For example, ASHAs may have more often chosen to visit women who preferred public facility births as a way to receive their incentives, while women with lower education may more often opt for the less expensive public services. Also, the results may not be directly generalisable to non-HPD districts of UP, where institutional delivery rates were likely higher and PMR lower and possibly less unequal, or to other states in which ASHAs' activities or the levels and types of facilities providing RMNCH services may differ.

Our results suggested that having any third trimester home visits from an ASHA was associated with a greater chance of women having an institutional delivery, and particularly at public facilities. Further,

For peer review only - http://bmjopen.bmj.com/site/about/guidelines.xhtml

having any third trimester home visits was more greatly associated with having an institutional delivery for mothers with lower education levels. Other studies have reported positive associations between exposure to community-based interventions and lower socio-economic inequalities in institutional delivery and skilled birth attendance in UP. A cross-sectional analysis by Seth et al. found that having any ASHA home visit was associated with higher rates of institutional delivery among poorer and illiterate women but not among richer or more educated women (30). Baqui et al.'s quasi-experimental study of an intervention involving NGO technical support to the ASHA programme in two districts of UP found that skilled birth attendance became more equitable between wealth groups in the intervention district but remained inequitable in the comparison district (29). The role of ASHAs' home visits in increasing the proportion of institutional deliveries, and particularly at public facilities, among women with less education and to a lesser extent more education may be due to their role in promoting the free public health services and incentives within the National Health Mission. Studies have reported that ASHAs have generally played the strongest role during pregnancy to encourage women to attend public antenatal and delivery care, in part by promoting the *Janani Suraksha Yojana* (JSY) incentives, and that this has occurred alongside widespread shifts in social norms in favour of facility-based maternity care in India (43-46). Our findings seem to support evidence indicating that ASHAs' home visits in the third trimester have played a role in improving institutional delivery rates not only overall but equitably by facilitating access for less educated women to the more affordable care in the public health system in UP.

This study indicated that ASHAs' home visits played the strongest role in supporting women of lower education groups to give birth at public facilities. Yet women from the lower, and even higher, education groups who delivered at private facilities still had high rates of perinatal mortality, though there were fewer cases leading to wider confidence intervals. Researchers have discussed how improvements in institutional delivery rates have not always translated into reductions in mortality among mothers and newborns where there has been lower quality of services and poor referral mechanisms, particularly at primary health care facilities or unregulated private services where people of lower socio-economic positions often attend first (47, 48). More research is needed to understand the processes by which inequitably high perinatal mortality rates continue for women of lower socio-economic positions, especially those who face pregnancy and intrapartum complications, such as the barriers they face in reaching timely higher-level public and especially private health services.

In 2018, the Government of India launched the *Ayushman Bharat* Programme (ABP) to enhance the availability of free integrated primary health care services through village-level Health and Wellness

For peer review only - http://bmjopen.bmj.com/site/about/guidelines.xhtml

Centers (HWCs) and an insurance scheme called *Pradhan Mantri Jan Arogya Yojana* for use of secondary- and tertiary-level care services that targets lower socio-economic groups (49). The programme aims to attach five ASHAs to each HWC, where they will work with new mid-level health providers. ASHAs are to continue home visits among other activities to promote utilisation and safe health behaviours for RMNCH among many other health services (49). Among their range of responsibilities, it will be crucial to support ASHAs not only in promoting and facilitating institutional births but in counselling on and ensuring timely identification of high-risk pregnancies and neonatal complications, especially for women that experience greater socio-economic and geographical disadvantages (3, 7, 49, 50). Building on the NHM, the ABP policies are also explicitly based on the premise that the health system functions effectively (49). The ABP includes public as well as empanelled private first referral units to be covered by the insurance scheme, which should remove financial barriers for women with birth complications that need them (51, 52). As more women and their families in rural areas are opting to give birth at health facilities, it is particularly important to consider the pluralistic health system in India holistically when addressing MNH inequities (48, 51, 53, 54). This would necessitate targeting differential gaps in accessibility, quality and integration of both public and private health services so as to strategically address persistent inequalities in perinatal mortality.

Conclusions

This study findings suggested that ASHAs' contacts and counselling for pregnant women through home visits in the third trimester may have influenced them to give birth at a public facility, particularly among those with lower compared to higher education levels. Further, less educated women who had any compared to no home visits and delivered at a public facility appeared to have significantly lower rates of perinatal deaths. The remaining socio-economic inequalities in PMR among institutional deliveries, which were particularly high in the private sector, may have resulted from barriers to accessing timely and quality services when women required emergency obstetric care not offered at the lower-level facilities where they first sought care. In future, research, policies and programmes should consider the pathways by which community health workers may be reducing socio-economic inequalities in perinatal health outcomes, and how broader contextual and health system changes can strategically support these efforts.

For peer review only - http://bmjopen.bmj.com/site/about/guidelines.xhtml

Funding

This work was supported by the Bill & Melinda Gates Foundation [OPP1161429]. AKB's time was funded by University College London's Overseas and Graduate Research Scholarships. TAJH's time was funded by a Research Excellence Initiative grant from Erasmus University Rotterdam, The Netherlands. The funders had no role in the design of the study, data collection and analysis, planning or writing of the manuscript.

Contributor statement

AKB planned and conducted the analyses, and wrote the draft manuscript. TAJH, TC and AP provided guidance on the design of the study and support throughout the analyses. BMR, SI, JA, and BD designed the CBTS methodology, managed the data collection and quality assurance, and gave technical input on the analyses. All authors read and gave input on the manuscript.

Competing interests

The authors declare no competing interests.

Data sharing statement

The data are hosted on the University of Manitoba server, and a de-identified data cut along with the questionnaires and data dictionary may be made available upon reasonable request (contact e-mail: Ramesh.BanadakoppaManjappa@umanitoba.ca).

Bibliography

1. Houweling TAJ, Morrison J, Azad K, Manandhar DS, Alcock G, Shende S, et al. How to reach every newborn: Three key messages. The Lancet Global Health. 2014;2(8):e436-e7. Available from: doi:10.1016/S2214-109X(14)70271-2.

2. Requejo JH, Bryce J, Barros AJ, Berman P, Bhutta Z, Chopra M, et al. Countdown to 2015 and beyond: fulfilling the health agenda for women and children. Lancet. 2015;385(9966):466-76. Available from: doi:10.1016/S0140-6736(14)60925-9.

3. Boerma T, Requejo J, Victora CG, Amouzou A, George A, Agyepong I, et al. Countdown to 2030: tracking progress towards universal coverage for reproductive, maternal, newborn, and child health. The Lancet. 2018;391(10129):1538-48. Available from: doi:10.1016/S0140-6736(18)30104-1.

4. You D, Hug, L, Ejdemyr, S, Beise, J. Levels and Trends in Child Mortality Report 2015. Geneva: Inter-Agency Group for Child Mortality Estimation, 2015.

For peer review only - http://bmjopen.bmj.com/site/about/guidelines.xhtml

5. Akseer N, Kamali M, Arifeen SE, Malik A, Bhatti Z, Thacker N, et al. Progress in maternal and child health: how has South Asia fared? Bmj. 2017;357:j1608. Available from: doi:10.1136/bmj.j1608.

6. WHO/ UNICEF/ UNFPA/ World Bank Group/ United Nations Population Division. Trends in maternal mortality: 1990 to 2015. Geneva: 2015.

7. Black RE, Levin C, Walker N, Chou D, Liu L, Temmerman M. Reproductive, maternal, newborn, and child health: key messages from Disease Control Priorities 3rd Edition. The Lancet. 2016;388(10061):2811-24. Available from: doi:http://dx.doi.org/10.1016/S0140-6736(16)00738-8.

8. United Nations Children Fund. Neonatal Mortality. Geneva: UNICEF; 2018 [cited 2018 March 12]. Available from: https://data.unicef.org/topic/child-survival/neonatal-mortality/

9. World Health Organization. Maternal and perinatal health. Geneva: World Health Organization; 2018 [cited 2018 March 12]. Available from: http://www.who.int/maternal_child_adolescent/topics/maternal/maternal_perinatal/en/.

10. International Institute for Population Sciences (IIPS) and ICF. National Family Health Survey (NFHS-4), 2015-16: India. Mumbai: Ministry of Health and Family Welfare, 2017.

11. Dettrick Z, Jimenez-Soto E, Hodge A. Socioeconomic and geographical disparities in under-five and neonatal mortality in Uttar Pradesh, India. Maternal and Child Health Journal. 2014;18(4):960-9. Available from: doi:10.1007/s10995-013-1324-8.

12. International Institute for Population Sciences (IIPS) and ICF. National Family Health Survey (NFHS-4), India, 2015-16: Uttar Pradesh. Mumbai: 2017.

13. Bhutta ZA, Das JK, Rizvi A, Gaffey MF, Walker N, Horton S, et al. Evidence-based interventions for improvement of maternal and child nutrition: what can be done and at what cost? Lancet. 2013;382(9890):452-77. Available from: doi:10.1016/s0140-6736(13)60996-4.

14. Ekman B, Pathmanathan I, Liljestrand J. Integrating health interventions for women, newborn babies, and children: a framework for action. Lancet. 2008;372(9642):990-1000. Available from: doi:10.1016/S0140-6736(08)61408-7.

15. Bhutta ZA, Black RE. Global maternal, newborn, and child health - So near and yet so far. New England Journal of Medicine. 2013;369(23):2226-35. Available from: doi:10.1056/NEJMra1111853.

16. Olaniran A, Smith H, Unkels R, Bar-Zeev S, van den Broek N. Who is a community health worker? – a systematic review of definitions. Glob Health Action. 2017;10(1):1-13. Available from: doi:10.1080/16549716.2017.1272223.

17. World Health Organization. Community health workers: a strategy to ensure access to primary health care services. Cairo: Regional Office for the Eastern Mediterranean, 2016.

18. Tulenko K, Mogedal S, Afzal MM, Frymus D, Oshin A, Pate M, et al. Community health workers for universal health-care coverage: from fragmentation to synergy. Bull World Health Organ. 2013;91(11):847-52. Available from: doi:10.2471/BLT.13.118745.

For peer review only - http://bmjopen.bmj.com/site/about/guidelines.xhtml

19. Perry H. A Brief History of Community Health Worker Programs. In: Perry H, Crigler L. (eds.) Developing and Strengthening Community Health Worker Programs at Scale - A Reference Guide and Case Studies for Program Managers and Policymakers. Washington: United States Agency for International Development; 2014. p. 1-14.

20. The Lancet. The Astana Declaration: the future of primary health care? The Lancet. 2018;392(10156):1369. Available from: doi:10.1016/S0140-6736(18)32478-4.

21. Victora CG, Wagstaff A, Schellenberg JA, Gwatkin D, Claeson M, Habicht J-P. Applying an equity lens to child health and mortality: more of the same is not enough. The Lancet. 2003;362(9379):233-41. Available from: doi:https://doi.org/10.1016/S0140-6736(03)13917-7.

22. Bhutta ZA, Reddy KS. Achieving equity in global health: So near and yet so far. JAMA - Journal of the American Medical Association. 2012;307(19):2035-6. Available from: doi:10.1001/jama.2012.4659.

23. Gilmore B, McAuliffe E. Effectiveness of community health workers delivering preventive interventions for maternal and child health in low- and middle-income countries: a systematic review. BMC public health. 2013;13(847):1-14.

24. Gogia S, Sachdev HS. Home visits by community health workers to prevent neonatal deaths in developing countries: a systematic review. Bulletin of the World Health Organization. 2010;88(9):658-66B. Available from: doi:http://dx.doi.org/10.2471/BLT.09.069369.

25. Prost A, Pagel C, Costello A. Community interventions to reduce maternal and child mortality in lowincome countries. Maternal and Infant Deaths: Chasing Millennium Development Goals 4 and 52010. p. 205-16.

26. Haines A, Sanders D, Lehmann U, Rowe AK, Lawn JE, Jan S, et al. Achieving child survival goals: potential contribution of community health workers. Lancet. 2007;369(9579):2121-31. Available from: doi:10.1016/s0140-6736(07)60325-0.

27. Lassi ZS, Bhutta ZA. Community-based intervention packages for reducing maternal and neonatal morbidity and mortality and improving neonatal outcomes. Cochrane Database Syst Rev. 2015(3):1-78. Available from: doi:10.1002/14651858.CD007754.pub3.

28. Ministry of Health and Family Welfare. ASHA Training Modules - National Health Mission. Delhi: Government of India; 2014 [cited 2017 March 28]. Available from: http://nrhm.gov.in/communitisation/asha/resources/asha-training-modules.html.

29. Baqui AH, Rosecrans AM, Williams EK, Agrawal PK, Ahmed S, Darmstadt GL, et al. NGO facilitation of a government community-based maternal and neonatal health programme in rural India: improvements in equity. Health Policy Plan. 2008;23(4):234-43. Available from: doi:http://dx.doi.org/10.1093/heapol/czn012.

30. Seth A, Tomar S, Singh K, Chandurkar D, Chakraverty A, Dey A, et al. Differential effects of community health worker visits across social and economic groups in Uttar Pradesh, India: a link between social inequities and health disparities. International Journal for Equity in Health. 2017;16(46):1-9. Available from: doi:10.1186/s12939-017-0538-6.

For peer review only

31. Rajbhandari S, Hodgins S, Sanghvi H, McPherson R, Pradhan YV, Baqui AH, et al. Expanding uterotonic protection following childbirth through community-based distribution of misoprostol: operations research study in Nepal. Int J Gynaecol Obstet. 2010;108(3):282-8. Available from: doi:http://dx.doi.org/10.1016/j.ijgo.2009.11.006.

32. Bill and Melinda Gates Foundation. Maternal, Newborn and Child Health. Seattle: Bill and Melinda Gates Foundation; 2014 [cited 2017 December 12]. Available from: http://www.gatesfoundation.org/What-We-Do/Global-Development/Maternal-Newborn-and-Child-Health.

33. Ministry of Health and Family Welfare. Guidance note for implementation of RMNCH+A interventions in High Priority Districts. New Delhi: Government of India, 2013.

34. Braveman P, Gruskin S. Defining equity in health. J Epidemiol Commun Health. 2003;57:254-8. Available from: doi:10.1136/jech.57.4.254.

35. Marmot M. Closing the health gap in a generation: the work of the Commission on Social Determinants of Health and its recommendations. Glob Health Promot. 2009;Suppl 1:23-7. Available from: doi:10.1177/1757975909103742.

36. Gabrysch S, Campbell OM. Still too far to walk: literature review of the determinants of delivery service use. BMC Pregnancy Childbirth. 2009;9(34). Available from: doi:10.1186/1471-2393-9-34.

37. Solar O, Irwin A. A conceptual framework for action on the social determinants of health. Geneva: World Health Organization, 2010.

38. Ministry of Health and Family Welfare. Guidelines for Community Processes - National Rural Health Mission. New Delhi: Government of India, 2014.

39. Khang Y-H, Lynch JW, Yang S, Harper S, Yun S-C, Jung-Choi K, et al. The contribution of material, psychosocial, and behavioral factors in explaining educational and occupational mortality inequalities in a nationally representative sample of South Koreans: Relative and absolute perspectives. Social Science & Medicine. 2009;68(5):858-66. Available from: doi:http://dx.doi.org/10.1016/j.socscimed.2008.12.003.

40. Zou G. A Modified Poisson Regression Approach to Prospective Studies with Binary Data. American Journal of Epidemiology. 2004;159(7):702-6. Available from: doi:10.1093/aje/kwh090.

41. Barros AJD, Victora CG. Measuring Coverage in MNCH: Determining and Interpreting Inequalities in Coverage of Maternal, Newborn, and Child Health Interventions. PLoS Med. 2013;10(5):e1001390. Available from: doi:10.1371/journal.pmed.1001390.

42. VanderWeele TJ. Mediation Analysis: A Practitioner's Guide. Annu Rev Public Health. 2016;37:17-32. Available from: doi:10.1146/annurev-publhealth-032315-021402.

43. Scott K, George AS, Ved RR. Taking stock of 10 years of published research on the ASHA programme: examining India's national community health worker programme from a health systems perspective. Health Research Policy and Systems. 2019;17(1):29. Available from: doi:10.1186/s12961-019-0427-0.

For peer review only

44. Sidney K, Tolhurst R, Jehan K, Diwan V, De Costa A. 'The money is important but all women anyway go to hospital for childbirth nowadays' - a qualitative exploration of why women participate in a conditional cash transfer program to promote institutional deliveries in Madhya Pradesh, India. BMC Pregnancy and Childbirth. 2016;16(1):47. Available from: doi:10.1186/s12884-016-0834-y.

45. Bruce SG, Blanchard AK, Gurav K, Roy A, Jayanna K, Mohan HL, et al. Preferences for infant delivery site among pregnant women and new mothers in Northern Karnataka, India. BMC Pregnancy Childbirth. 2015;15(49):1-10. Available from: doi:10.1186/s12884-015-0481-8.

46. Lim SS, Dandona L, Hoisington JA, James SL, Hogan MC, Gakidou E. India's Janani Suraksha Yojana, a conditional cash transfer programme to increase births in health facilities: an impact evaluation. Lancet. 2009;375(9730):2009-23. Available from: doi:10.1016/s0140-6736(10)60744-1.

47. Sharma J, Leslie HH, Regan M, Nambiar D, Kruk ME. Can India's primary care facilities deliver? A cross-sectional assessment of the Indian public health system's capacity for basic delivery and newborn services. BMJ Open. 2018;8(6):e020532. Available from: doi:10.1136/bmjopen-2017-020532.

48. McPake B, Hanson K. Managing the public & private mix to achieve universal health coverage. The Lancet. 2016;388(10044):622-30. Available from: doi:10.1016/S0140-6736(16)00344-5.

49. Ministry of Health and Family Welfare. Ayushman Bharat: Comprehensive Primary Health Care through Health and Wellness Centers Operational Guidelines. New Delhi: Government of India, 2018.

50. Barros AJ, Ronsmans C, Axelson H, Loaiza E, Bertoldi AD, Franca GV, et al. Equity in maternal, newborn, and child health interventions in Countdown to 2015: a retrospective review of survey data from 54 countries. Lancet. 2012;379(9822):1225-33. Available from: doi:10.1016/S0140-6736(12)60113-5.

51. Angell BJ, Prinja S, Gupt A, Jha V, Jan S. The Ayushman Bharat Pradhan Mantri Jan Arogya Yojana and the path to universal health coverage in India: Overcoming the challenges of stewardship and governance. PLOS Medicine. 2019;16(3):e1002759. Available from: doi:10.1371/journal.pmed.1002759.

52. Department of Rural Development. Socio Economic and Caste Census 2011. New Delhi: Government of India; 2011 [cited 2019 July 3]. Available from: https://secc.gov.in/reportlistContent.

53. Upadhyay RP, Krishnan A, Rai SK, Chinnakali P, Odukoya O. Need to Focus Beyond the Medical Causes: a Systematic Review of the Social Factors Affecting Neonatal Deaths. Paediatr Perinat Epidemiol. 2014;28(2):127-37. Available from: doi:10.1111/ppe.12098.

54. Wall SN, Lee AC, Carlo W, Goldenberg R, Niermeyer S, Darmstadt GL, et al. Reducing intrapartum-related neonatal deaths in low- and middle-income countries-what works? Semin Perinatol. 2010;34(6):395-407. Available from: doi:10.1053/j.semperi.2010.09.009.

For peer review only

For peer review only - http://bmjopen.bmj.com/site/about/guidelines.xhtml

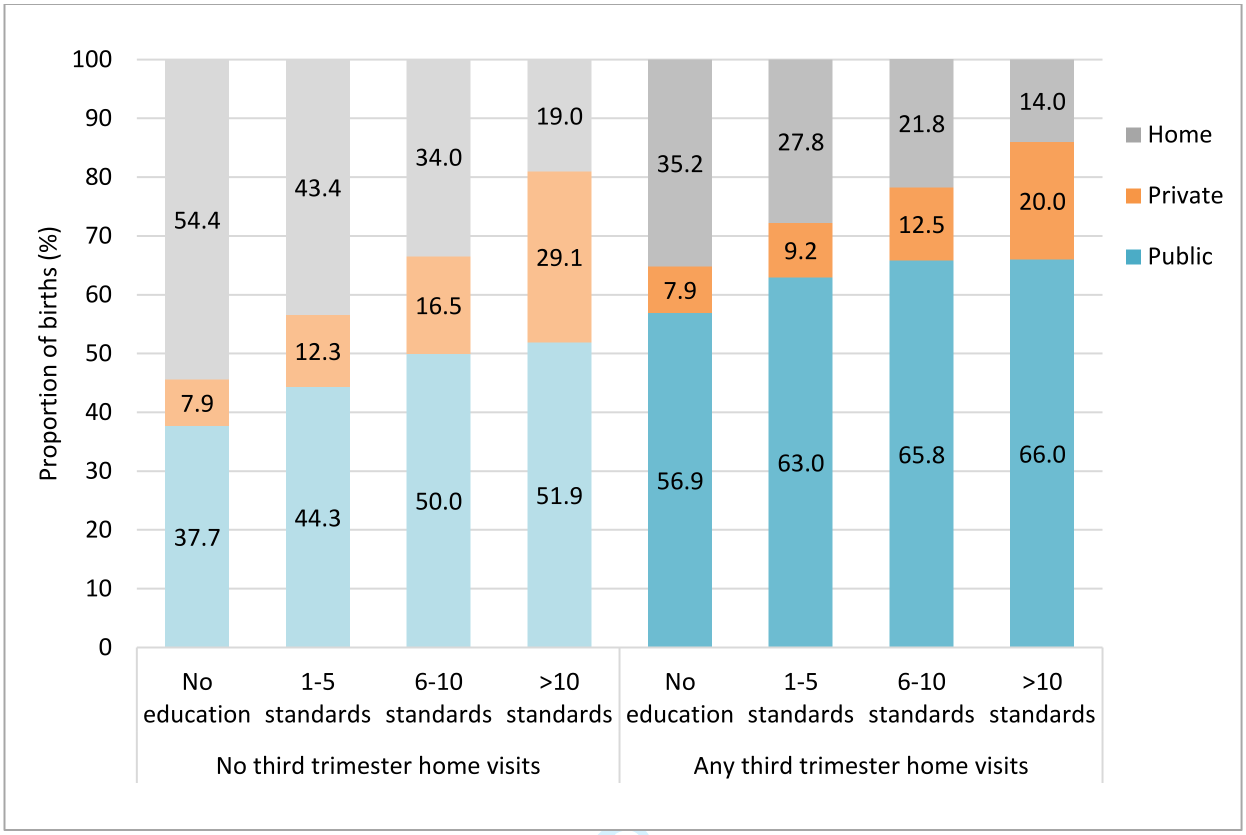

Figure 1: Proportion of women in each education group giving birth at home, public facilities, or private facilities, by exposure to any third trimester home visits

For peer review only

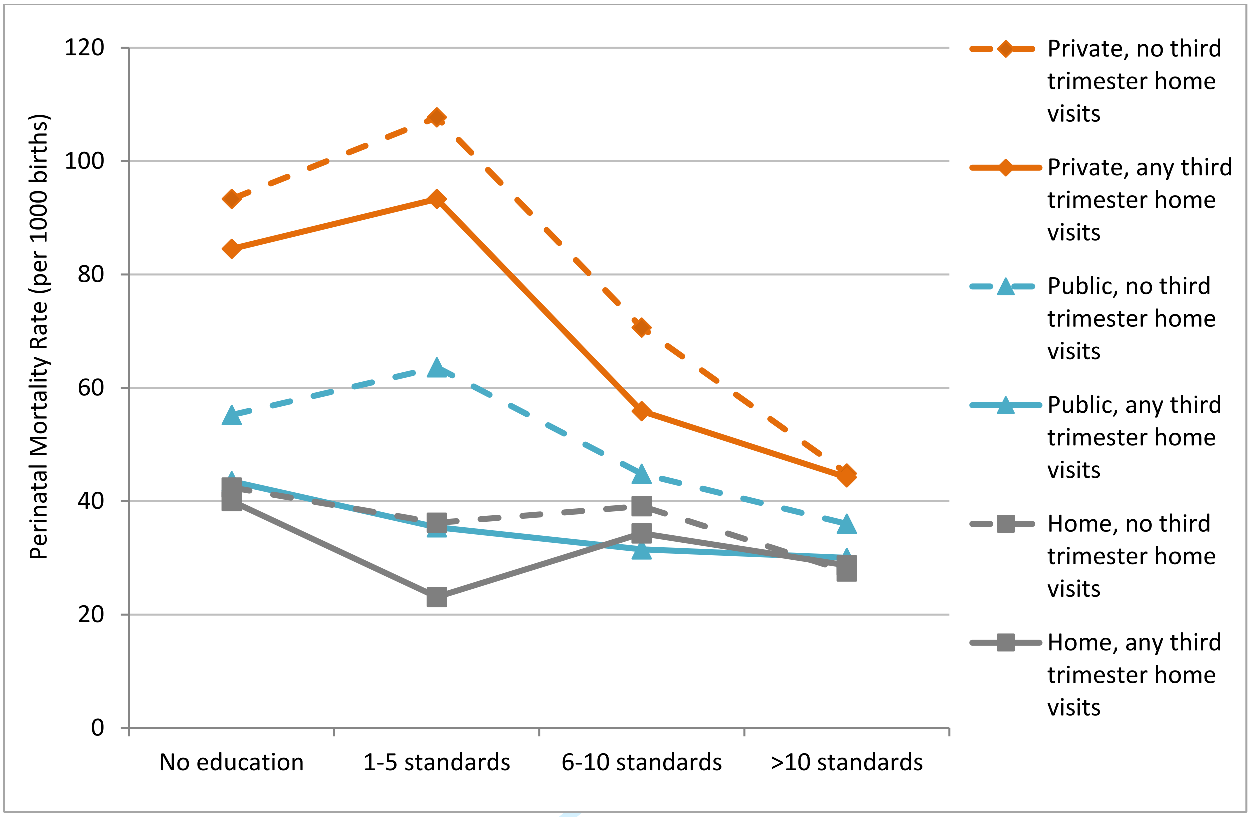

Figure 2: PMR among women in each education group, by place of birth and exposure to any third trimester home visits

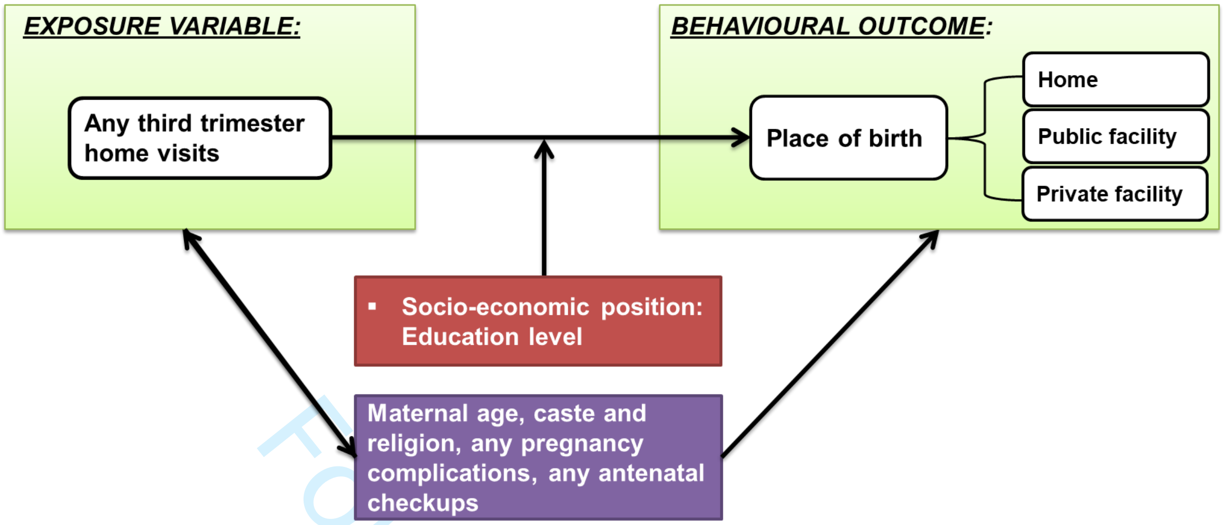

Supplementary figure 1: Conceptual model of the associations between women's exposure to third trimester home visits and place of birth

For peer review only

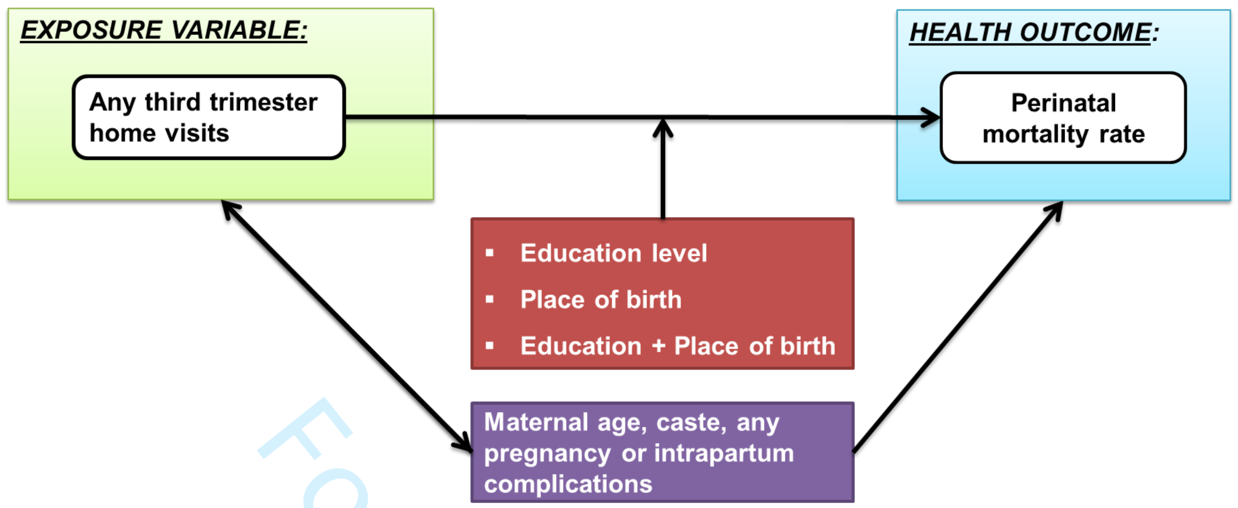

Supplementary figure 2: Conceptual model of the associations between women's exposure to third trimester home visits and perinatal mortality

For peer review only

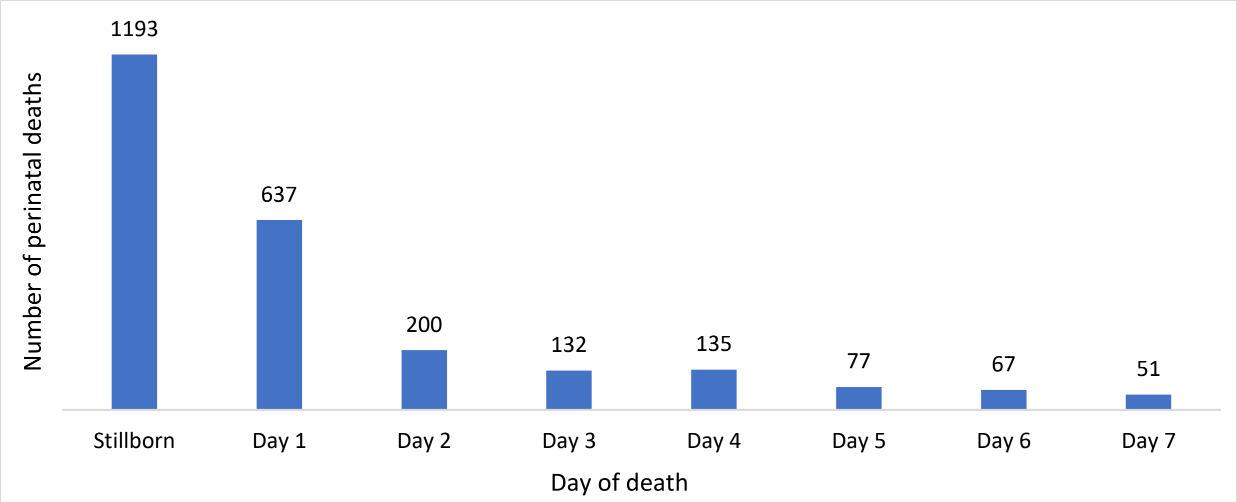

Supplementary figure 3: Distribution and timing of perinatal deaths among women who completed a pregnancy in the past two months

For peer review only

Supplementary table 1: Unadjusted associations between institutional delivery and any third trimester home visits, overall and by education level

Third trimester home visits	All education groups (n=52615)		None / illiterate (n= 30614)		1-5 standards (n=5693)		6-10 standards (n=9895)		>10 standards (n=6413)	
	Unadjusted % (95% CI)	Unadjusted RR (95% CI)	Unadjusted % (95% CI)	Unadjusted RR (95% CI)	Unadjusted % (95% CI)	Unadjusted RR (95% CI)	Unadjusted % (95% CI)	Unadjusted RR (95% CI)	Unadjusted % (95% CI)	Unadjusted RR (95% CI)
Institution (public and private) vs. home										
None (n=23914)	54.2 (53.6, 54.9)	Ref	45.6 (44.8, 46.4)	Ref	56.6 (54.6, 58.5)	Ref	66.0 (64.6, 67.5)	Ref	81.0 (79.5, 82.5)	Ref
Any (n=28701)	71.1 (70.6, 71.6)	1.31 (1.29, 1.33)	64.8 (64.0, 65.5)	1.42 (1.39, 1.45)	72.2 (70.6, 73.7)	1.28 (1.23, 1.33)	78.2 (77.1, 79.3)	1.18 (1.15, 1.22)	86.0 (84.9, 87.1)	1.06 (1.04, 1.09)
Public facility vs. home										
None (n=20997)	47.9 (47.2, 48.6)	Ref	41.0 (40.2, 41.8)	Ref	50.5 (48.3, 52.6)	Ref	59.3 (57.7, 61.0)	Ref	73.2 (71.2, 75.2)	Ref
Any (n=25682)	67.7 (67.1, 68.3)	1.41 (1.39, 1.44)	61.8 (61.0, 62.6)	1.51 (1.47, 1.54)	69.4 (67.7, 71.0)	1.37 (1.31, 1.44)	75.1 (73.9, 76.3)	1.27 (1.23, 1.31)	82.5 (81.1, 83.9)	1.13 (1.09, 1.16)
Private facility vs. home										
None (n=13860)	21.0 (20.4, 21.7)	Ref	12.7 (12.0, 13.4)	Ref	22.0 (19.8, 24.3)	Ref	32.6 (30.6, 34.7)	Ref	60.6 (57.9, 63.2)	Ref
Any (n=11318)	26.7 (25.9, 27.5)	1.27 (1.21, 1.32)	18.3 (17.4, 19.2)	1.44 (1.34, 1.55)	24.8 (22.3, 27.2)	1.12 (0.98, 1.29)	36.4 (34.3, 38.5)	1.11 (1.02, 1.21)	58.8 (56.1, 61.6)	0.97 (0.91, 1.04)

For peer review only

Supplementary table 2: Unadjusted associations between perinatal mortality rate and any third trimester home visits, overall and by education and place of birth

Third trimester home visits	All education groups (n=52615)		None/ illiterate (n=30614)		1-5 standards (n=5693)		6-10 standards (n=9895)		>10 standards (n=6413)	
	Unadjusted PMR (95% CI)	Unadjusted RR (95% CI)	Unadjusted PMR (95% CI)	Unadjusted RR (95% CI)	Unadjusted PMR (95% CI)	Unadjusted RR (95% CI)	Unadjusted PMR (95% CI)	Unadjusted RR (95% CI)	Unadjusted PMR (95% CI)	Unadjusted RR (95% CI)
All births										
Any (n=28686)	40.8 (38.5, 43.1)	Ref	45.4 (42.2, 48.7)	Ref	37.3 (30.8, 43.8)	Ref	35.1 (30.4, 39.9)	Ref	32.7 (27.0, 38.4)	Ref
None (n=23902)	49.5 (46.8, 52.3)	1.21 (1.12, 1.31)	51.3 (47.7, 54.8)	1.13 (1.02, 1.26)	57.1 (47.9, 66.4)	1.53 (1.21, 1.94)	47.1 (40.7, 53.6)	1.34 (1.11, 1.63)	37.0 (29.8, 44.1)	1.13 (87.3, 1.47)
Home delivery										
Any (n=8295)	36.5 (32.5, 40.6)	Ref	39.9 (34.8, 45.1)	Ref	23.1 (13.3, 32.8)	Ref	34.3 (24.2, 44.4)	Ref	28.9 (14.4, 43.0)	Ref
None (n=10935)	40.7 (37.0, 44.4)	1.11 (0.96, 1.29)	42.4 (37.9, 46.8)	1.06 (90.0, 1.25)	36.2 (24.9, 47.5)	1.57 (0.93, 2.65)	39.1 (29.0, 49.3)	1.14 (77.0, 1.69)	27.6 (13.3, 41.8)	0.96 (0.47, 1.97)
Public facility delivery										
Any (n=17426)	38.0 (35.1, 40.8)	Ref	43.5 (39.3, 47.7)	Ref	35.4 (27.4, 43.4)	Ref	31.5 (25.9, 37.1)	Ref	30.0 (23.4, 36.8)	Ref
None (n=10052)	51.3 (47.0, 55.6)	1.35 (1.21, 1.51)	55.2 (49.2, 61.3)	1.27 (1.10, 1.47)	63.6 (48.9, 78.2)	1.80 (1.30, 2.48)	44.8 (35.9, 53.8)	1.42 (1.09, 1.86)	36.0 (26.2, 45.8)	1.20 (0.84, 1.71)
Private facility delivery										
Any (n=3017)	68.6 (59.6, 77.6)	Ref	84.5 (69.1, 99.9)	Ref	93.3 (60.4, 126.3)	Ref	55.9 (39.0, 72.7)	Ref	44.2 (29.4, 58.9)	Ref
None (n=2915)	76.5 (66.9, 86.1)	1.11 (92.9, 1.34)	93.3 (76.5, 110.0)	1.10 (0.85, 1.42)	107.7 (72.5, 143.0)	1.15 (0.71, 1.87)	70.5 (51.3, 89.8)	1.26 (0.84, 1.90)	44.9 (30.3, 59.4)	1.02 (0.64, 1.62)

For peer review only

STROBE 2007 (v4) Statement—Checklist of items that should be included in reports of *cross-sectional studies*

Section/Topic	Item #	Recommendation	Reported on page #
**Title and abstract**	1	(*a*) Indicate the study's design with a commonly used term in the title or the abstract	2
		(*b*) Provide in the abstract an informative and balanced summary of what was done and what was found	2
**Introduction**			
Background/rationale	2	Explain the scientific background and rationale for the investigation being reported	3-4
Objectives	3	State specific objectives, including any prespecified hypotheses	4
**Methods**			
Study design	4	Present key elements of study design early in the paper	4
Setting	5	Describe the setting, locations, and relevant dates, including periods of recruitment, exposure, follow-up, and data collection	4-5
Participants	6	(*a*) Give the eligibility criteria, and the sources and methods of selection of participants	4-5
Variables	7	Clearly define all outcomes, exposures, predictors, potential confounders, and effect modifiers. Give diagnostic criteria, if applicable	5-6
Data sources/ measurement	8*	For each variable of interest, give sources of data and details of methods of assessment (measurement). Describe comparability of assessment methods if there is more than one group	5-6
Bias	9	Describe any efforts to address potential sources of bias	5, 14
Study size	10	Explain how the study size was arrived at	4-5
Quantitative variables	11	Explain how quantitative variables were handled in the analyses. If applicable, describe which groupings were chosen and why	7-8
Statistical methods	12	(*a*) Describe all statistical methods, including those used to control for confounding	7-8
		(*b*) Describe any methods used to examine subgroups and interactions	7
		(*c*) Explain how missing data were addressed	NA
		(*d*) If applicable, describe analytical methods taking account of sampling strategy	NA
		(*e*) Describe any sensitivity analyses	NA
**Results**			

For peer review only

Participants	13*	(a) Report numbers of individuals at each stage of study—eg numbers potentially eligible, examined for eligibility, confirmed eligible, included in the study, completing follow-up, and analysed	5
		(b) Give reasons for non-participation at each stage	5
		(c) Consider use of a flow diagram	NA
Descriptive data	14*	(a) Give characteristics of study participants (eg demographic, clinical, social) and information on exposures and potential confounders	8-9
		(b) Indicate number of participants with missing data for each variable of interest	NA
Outcome data	15*	Report numbers of outcome events or summary measures	8-10, 12
Main results	16	(*a*) Give unadjusted estimates and, if applicable, confounder-adjusted estimates and their precision (eg, 95% confidence interval). Make clear which confounders were adjusted for and why they were included	10-13, Figures 1-2, supplementary Table 1
		(*b*) Report category boundaries when continuous variables were categorized	NA
		(*c*) If relevant, consider translating estimates of relative risk into absolute risk for a meaningful time period	9-11
Other analyses	17	Report other analyses done—eg analyses of subgroups and interactions, and sensitivity analyses	10-13, Figures 1-2
**Discussion**			
Key results	18	Summarise key results with reference to study objectives	14
Limitations	19	Discuss limitations of the study, taking into account sources of potential bias or imprecision. Discuss both direction and magnitude of any potential bias	14
Interpretation	20	Give a cautious overall interpretation of results considering objectives, limitations, multiplicity of analyses, results from similar studies, and other relevant evidence	14-16
Generalisability	21	Discuss the generalisability (external validity) of the study results	14
**Other information**			
Funding	22	Give the source of funding and the role of the funders for the present study and, if applicable, for the original study on which the present article is based	17

*Give information separately for cases and controls in case-control studies and, if applicable, for exposed and unexposed groups in cohort and cross-sectional studies.

Note: An Explanation and Elaboration article discusses each checklist item and gives methodological background and published examples of transparent reporting. The STROBE checklist is best used in conjunction with this article (freely available on the Web sites of PLoS Medicine at http://www.plosmedicine.org/, Annals of Internal Medicine at http://www.annals.org/, and Epidemiology at http://www.epidem.com/). Information on the STROBE Initiative is available at www.strobe-statement.org.

For peer review only

BMJ Open

Associations between community health workers' home visits and lower education-based inequalities in institutional delivery and perinatal mortality in rural Uttar Pradesh, India

Journal:	*BMJ Open*
Manuscript ID	bmjopen-2020-044835.R1
Article Type:	Original research
Date Submitted by the Author:	10-Feb-2021
Complete List of Authors:	Blanchard, Andrea; University of Manitoba Faculty of Health Sciences, Community Health Sciences<br>Colbourn, Timothy; UCL Institute for Global Health,<br>Prost, Audrey; University College London Institute of Child Health,<br>Ramesh, Banadakoppa Manjappa; University of Manitoba Faculty of Health Sciences, Department of Community Health Sciences;<br>Isac, Shajy; India Health Action Trust<br>Anthony, John; University of Manitoba, Department of Community Health Sciences; India Health Action Trust<br>Dehuri, Bidyadhar; India Health Action Trust<br>Houweling, TA; Erasmus Medical Center, Department of Public Health
**Primary Subject Heading**:	Global health
Secondary Subject Heading:	Public health, Epidemiology
Keywords:	EPIDEMIOLOGY, International health services < HEALTH SERVICES ADMINISTRATION & MANAGEMENT, PUBLIC HEALTH, Maternal medicine < OBSTETRICS, PERINATOLOGY

SCHOLARONE™
Manuscripts

I, the Submitting Author has the right to grant and does grant on behalf of all authors of the Work (as defined in the below author licence), an exclusive licence and/or a non-exclusive licence for contributions from authors who are: i) UK Crown employees; ii) where BMJ has agreed a CC-BY licence shall apply, and/or iii) in accordance with the terms applicable for US Federal Government officers or employees acting as part of their official duties; on a worldwide, perpetual, irrevocable, royalty-free basis to BMJ Publishing Group Ltd ("BMJ") its licensees and where the relevant Journal is co-owned by BMJ to the co-owners of the Journal, to publish the Work in this journal and any other BMJ products and to exploit all rights, as set out in our licence.

The Submitting Author accepts and understands that any supply made under these terms is made by BMJ to the Submitting Author unless you are acting as an employee on behalf of your employer or a postgraduate student of an affiliated institution which is paying any applicable article publishing charge ("APC") for Open Access articles. Where the Submitting Author wishes to make the Work available on an Open Access basis (and intends to pay the relevant APC), the terms of reuse of such Open Access shall be governed by a Creative Commons licence – details of these licences and which Creative Commons licence will apply to this Work are set out in our licence referred to above.

Other than as permitted in any relevant BMJ Author's Self Archiving Policies, I confirm this Work has not been accepted for publication elsewhere, is not being considered for publication elsewhere and does not duplicate material already published. I confirm all authors consent to publication of this Work and authorise the granting of this licence.

For peer review only

Associations between community health workers' home visits and lower education-based inequalities in institutional delivery and perinatal mortality in rural Uttar Pradesh, India

Andrea K. Blanchard[1], Timothy Colbourn[2], Audrey Prost[2], B.M. Ramesh[1], Shajy Isac[1,3], John Anthony[1,3], Bidyadhar Dehury[3], Tanja A.J. Houweling[4]

1. Institute for Global Public Health, University of Manitoba, Winnipeg, Canada
2. Institute for Global Health, University College London, London, United Kingdom
3. India Health Action Trust, New Delhi & Lucknow, India
4. Department of Public Health, Erasmus MC, University Medical Center Rotterdam, The Netherlands

Correspondence to: Andrea Katryn Blanchard, Andrea.Blanchard@umanitoba.ca

Word count: 4668

Number of references: 57

For peer review only

Abstract

Introduction: India's National Health Mission has trained community health workers called Accredited Social Health Activists (ASHAs) to visit and counsel women before and after birth. Little is known about the extent to which exposure to ASHAs' home visits has reduced perinatal health inequalities. This study aimed to examine the extent to which ASHAs' third trimester home visits may contribute to equitable improvements in institutional delivery and reductions in perinatal mortality rates between women of varying education levels in Uttar Pradesh state, India.

Methods: Cross-sectional survey data was collected among a representative sample of 52,615 women who gave birth in the preceding two months in rural areas of 25 districts of Uttar Pradesh in 2014/15. We analyzed the data using generalised linear modelling to examine the associations between exposure to home visits and education-based inequalities in institutional delivery and perinatal mortality.

Results: Third trimester home visits were associated with higher institutional delivery rates, in particular public facility delivery rates (adjusted RR 1.32, 95% CI 1.30-1.34), and to a lesser extent private facility delivery rates (aRR 1.09, 95% CI 1.04-1.13), after adjusting for confounders. Associations were stronger among women with lower education levels. Having none compared to any third trimester home visits was associated with higher perinatal mortality (aRR 1.18, 95% CI: 1.09-1.28). Having any versus no visits was more greatly associated with lower perinatal mortality among women with lower education levels than those with the most education, and most notably among public facility births.

Conclusions: The study's findings indicate that ASHAs' home visits in the third trimester contributed to equitable improvements in institutional deliveries and lower perinatal mortality rates, particularly within the public sector. Broader strategies must buttress the role of ASHAs' home visits in improving the Sustainable Development Goals of improving maternal and newborn health and leaving no one behind.

Abstract word count: 300

For peer review only

Article summary

Strengths and limitations

- This study analysed a large population-based survey that allowed the detection of education-based inequalities in institutional delivery and perinatal mortality rates overall and at public and private health facilities separately, and whether these inequalities were lower among women who received community health workers' home visits in the third trimester.
- The cross-sectional survey design precluded conclusions about causality, and the results are not directly generalisable to non-high priority districts in Uttar Pradesh or other states in India.
- While important to analyse the evidently large education-based gradients in the outcomes, the first round of the survey did not include wealth or asset indices because it was created primarily for efficient programme monitoring.
- The survey responses were self-reported, which could lead to recall, social desirability or misclassification biases.
- The survey included women who gave birth within the last 60 days, which likely improved their recall and classification of perinatal compared to late neonatal deaths.

For peer review only

For peer review only - http://bmjopen.bmj.com/site/about/guidelines.xhtml

Background

Since the establishment of the Sustainable Development Goals (SDGs), global health leaders have renewed their pledge to improve maternal and newborn health, with an emphasis on reducing high mortality rates among families who are most socio-economically disadvantaged (1-3). In 2015, 2.7 million infants died in the first month of life, accounting for 45% of child deaths globally. A further 2.6 million were estimated to be stillborn in 2015 (4). Despite considerable progress in the past decades, over one million neonatal deaths and one million stillbirths are estimated to occur every year in South Asia (5). An estimated 28% of neonatal deaths globally occur in India alone (6). The greatest number of deaths happen during the perinatal period, i.e. between 28 completed weeks of gestation and seven days after birth (7, 8). Uttar Pradesh (UP) is India's most populous state and has had the highest perinatal mortality rate (PMR), estimated at 56 per 1000 live births between 2010 to 2015, compared to 36 per 1000 live births nationally (9). Uptake of health services for reproductive, maternal, newborn and child health (RMNCH) has historically been low and inequitable between socio-economic groups in many regions of UP. Nearly 68% of women in UP reported delivering in a health facility in 2015/16. This ranged from 56% among women with no education to over 80% for those with 10 or more standards of education (10, 11).

Community-level interventions are a promising approach to equitably improve maternal and newborn health, with most relying on Community Health Workers (CHWs) (12-14). CHWs have been defined as people who live and work in their communities, are selected by and accountable to them, and may or may not be part of the health system (15-17). The expanded pursuit of primary health care and universal health coverage has given CHW programmes renewed importance in public health programmes (3, 18, 19). Global health leaders have endorsed their potential to improve institutional delivery among communities where resources are most limited (14, 20, 21). There is also evidence that CHWs can promote safe newborn care practices that prevent perinatal mortality (22-26).

India's National Health Mission (NHM) has recruited and trained a large cadre of CHWs called Accredited Social Health Activists (ASHAs) since 2005. ASHAs are trained to keep a birth register of all pregnant women, and routinely contact and counsel women and their families on antenatal, delivery, and postnatal care through home visits. During home visits in the third trimester of a woman's pregnancy, ASHAs are meant to focus on conveying the benefits of institutional delivery, review their birth plan (transportation and place of birth), and counsel on essential newborn care practices, which are all intended to reduce perinatal mortality (27). ASHAs and the women they counsel normally receive monetary incentives

For peer review only - http://bmjopen.bmj.com/site/about/guidelines.xhtml

through the *Janani Suraksha Yojana* scheme for giving birth at a health facility, and these services are meant to be free at public health facilities. ASHAs should inform women about these schemes when they are preparing a birth plan. Their training was also meant to provide guidance on how ASHAs could better reach geographically and socio-economically marginalised families (28). Despite the scale of the ASHA home visiting programme, few studies have examined whether it has improved neonatal health outcomes equitably (as much or more among lower compared to higher socio-economic position groups) (29-31). There is a paucity of research to examine its effects on equity in perinatal mortality overall or comparing between public and private facility births. The present study aimed to examine the extent to which ASHAs' home visits in the third trimester of pregnancy may be associated with equitable improvements in institutional delivery at public and private facilities, and reductions in perinatal mortality rates, between women with lower to higher education levels in 25 districts of Uttar Pradesh state, India.

Methods

Study design and setting

The Government of UP's branch of the NHM has committed substantial resources towards reducing the burden of maternal and neonatal mortality. In 2013, UP's NHM programme established a partnership with the India Health Action Trust, and the University of Manitoba in Canada, under the auspices of the Uttar Pradesh Technical Support Unit (UP TSU), funded by the Bill and Melinda Gates Foundation. This partnership was embedded within UP's NHM programmes in its 25 high priority districts (HPDs), covering a population of over 83 million people (32). The government chose the HPDs by ranking districts using a composite health index that combined six health indicators from the state's Annual Health Survey data (33). This study used data from the first round of UP TSU's community-based, cross-sectional Community Behavioural Tracking Survey (CBTS-1) collected in April 2014 to February 2015, which included 100 blocks (sub-districts) of the 25 HPDs.

Sampling and participants

Sampling was done in two stages, starting with randomly selecting the primary sampling units, and then inviting all eligible women within them to participate. Within the 100 blocks, the CBTS-1 primary sampling unit (PSU) was an ASHA area, which covers approximately 1000 people and is the smallest unit of health service delivery. The required sample size in each block to detect a minimum percentage change of 7.5% in the institutional delivery rate within six months (a key indicator expected to change between survey rounds), assuming an estimated non-response rate of 15% and a design effect of 1.5, was 536 women per

For peer review only - http://bmjopen.bmj.com/site/about/guidelines.xhtml

block. This was considered an ideal sample size given the resources for data collection, compared to what was required for a higher or lower minimum detectable percentage change in institutional delivery rate (5% would give n=1209; 7.5%, n=536; 10%, n=300). The average number of PSUs needed to reach a sample size of at least 536 participants per block was estimated to be 110, assuming that there would be five eligible respondents who had completed a pregnancy within the last 60 days per PSU. This assumption was based on the crude birth rate of UP in the most recent available data (29 per 1000 population in the Annual Health Survey in 2011/12). After all PSUs in the 100 blocks of 25 districts were listed, 110 PSUs were chosen in each of the 100 blocks using systematic random sampling.

In each of the PSUs, every household was enumerated and all eligible women in the household were invited to participate. PSUs were of nearly uniform size. Eligible participants were women aged 15 to 49 who had completed a pregnancy in the past sixty days. 72,054 women were identified and 57,788 were included in the survey, leading to an 80% response rate. There were no refusals to participate; non-response occurred largely when women were repeatedly unavailable at home when the interviewers visited on different days. 5,173 participants responded that their pregnancy ended in an abortion, and therefore were not included in this study's sample. We analysed data from 52,615 participants.

Data collection

Field interviewers' educational background included a minimum of a Bachelor degree in social sciences or related field, and six months or more of related experience. They received ten days of intensive training on data collection methods, including orientation and then hands-on field practice with input from their supervisors. To collect the data, the interviewers first determined the PSU boundary. With a random start, they visited all households and gave each household a number. They used a screening questionnaire in each household to identify women who recently completed a pregnancy, and then described the study to them and asked for their informed consent. The interviewers collected the data anonymously using a mobile application, and the data was routinely quality checked by field supervisors. The survey tool included close-ended questions related to selected background characteristics, pregnancy outcomes, antenatal care, birth preparedness, delivery, postnatal and newborn care, and reproductive health.

Variables

Outcome variables

The outcomes in this study were institutional delivery and perinatal mortality rates. We conducted the analyses with the outcome of institutional delivery (public and private) versus home, as well as for

For peer review only

institutional delivery at a public facility versus home and a private facility versus home separately. This was because of the differing patterns in institutional delivery and PMR at public compared to private facilities based on women's exposure to third trimester home visits and education. PMR was defined as the number of stillbirths (occurring between 28 weeks of gestation and delivery) and deaths during the early neonatal period (first seven days of life) out of 1000 births. These were self-reported and not confirmed by verbal autopsies.

Independent variables

Equity in health has been defined as the absence of systematic disparities in health or its social determinants between groups with differing positions in a social hierarchy (34). A number of socio-economic position characteristics are used to describe inequities in health, in terms of wealth or asset indices, education, occupation, caste, ethnicity and religion (34, 35). Wealth or asset-based inequalities would have been valuable to assess, but this indicator was not available in the first round of the CBTS. We examined caste group differences, but they were more consistent for institutional delivery rates than PMR. We focused on education as the main indicator of socio-economic position in this study as there were consistent inequalities in both outcomes. Higher maternal education has been associated with child survival and utilisation of maternal health services in previous research (36). Education levels represent socio-economic opportunities that are achieved early in life and remain fairly stable over the lifetime, being relatively uninfluenced by later health status. It is also an ordinal variable and enables the examination of gradients in health outcomes (37). We categorised education levels into four groups from least to most years of study (standards) completed based on common Indian school system divisions: none or illiterate, 1 to 5 (lower primary school), 6 to 10 (higher primary to lower secondary), or more than 10 standards (upper secondary and college) (37).

Receipt of ASHA home visits in the third trimester of pregnancy was the main intervention exposure variable. We considered exposure to home visits earlier in pregnancy, but found that only 9.8% of women received a home visit in the first two trimesters of pregnancy but not in the third trimester. This suggested that a third trimester home visit was a good single indicator of ASHA home visit coverage. Third trimester visits are also meant to focus substantially on encouraging and preparing women for institutional delivery.

Covariate and confounding variables

Women's age, caste, religion and parity have been found to be associated with where women give birth, as well as their risk of perinatal loss; all but parity were available in the CBTS-1 data (34-36, 38-40). Having

For peer review only - http://bmjopen.bmj.com/site/about/guidelines.xhtml

any antenatal care visits can increase the likelihood of birth preparedness and relatedly, having an institutional delivery as well (36, 38). Pregnancy, intrapartum and newborn complications have also been found to lead to higher institutional delivery, and these can all affect mortality rates (38). Among these, the available covariates in the dataset that were found to be significant at p<0.05 in bivariable models were retained in multivariable models. Maternal age and caste were adjusted for as confounders in all multivariable models. Religion and any antenatal check-ups were included for institutional delivery, but not PMR, because they were significantly associated with the former but not the latter in the bivariable models. We also adjusted for any pregnancy complications in analyses with both outcomes, as well as intrapartum complications and then place of delivery for perinatal mortality. Pregnancy complications was defined as experiencing any of the following: excessive bleeding before delivery, convulsions, high blood pressure, or sepsis/fever. Intrapartum complications included any of the following: premature labour, preterm/premature rupture of the membrane, prolonged labour of more than 12 hours, obstructed labour, breach/malpresentation, or excessive bleeding immediately after delivery. Conceptual models are shown in Supplementary figures 1 and 2 on the hypothesised relationships between independent variables and each outcome.

Statistical analyses

Analyses were conducted in STATA 13.0. We first used cross-tabulations to describe the independent variables, outcomes and covariates (shown in Table 1). We produced figures to visualise education group differences in the proportion of births at home, public and private facilities, and PMR at each of these locations, by exposure to third trimester home visits (Figures 1 and 2). Then we used bivariable and multivariable generalised linear models (GLM) to assess whether the outcomes were independently associated with having any third trimester home visits. Lastly, we examined whether the associations between the institutional delivery and PMR outcomes and women's exposure to third trimester home visits were differential by education group, and then place of birth using stratified analyses. This was because Figure 1 and 2 suggested that having third trimester home visits may be differentially related to the outcomes depending on women's education level. Interaction terms between exposure to home visits and the outcomes were initially examined. These were significant for institutional delivery in the adjusted models, but not for PMR; the latter showed uneven gradients by education groups that were likely obscured in the interaction term. Thus, we focused on the stratified results as they were more interpretable and transparent about where uneven patterns of inequalities and associations existed.

For peer review only - http://bmjopen.bmj.com/site/about/guidelines.xhtml

In the multivariable analyses with institutional delivery as the outcome (shown in the Table 2), the model was first adjusted for maternal age, caste, religion, any pregnancy complications and any antenatal check-ups. Second, the model was adjusted for the same variables but stratified by education. Thirdly, we ran adjusted models for institutional delivery separately for births at public or private facilities versus home, in relation to women's exposure to home visits overall and then comparing each education group.

In the multivariable models with PMR as the outcome (results in Table 3), all models were adjusted for maternal age, caste, any pregnancy complications and any intrapartum complications. First, we produced the model without place of birth and then adjusted for place of birth. We also stratified the model by education and then by place of birth separately, and finally by education and place of birth at once, to assess how the relationships between PMR and third trimester home visits may be different.

We conducted GLM with a binomial family distribution and a log link function to obtain risk ratios (RR). Some adjusted models with binomial distribution failed to converge for the institutional delivery outcome. Others have found that this can occur when including variables with few data points in some categories, and have addressed this by using a Poisson distribution with robust standard errors to produce the RR (41). Using robust Poisson GLM models with a log link function produced estimates that were almost identical to our analyses using a log binomial distribution in models where convergence did not fail. Therefore, the RR were produced from robust Poisson and log binomial models for institutional delivery and PMR respectively. For both outcomes, we also present the unadjusted and adjusted percentages of institutional delivery and perinatal mortality rates per 1000 live births, alongside the unadjusted (supplementary Table 1 and 2) and adjusted (Tables 2 and 3) RR estimates from the GLM models.

All data collection protocols and analysis plans were approved by the Institutional Review Board of Sigma Research and Consulting in New Delhi [#10040/IRB/D/16-17], and the University College London Research Ethics Committee [#9909/001].

Patient and public involvement statement

No patients or members of the public were involved in this study.

Results

Over 80% of women were 15 to 29 years old (Table 1). The majority of women belonged to Other Backward Class groups, followed by Scheduled Caste and General Caste groups. Nearly 60% of women were illiterate or had no formal education. Over half of the participants (55%) received any home visits

For peer review only - http://bmjopen.bmj.com/site/about/guidelines.xhtml

during the third trimester, and nearly 50% had gone for any ANC check-up. Less than 20% of women reported having any pregnancy complications, while almost 40% reported intrapartum complications.

Table 1: Descriptive socio-demographic and health-related characteristics of the sample

Variable	Total, n (%)	Any third trimester home visit (%)	Institutional delivery			Perinatal mortality rate (per 1000 births)
			Total (%)	Public facility (%)	Private facility (%)	
**Overall**	52615 (100)	55	63	52	11	45
**Age**						
15-24 years	21079 (40)	55	70	56	14	49
25-29 years	21400 (41)	55	62	51	11	39
30+ years	10136 (19)	53	55	47	8	49
**Religion**						
Hindu	42720 (81)	55	64	53	11	45
Muslim/Other	9895 (19)	51	59	46	13	42
**Caste**						
Scheduled Tribe	2216 (4)	40	55	49	6	43
Scheduled Caste	12334 (23)	55	62	54	8	44
Other Backward Class	24579 (47)	55	63	52	11	45
General Caste	6323 (12)	53	72	53	19	48
Don't know	7163 (14)	57	61	49	12	44
**Education**						
None/ illiterate	30614 (58)	52	56	48	8	48
1-5 standards	5693 (11)	57	66	55	11	46
6-10 standards	9895 (19)	58	73	59	14	40
>10 standards	6413 (12)	58	84	60	24	35
**Any pregnancy complications**						
No	43102 (82)	55	63	52	11	38
Yes	9513 (18)	51	67	53	14	76
**Any intrapartum complications**						
No	32000 (61)	55	60	51	9	32
Yes	20615 (39)	53	69	54	15	65

For peer review only - http://bmjopen.bmj.com/site/about/guidelines.xhtml

Variable	Total, n (%)	Any third trimester home visit (%)	Institutional delivery			Perinatal mortality rate (per 1000 births)
			Total (%)	Public facility (%)	Private facility (%)	
**Any ANC check-ups**						
No	27179 (52)	44	54	45	9	45
Yes	25436 (48)	66	73	59	14	45
**Any third trimester home visits**						
No	23914 (45)	NA	54	42	12	50
Yes	28701 (55)	NA	71	61	10	41

Institutional delivery

Almost two-thirds (63%) of women gave birth at a health facility (52% in a public facility, 11% in a private facility). Institutional delivery was higher for women with more education. This ranged from 56% among women who were illiterate or had no formal education to 84% for those with more than 10 standards. Educational inequalities were apparent for both public and private facilities. A greater proportion of women having any versus no third trimester home visits reported giving birth in a public facility (61% vs. 42% respectively). An equal proportion had private facility births without or with any third trimester home visits (12% vs. 10% respectively) (Table 1).

Women who had received any third trimester home visit compared to none reported higher institutional delivery overall (71% vs. 54%) (RR 1.31 [95%CI: 1.29, 1.33]) (supplementary table 1). This association remained significant after adjusting for confounders (aRR 1.23 [95%CI: 1.22, 1.25]) (Table 2). The adjusted associations between having an institutional birth and any third trimester home visit was stronger with each step down the educational gradient.

[Figure 1]

Figure 1 illustrates that a higher proportion of women gave birth at public facilities versus home when they had any compared to no third trimester visits from an ASHA, and this was most noticeable among the least educated groups. In Table 2, the adjusted association between having any third trimester home visit and an institutional birth was stronger for births at a public facility (aRR 1.32 [95% CI: 1.30-1.34]) than those at a private facility (aRR 1.08 [95% CI: 1.04-1.13]) compared to home. This was observed for all educational groups, but particularly so for less educated women.

For peer review only - http://bmjopen.bmj.com/site/about/guidelines.xhtml

Table 2: Associations between institutional delivery and any third trimester home visits, overall and by education level

Third trimester home visits	All education groups (n=52615)		None / illiterate (n= 30614)		1-5 standards (n=5693)		6-10 standards (n=9895)		>10 standards (n=6413)	
	Adjusted % (95% CI)	Adjusted RR (95% CI)	Adjusted % (95% CI)	Adjusted RR (95% CI)	Adjusted % (95% CI)	Adjusted RR (95% CI)	Adjusted % (95% CI)	Adjusted RR (95% CI)	Adjusted % (95% CI)	Adjusted RR (95% CI)
Institution (public and private) vs. home										
None (n=23914)	54.2 (53.6, 54.9)	Ref	47.2 (46.4, 48.0)	Ref	57.5 (55.6, 59.5)	Ref	67.2 (65.7, 68.6)	Ref	81.6 (80.1, 83.1)	Ref
Any (n=28701)	71.1 (70.6, 71.6)	1.23 (1.22, 1.25)	62.9 (62.2, 63.6)	1.33 (1.30, 1.36)	71.3 (69.7, 72.8)	1.24 (1.19, 1.29)	77.3 (76.2, 78.4)	1.15 (1.12, 1.18)	85.6 (84.4, 86.7)	1.05 (1.03, 1.07)
Public facility vs. home										
None (n=20997)	47.9 (47.2, 48.9)	Ref	42.4 (41.5, 43.2)	Ref	51.5 (49.4, 53.7)	Ref	60.6 (58.9, 72.9)	Ref	74.1 (72.1, 76.1)	Ref
Any (n=25682)	67.7 (67.1, 68.3)	1.32 (1.30, 1.34)	59.9 (59.2, 60.7)	1.41 (1.38, 1.45)	68.4 (66.7, 70.1)	1.33 (1.26, 1.39)	74.1 (72.9, 75.3)	1.22 (1.18, 1.26)	81.9 (80.5, 83.3)	1.11 (1.07, 1.14)
Private facility vs. home										
None (n=13860)	22.6 (22.0, 23.3)	Ref	13.7 (13.0, 14.4)	Ref	22.8 (20.6, 25.0)	Ref	33.8 (31.8, 35.9)	Ref	61.4 (58.9, 64.0)	Ref
Any (n=11318)	24.6 (23.9, 25.3)	1.09 (1.04, 1.13)	16.6 (15.8, 17.5)	1.21 (1.12, 1.31)	23.9 (21.6, 26.2)	1.05 (0.91, 1.20)	35.1 (33.1, 37.1)	1.04 (0.95, 1.13)	58.0 (55.3, 60.6)	0.94 (0.87, 1.00)

For peer review only - http://bmjopen.bmj.com/site/about/guidelines.xhtml

Perinatal mortality rate

The overall perinatal mortality rate in the CBTS-1 sample was 45 per 1000 births (Table 1). The greatest number of early neonatal deaths occurred on the day of birth, and steadily decreased in number on subsequent days (supplementary Figure 3). Perinatal mortality reduced with increasing educational attainment, from 48 to 35 per 1000 births among women with no education compared to more than ten standards respectively. The PMR was 39 per 1000 births among women giving birth at home, 43 per 1000 births at public facilities, and 73 per 1000 births at private facilities overall.

The PMR was higher among women without compared to with any third trimester home visit, at 50 compared to 41 per 1000 births respectively (RR 1.21 [95% CI: 1.12, 1.31]) (supplementary Table 2). The association remained after adjustment for socio-demographic characteristics, any pregnancy and intrapartum complications (aRR 1.17 [95% CI: 1.98, 1.27]), and after additional adjustment for place of birth (aRR 1.18 [95% CI: 1.09, 1.28]) (Table 3).

The association between having no third trimester home visits and higher PMR was stronger for those with one to five standards of education (aRR 1.53 [95% CI: 1.20, 1.94]), and to a lesser extent 5 to 10 standards (aRR 1.27 [95% CI: 1.05, 1.54]). The association was somewhat weaker for women with no education (aRR 1.13 [95% CI: 1.02, 1.25]), and absent among women who had an education over ten standards.

The inverse association between any third trimester home visits and PMR was stronger for births at public facilities (aRR 1.31 [95% CI: 1.17, 1.47]). There was no association for births at private facilities (aRR 1.12 [95% CI: 0.93, 1.34]) nor for home births (aRR 1.04 [95% CI: 0.91, 1.21]).

[Figure 2]

Figure 2 shows that for women who had any versus no third trimester home visits, educational differences in PMR appeared to be smaller among public facility births and to a lesser extent also for private facility births. In adjusted analyses, PMR was significantly higher among those without third trimester home visits compared to those with any visits for public facility births for all except the most educated group (Table 3). However, differences in PMR between women with and without third trimester home visits were not significant for those who delivered at home or in private facilities, across education groups. Confidence intervals were wide in some of the comparisons based on lower numbers of perinatal deaths.

For peer review only - http://bmjopen.bmj.com/site/about/guidelines.xhtml

Table 3: Adjusted associations between perinatal mortality rate and any third trimester home visits, overall and by education and place of birth

Third trimester home visits	All education groups (n=52588)		None / illiterate (n= 30600)		1-5 standards (n=5689)		6-10 standards (n=9887)		>10 standards (n=6412)	
	Adjusted PMR (95% CI)	Adjusted RR (95% CI)	Adjusted PMR (95% CI)	Adjusted RR (95% CI)	Adjusted PMR (95% CI)	Adjusted RR (95% CI)	Adjusted PMR (95% CI)	Adjusted RR (95% CI)	Adjusted PMR (95% CI)	Adjusted RR (95% CI)
All births, not adjusting for place of birth										
Any (n=28686)	41.5 (39.2, 43.8)	Ref	45.9 (42.7, 49.2)	Ref	37.8 (21.2, 44.3)	Ref	35.6, 30.8, 40.4)	Ref	33.1 (27.3, 38.9)	Ref
None (n=23902)	48.6 (45.9, 51.3)	1.17 (1.08, 1.27)	50.7 (47.2, 54.2)	1.10 (1.00, 1.22)	56.1 (47.0, 65.1)	1.48 (1.17, 1.88)	46.3 (40.0, 52.6)	1.30 (1.07, 1.58)	36.3 (29.3, 43.3)	1.10 (0.84, 1.42)
All births, adjusting for place of birth										
Any (n=28686)	41.3 (39.0, 43.6)	Ref	45.5 (42.2, 48.7)	Ref	37.3 (30.8, 43.8)	Ref	36.0 (31.1, 40.9)	Ref	33.3 (27.5, 39.2)	Ref
None (n=23902)	48.9 (46.1, 51.6)	1.18 (1.09, 1.28)	51.2 (47.7, 54.8)	1.13 (1.02, 1.25)	56.9 (47.7, 66.2)	1.53 (1.20, 1.94)	45.7 (39.4, 52.0)	1.27 (1.05, 1.54)	35.9 (28.9, 42.9)	1.07 (0.83, 1.40)
Home delivery										
Any (n=8295)	37.8 (33.7, 42.0)	Ref	41.1 (35.8, 46.4)	Ref	23.7 (13.7, 33.7)	Ref	35.5 (25.1, 45.9)	Ref	30.0 (14.9, 45.1)	Ref
None (n=10935)	39.7 (36.1, 43.3)	1.04 (0.91, 1.21)	41.6 (37.2, 45.9)	1.01 (0.86, 1.19)	35.4 (24.4, 46.5)	1.50 (0.88, 2.53)	38.0 (28.2, 47.8)	1.07 (0.72, 1.58)	26.3 (12.6, 40.0)	0.88 (0.42, 1.82)
Public facility delivery										
Any (n=17426)	38.4 (35.5, 41.3)	Ref	43.8 (39.6, 48.0)	Ref	35.6 (27.6, 43.6)	Ref	31.8 (26.2, 37.4)	Ref	30.4 (23.6, 37.2)	Ref
None (n=10052)	50.4 (46.2, 54.6)	1.31 (1.17, 1.47)	54.5 (48.5, 60.4)	1.24 (1.08, 1.44)	62.7 (48.3, 43.6)	1.76 (1.28, 2.43)	44.0 (35.3 (52.8)	1.38 (1.06, 1.81)	35.3 (25.7, 44.9)	1.16 (0.82, 1.65)
Private facility delivery										
Any (n=3017)	68.5 (59.6, 77.4)	Ref	84.8 (69.6, 100.0)	Ref	96.0 (62.4, 129.6)	Ref	56.9 (40.0, 73.8)	Ref	43.5 (29.0, 58.0)	Ref
None (n=2915)	76.5 (67.0, 86.1)	1.12 (0.93, 1.34)	93.1 (76.6, 109.5)	1.10 (0.85, 1.41)	104.5 (70.8, 138.8)	1.09 (0.68, 1.76)	69.4 (50.7, 88.0)	1.22 (0.82, 1.82)	45.5 (30.8, 60.2)	1.05 (0.66, 1.66)

For peer review only - http://bmjopen.bmj.com/site/about/guidelines.xhtml

Discussion

This study sought to examine the associations between women's exposure to ASHAs' third trimester home visits and education-based inequalities in institutional delivery and perinatal mortality rates in 25 districts of Uttar Pradesh, India. The results of this study indicate that having any ASHA home visits during women's third trimester of pregnancy was associated with giving birth in public facilities, and this association was increasingly strong for decreasing education levels. Perinatal mortality rates were found to be highest for those delivering at private facilities, and especially among the least educated women. Women's receipt of any ASHA home visit in the third trimester was significantly associated with lower PMR, and most strongly for women giving birth in public facilities among all but the most educated.

There were some limitations to the analyses. The CBTS-1 survey responses were self-reported, and therefore prone to recall, misclassification and social desirability biases. The survey included women who delivered in the past 60 days to improve recall. However, the response rate was reduced somewhat because some women were not yet home from the facility or went to their maternal home in this period. The measure of an 'early' neonatal death could have been over- or under-reported, though combining stillbirths and early neonatal deaths to measure PMR can avoid the common misclassification of early neonatal deaths as stillbirths. We did not observe heaping of reported neonatal deaths within the first six days after birth, and the proportion of neonatal deaths at various times after delivery was similar across education groups. The CBTS-1 offered a sufficiently large population-based sample to measure PMR, and to stratify the analyses by education and place of delivery. However, its purpose as a programme monitoring methodology resulted in lack of data on some relevant factors or confounders, such as wealth, parity, newborn's sex, or having a singleton or twin birth that might have attenuated the strength of observed associations if included (42). The cross-sectional nature of the analysis precluded conclusions about causality. Moreover, we assumed that the associations between exposure and outcome variables could involve reverse or multidirectional, rather than linear, pathways of causation (43). For example, ASHAs may have more often chosen to visit women who preferred public facility births as a way to receive their incentives, while women with lower education may more often opt for the less expensive public services. The results may not be directly generalisable to non-HPD districts of UP, where institutional delivery was likely higher and PMR lower, and possibly less unequal. It may be less relevant to other states where there are different roles for ASHAs, or types of facilities providing RMNCH services, and these may have also changed over time.

For peer review only - http://bmjopen.bmj.com/site/about/guidelines.xhtml

Our results suggested that having any third trimester home visits from an ASHA was associated with a greater chance of women having an institutional delivery, and particularly at public facilities. Further, having any third trimester home visits was more greatly associated with having an institutional delivery for mothers with lower education levels. Other studies have reported positive associations between exposure to community-based interventions and lower socio-economic inequalities in institutional delivery and skilled birth attendance in UP. Another cross-sectional study found that having any ASHA home visit was associated with higher institutional delivery among poorer and illiterate women, but not among richer or more educated women (30). A quasi-experimental study of an intervention involving NGO technical support to the ASHA programme in two districts of UP found that skilled birth attendance became more equitable between wealth groups in the intervention district but not the comparison district (29). The role of ASHAs' home visits in increasing the proportion of institutional deliveries, and particularly at public facilities, among women with less education and to a lesser extent more education may be due to their role in promoting the free public health services and incentives within the National Health Mission. Studies have found that ASHAs have generally had a strong role in encouraging women to attend public antenatal and delivery care, in part by promoting the *Janani Suraksha Yojana* incentives, and that this has occurred alongside shifting social norms in favour of facility-based maternity care in India (44-47). Our findings seem to support evidence indicating that having at least one home visit in the third trimester may contribute to improving institutional delivery not only overall but equitably, by facilitating access for less educated women to the more affordable care in the public health system in UP. It would be valuable for future research to examine whether having multiple home visits leads to even better outcomes, and to compare this for women of lower and higher age and birth order who tend to be at greater risk of perinatal loss.

This study indicated that ASHAs' home visits played the strongest role in supporting women of lower education groups to give birth at public facilities. Yet women from the lower, and even higher, education groups who delivered at private facilities still had high perinatal mortality rates, though there were fewer cases leading to wider confidence intervals. Researchers have discussed how improvements in institutional delivery have not always translated into reductions in mortality among mothers and their babies where there has been lower quality of services and poor referral mechanisms, particularly at primary health care facilities or unregulated private services where people of lower socio-economic positions often attend first (48, 49). More research is needed to understand the processes by which inequitably high perinatal mortality rates continue for women of lower socio-economic positions,

For peer review only - http://bmjopen.bmj.com/site/about/guidelines.xhtml

especially those who face pregnancy and intrapartum complications, such as the barriers to reaching higher-level public and especially private health services in time or at all.

In 2018, the Government of India launched the *Ayushman Bharat* Programme (ABP) to enhance the availability of free integrated primary health care services through village-level Health and Wellness Centers (HWCs) (50). The programme aims to attach five ASHAs to each HWC, where they will work with new mid-level health providers. ASHAs are to continue home visits to promote utilisation and safe health behaviours for RMNCH, among a range of other health services (50). One systematic review of previous studies in India and elsewhere found that lower neonatal mortality was associated with CHW home visits, particularly when they included both preventive (e.g. counselling on care seeking or behaviours) and curative roles (e.g. provision of injectable antibiotics) (51). Another showed that the effects of CHW home visits on care-seeking for institutional delivery can be equal or greater for lower compared to higher wealth or education groups (52). It will be crucial to support ASHAs to continue providing counselling on institutional delivery, essential newborn care, as well as timely identification, treatment or referral of pregnancy or newborn complications for women that also face greater vulnerability due to socio-economic disadvantage (3, 50, 53, 54).

Building on the NHM, the ABP policies are also explicitly based on the premise that the health system functions effectively (50). The ABP includes public as well as empanelled private first referral units to be covered by the insurance scheme called *Pradhan Mantri Jan Arogya Yojana*, which should remove financial barriers for women of lower socio-economic groups facing birth complications (55). As more women and their families are giving birth at public and private health facilities, it is particularly important to consider the pluralistic health system in India holistically and in context when addressing maternal and perinatal health inequities (49, 55-57). This would necessitate targeting the causes of differential gaps in accessibility, quality and integration of these health sectors, and between community and facility-based services, so as to strategically address persistent inequalities in perinatal mortality.

Conclusions

This study's findings suggested that ASHAs' contacts and counselling for pregnant women through home visits in the third trimester may have influenced them to give birth at a public facility, and particularly those with lower compared to higher education levels. Further, less educated women who had any compared to no home visits that delivered at a public facility appeared to have significantly lower rates of perinatal mortality. Future research, policies, and programmes should seek to understand the processes

For peer review only - http://bmjopen.bmj.com/site/about/guidelines.xhtml

by which community health workers' home visits contributed to improving equity in perinatal health outcomes, and how more integrated community and health system efforts can strategically support these efforts.

Funding

This work was supported by the Bill & Melinda Gates Foundation [OPP1161429]. AKB's time was funded by University College London's Overseas and Graduate Research Scholarships. TAJH's time was funded by a Research Excellence Initiative grant from Erasmus University Rotterdam, The Netherlands. The funders had no role in the design of the study, data collection and analysis, planning or writing of the manuscript.

Contributor statement

AKB planned and conducted the analyses, and wrote the draft manuscript. TAJH, TC and AP provided guidance on the design of the study and support throughout the analyses. BMR, SI, JA, and BD designed the CBTS methodology, managed the data collection and quality assurance, and gave technical input on the analyses. All authors read and gave input on the manuscript.

Competing interests

The authors declare no competing interests.

Data sharing statement

The data are hosted on the University of Manitoba server, and a de-identified data cut along with the questionnaires and data dictionary may be made available upon reasonable request (contact e-mail: Ramesh.BanadakoppaManjappa@umanitoba.ca).

Figures

Figure 1: Proportion of women in each education group giving birth at home, public facilities, or private facilities, by exposure to any third trimester home visits

Figure 2: PMR among women in each education group, by place of birth and exposure to any third trimester home visits

Bibliography

1. Houweling TAJ, Morrison J, Azad K, Manandhar DS, Alcock G, Shende S, et al. How to reach every newborn: Three key messages. The Lancet Global Health. 2014;2(8):e436-e7. Available from: doi:10.1016/S2214-109X(14)70271-2.

For peer review only - http://bmjopen.bmj.com/site/about/guidelines.xhtml

2. Requejo JH, Bryce J, Barros AJ, Berman P, Bhutta Z, Chopra M, et al. Countdown to 2015 and beyond: fulfilling the health agenda for women and children. Lancet. 2015;385(9966):466-76. Available from: doi:10.1016/S0140-6736(14)60925-9.

3. Boerma T, Requejo J, Victora CG, Amouzou A, George A, Agyepong I, et al. Countdown to 2030: tracking progress towards universal coverage for reproductive, maternal, newborn, and child health. The Lancet. 2018;391(10129):1538-48. Available from: doi:10.1016/S0140-6736(18)30104-1.

4. You D, Hug, L, Ejdemyr, S, Beise, J. Levels and Trends in Child Mortality Report 2015. Geneva: Inter-Agency Group for Child Mortality Estimation, 2015.

5. Akseer N, Kamali M, Arifeen SE, Malik A, Bhatti Z, Thacker N, et al. Progress in maternal and child health: how has South Asia fared? Bmj. 2017;357:j1608. Available from: doi:10.1136/bmj.j1608.

6. Black RE, R. Laxminarayan, M. Temmerman, and N. Walker. (eds.) Reproductive, Maternal, Newborn, and Child Health. Disease Control Priorities. 3 ed. Washington, DC: World Bank; 2016.

7. United Nations Children Fund. Neonatal Mortality. Geneva: UNICEF; 2018 [cited 2018 March 12]. Available from: https://data.unicef.org/topic/child-survival/neonatal-mortality/

8. World Health Organization. Maternal and perinatal health. Geneva: World Health Organization; 2018 [cited 2018 March 12]. Available from: http://www.who.int/maternal_child_adolescent/topics/maternal/maternal_perinatal/en/.

9. International Institute for Population Sciences (IIPS) and ICF. National Family Health Survey (NFHS-4), 2015-16: India. Mumbai: Ministry of Health and Family Welfare, 2017.

10. Dettrick Z, Jimenez-Soto E, Hodge A. Socioeconomic and geographical disparities in under-five and neonatal mortality in Uttar Pradesh, India. Maternal and Child Health Journal. 2014;18(4):960-9. Available from: doi:10.1007/s10995-013-1324-8.

11. International Institute for Population Sciences (IIPS) and ICF. National Family Health Survey (NFHS-4), India, 2015-16: Uttar Pradesh. Mumbai: 2017.

12. Bhutta ZA, Das JK, Rizvi A, Gaffey MF, Walker N, Horton S, et al. Evidence-based interventions for improvement of maternal and child nutrition: what can be done and at what cost? Lancet. 2013;382(9890):452-77. Available from: doi:10.1016/s0140-6736(13)60996-4.

13. Ekman B, Pathmanathan I, Liljestrand J. Integrating health interventions for women, newborn babies, and children: a framework for action. Lancet. 2008;372(9642):990-1000. Available from: doi:10.1016/S0140-6736(08)61408-7.

14. Bhutta ZA, Black RE. Global maternal, newborn, and child health - So near and yet so far. New England Journal of Medicine. 2013;369(23):2226-35. Available from: doi:10.1056/NEJMra1111853.

15. Olaniran A, Smith H, Unkels R, Bar-Zeev S, van den Broek N. Who is a community health worker? – a systematic review of definitions. Glob Health Action. 2017;10(1):1-13. Available from: doi:10.1080/16549716.2017.1272223.

For peer review only

For peer review only - http://bmjopen.bmj.com/site/about/guidelines.xhtml

16. World Health Organization. Community health workers: a strategy to ensure access to primary health care services. Cairo: Regional Office for the Eastern Mediterranean, 2016.

17. Tulenko K, Mogedal S, Afzal MM, Frymus D, Oshin A, Pate M, et al. Community health workers for universal health-care coverage: from fragmentation to synergy. Bull World Health Organ. 2013;91(11):847-52. Available from: doi:10.2471/BLT.13.118745.

18. Perry H. A Brief History of Community Health Worker Programs. In: Perry H, Crigler L. (eds.) Developing and Strengthening Community Health Worker Programs at Scale - A Reference Guide and Case Studies for Program Managers and Policymakers. Washington: United States Agency for International Development; 2014. p. 1-14.

19. The Lancet. The Astana Declaration: the future of primary health care? The Lancet. 2018;392(10156):1369. Available from: doi:10.1016/S0140-6736(18)32478-4.

20. Victora CG, Wagstaff A, Schellenberg JA, Gwatkin D, Claeson M, Habicht J-P. Applying an equity lens to child health and mortality: more of the same is not enough. The Lancet. 2003;362(9379):233-41. Available from: doi:https://doi.org/10.1016/S0140-6736(03)13917-7.

21. Bhutta ZA, Reddy KS. Achieving equity in global health: So near and yet so far. JAMA - Journal of the American Medical Association. 2012;307(19):2035-6. Available from: doi:10.1001/jama.2012.4659.

22. Gilmore B, McAuliffe E. Effectiveness of community health workers delivering preventive interventions for maternal and child health in low- and middle-income countries: a systematic review. BMC public health. 2013;13(847):1-14.

23. Gogia S, Sachdev HS. Home visits by community health workers to prevent neonatal deaths in developing countries: a systematic review. Bulletin of the World Health Organization. 2010;88(9):658-66B. Available from: doi:http://dx.doi.org/10.2471/BLT.09.069369.

24. Prost A, Pagel C, Costello A. Community interventions to reduce maternal and child mortality in low income countries. Maternal and Infant Deaths: Chasing Millennium Development Goals 4 and 52010. p. 205-16.

25. Haines A, Sanders D, Lehmann U, Rowe AK, Lawn JE, Jan S, et al. Achieving child survival goals: potential contribution of community health workers. Lancet. 2007;369(9579):2121-31. Available from: doi:10.1016/s0140-6736(07)60325-0.

26. Lassi ZS, Bhutta ZA. Community-based intervention packages for reducing maternal and neonatal morbidity and mortality and improving neonatal outcomes. Cochrane Database Syst Rev. 2015(3):1-78. Available from: doi:10.1002/14651858.CD007754.pub3.

27. Ministry of Health and Family Welfare. Guidelines for Community Processes - National Rural Health Mission. New Delhi: Government of India, 2014.

28. Ministry of Health and Family Welfare. ASHA Training Modules - National Health Mission. Delhi: Government of India; 2014 [cited 2017 March 28]. Available from: http://nrhm.gov.in/communitisation/asha/resources/asha-training-modules.html.

For peer review only

For peer review only - http://bmjopen.bmj.com/site/about/guidelines.xhtml

29. Baqui AH, Rosecrans AM, Williams EK, Agrawal PK, Ahmed S, Darmstadt GL, et al. NGO facilitation of a government community-based maternal and neonatal health programme in rural India: improvements in equity. Health Policy Plan. 2008;23(4):234-43. Available from: doi:http://dx.doi.org/10.1093/heapol/czn012.

30. Seth A, Tomar S, Singh K, Chandurkar D, Chakraverty A, Dey A, et al. Differential effects of community health worker visits across social and economic groups in Uttar Pradesh, India: a link between social inequities and health disparities. International Journal for Equity in Health. 2017;16(46):1-9. Available from: doi:10.1186/s12939-017-0538-6.

31. Rajbhandari S, Hodgins S, Sanghvi H, McPherson R, Pradhan YV, Baqui AH, et al. Expanding uterotonic protection following childbirth through community-based distribution of misoprostol: operations research study in Nepal. Int J Gynaecol Obstet. 2010;108(3):282-8. Available from: doi:http://dx.doi.org/10.1016/j.ijgo.2009.11.006.

32. Bill and Melinda Gates Foundation. Maternal, Newborn and Child Health. Seattle: Bill and Melinda Gates Foundation; 2014 [cited 2017 December 12]. Available from: http://www.gatesfoundation.org/What-We-Do/Global-Development/Maternal-Newborn-and-Child-Health.

33. Ministry of Health and Family Welfare. Guidance note for implementation of RMNCH+A interventions in High Priority Districts. New Delhi: Government of India, 2013.

34. Braveman P, Gruskin S. Defining equity in health. J Epidemiol Commun Health. 2003;57:254-8. Available from: doi:10.1136/jech.57.4.254.

35. Marmot M. Closing the health gap in a generation: the work of the Commission on Social Determinants of Health and its recommendations. Glob Health Promot. 2009;Suppl 1:23-7. Available from: doi:10.1177/1757975909103742.

36. Gabrysch S, Campbell OM. Still too far to walk: literature review of the determinants of delivery service use. BMC Pregnancy Childbirth. 2009;9(34). Available from: doi:10.1186/1471-2393-9-34.

37. Solar O, Irwin A. A conceptual framework for action on the social determinants of health. Geneva: World Health Organization, 2010.

38. Lawn JE, Blencowe H, Oza S, You D, Lee AC, Waiswa P, et al. Every Newborn: progress, priorities, and potential beyond survival. Lancet. 2014;384(9938):189-205. Available from: doi:10.1016/S0140-6736(14)60496-7.

39. Ministry of Health and Family Welfare. India Newborn Action Plan. New Delhi: 2014.

40. Sankar MJ, Neogi SB, Sharma J, Chauhan M, Srivastava R, Prabhakar PK, et al. State of newborn health in India. Journal of Perinatology. 2016;36(Suppl 3):S3-S8. Available from: doi:10.1038/jp.2016.183.

41. Zou G. A Modified Poisson Regression Approach to Prospective Studies with Binary Data. American Journal of Epidemiology. 2004;159(7):702-6. Available from: doi:10.1093/aje/kwh090.

For peer review only - http://bmjopen.bmj.com/site/about/guidelines.xhtml

42. Barros AJD, Victora CG. Measuring Coverage in MNCH: Determining and Interpreting Inequalities in Coverage of Maternal, Newborn, and Child Health Interventions. PLoS Med. 2013;10(5):e1001390. Available from: doi:10.1371/journal.pmed.1001390.

43. VanderWeele TJ. Mediation Analysis: A Practitioner's Guide. Annu Rev Public Health. 2016;37:17-32. Available from: doi:10.1146/annurev-publhealth-032315-021402.

44. Scott K, George AS, Ved RR. Taking stock of 10 years of published research on the ASHA programme: examining India's national community health worker programme from a health systems perspective. Health Research Policy and Systems. 2019;17(1):29. Available from: doi:10.1186/s12961-019-0427-0.

45. Sidney K, Tolhurst R, Jehan K, Diwan V, De Costa A. 'The money is important but all women anyway go to hospital for childbirth nowadays' - a qualitative exploration of why women participate in a conditional cash transfer program to promote institutional deliveries in Madhya Pradesh, India. BMC Pregnancy and Childbirth. 2016;16(1):47. Available from: doi:10.1186/s12884-016-0834-y.

46. Bruce SG, Blanchard AK, Gurav K, Roy A, Jayanna K, Mohan HL, et al. Preferences for infant delivery site among pregnant women and new mothers in Northern Karnataka, India. BMC Pregnancy Childbirth. 2015;15(49):1-10. Available from: doi:10.1186/s12884-015-0481-8.

47. Lim SS, Dandona L, Hoisington JA, James SL, Hogan MC, Gakidou E. India's Janani Suraksha Yojana, a conditional cash transfer programme to increase births in health facilities: an impact evaluation. Lancet. 2009;375(9730):2009-23. Available from: doi:10.1016/s0140-6736(10)60744-1.

48. Sharma J, Leslie HH, Regan M, Nambiar D, Kruk ME. Can India's primary care facilities deliver? A cross-sectional assessment of the Indian public health system's capacity for basic delivery and newborn services. BMJ Open. 2018;8(6):e020532. Available from: doi:10.1136/bmjopen-2017-020532.

49. McPake B, Hanson K. Managing the public & private mix to achieve universal health coverage. The Lancet. 2016;388(10044):622-30. Available from: doi:10.1016/S0140-6736(16)00344-5.

50. Ministry of Health and Family Welfare. Ayushman Bharat: Comprehensive Primary Health Care through Health and Wellness Centers Operational Guidelines. New Delhi: Government of India, 2018.

51. Gogia S, Sachdev HS. Home visits by community health workers to prevent neonatal deaths in developing countries: A systematic review. Bulletin of the World Health Organization. 2010;88(9):658-66. Available from: doi:10.2471/BLT.09.069369.

52. Blanchard AK, Prost A, Houweling TAJ. Effects of community health worker interventions on socioeconomic inequities in maternal and newborn health in low-income and middle-income countries: a mixed-methods systematic review. Bmj Global Health. 2019;4(3). Available from: doi:10.1136/bmjgh-2018-001308.

53. Barros AJ, Ronsmans C, Axelson H, Loaiza E, Bertoldi AD, Franca GV, et al. Equity in maternal, newborn, and child health interventions in Countdown to 2015: a retrospective review of survey data from 54 countries. Lancet. 2012;379(9822):1225-33. Available from: doi:10.1016/S0140-6736(12)60113-5.

For peer review only - http://bmjopen.bmj.com/site/about/guidelines.xhtml

54. Black RE, Levin C, Walker N, Chou D, Liu L, Temmerman M. Reproductive, maternal, newborn, and child health: key messages from Disease Control Priorities 3rd Edition. The Lancet. 2016;388(10061):2811-24. Available from: doi:http://dx.doi.org/10.1016/S0140-6736(16)00738-8.

55. Angell BJ, Prinja S, Gupt A, Jha V, Jan S. The Ayushman Bharat Pradhan Mantri Jan Arogya Yojana and the path to universal health coverage in India: Overcoming the challenges of stewardship and governance. PLOS Medicine. 2019;16(3):e1002759. Available from: doi:10.1371/journal.pmed.1002759.

56. Upadhyay RP, Krishnan A, Rai SK, Chinnakali P, Odukoya O. Need to Focus Beyond the Medical Causes: a Systematic Review of the Social Factors Affecting Neonatal Deaths. Paediatr Perinat Epidemiol. 2014;28(2):127-37. Available from: doi:10.1111/ppe.12098.

57. Wall SN, Lee AC, Carlo W, Goldenberg R, Niermeyer S, Darmstadt GL, et al. Reducing intrapartum-related neonatal deaths in low- and middle-income countries-what works? Semin Perinatol. 2010;34(6):395-407. Available from: doi:10.1053/j.semperi.2010.09.009.

For peer review only

For peer review only - http://bmjopen.bmj.com/site/about/guidelines.xhtml

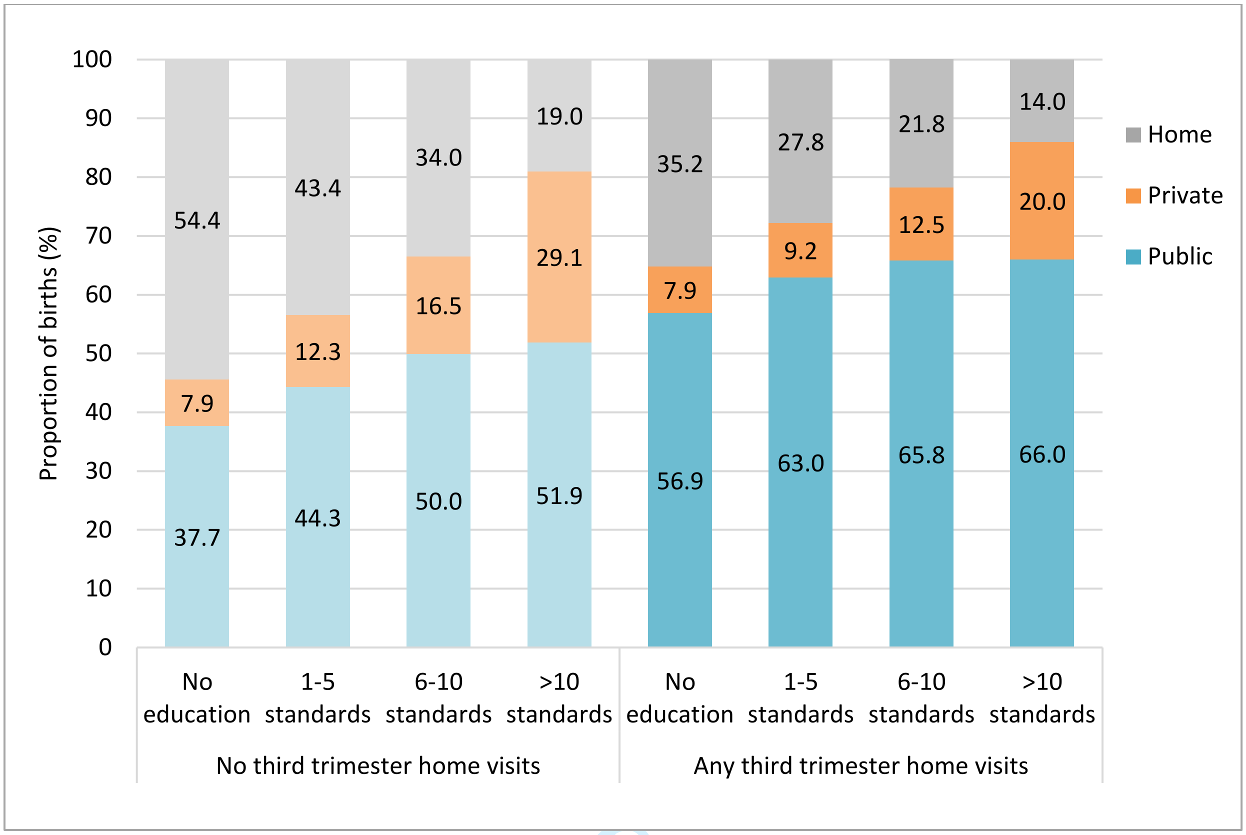

Figure 1: Proportion of women in each education group giving birth at home, public facilities, or private facilities, by exposure to any third trimester home visits

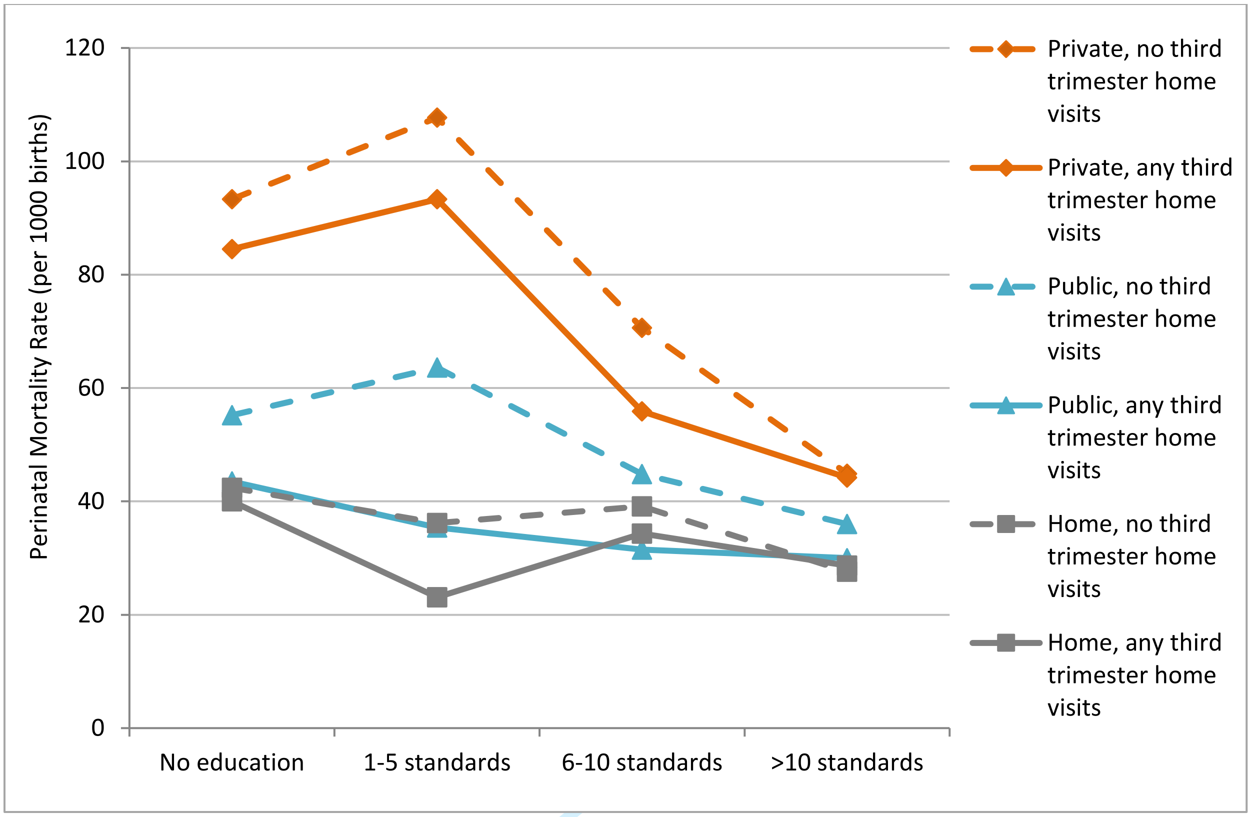

Figure 2: PMR among women in each education group, by place of birth and exposure to any third trimester home visits

For peer review only

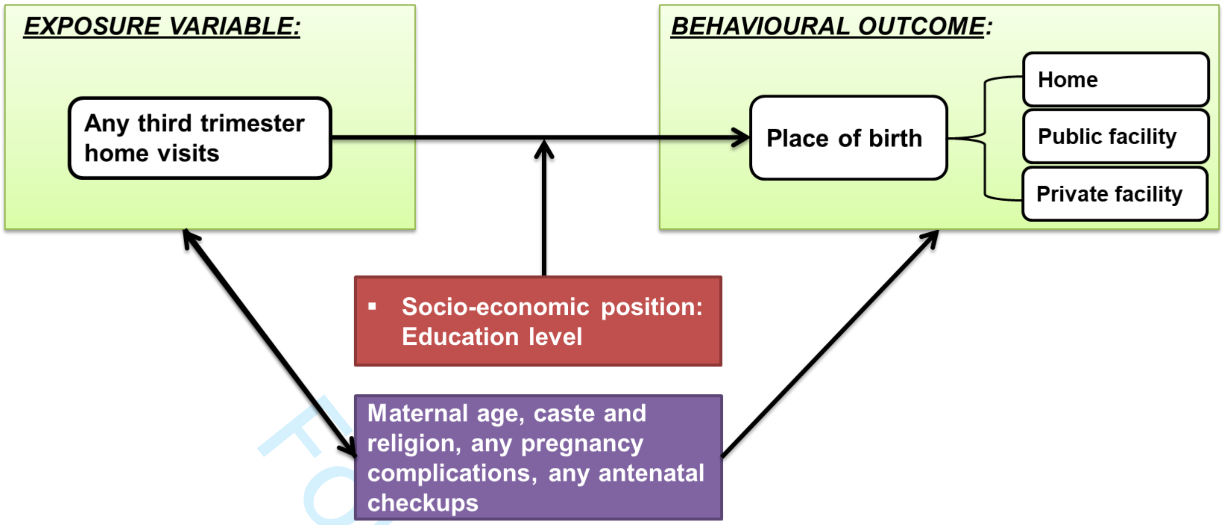

Supplementary figure 1: Conceptual model of the associations between women's exposure to third trimester home visits and place of birth

For peer review only

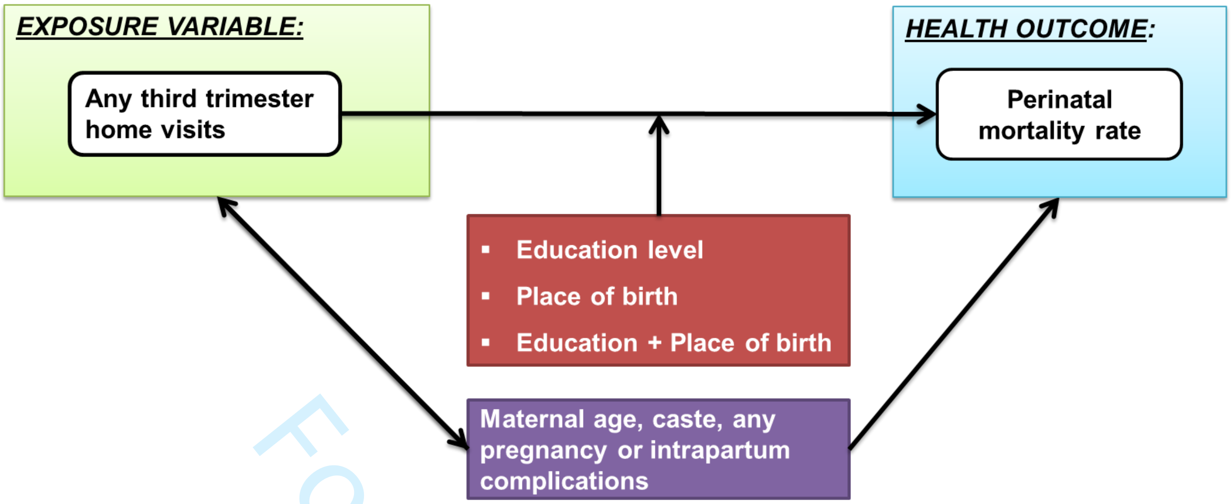

Supplementary figure 2: Conceptual model of the associations between women's exposure to third trimester home visits and perinatal mortality

For peer review only

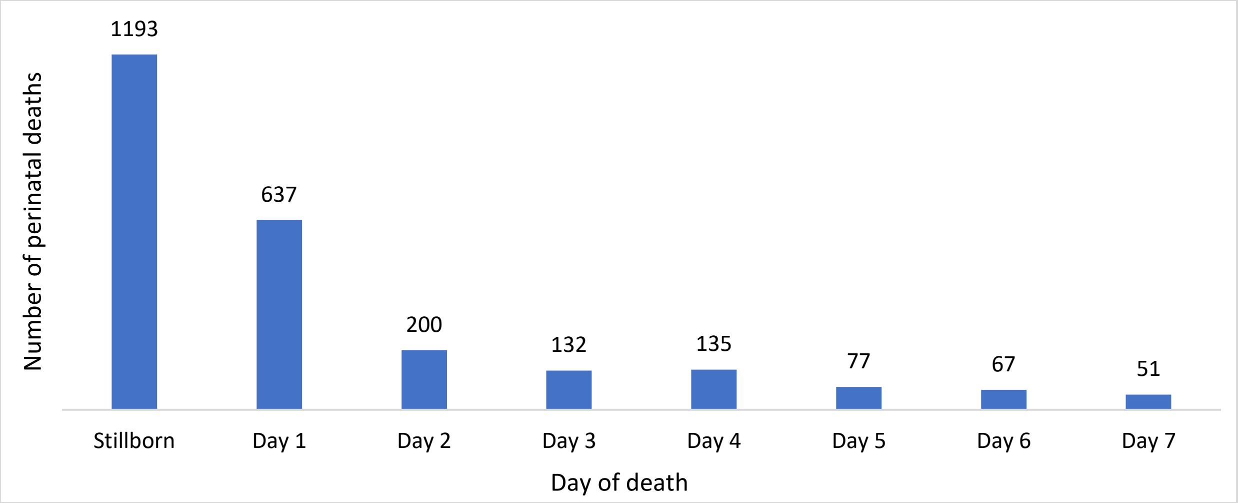

Supplementary figure 3: Distribution and timing of perinatal deaths among women who completed a pregnancy in the past two months

For peer review only

Supplementary table 1: Unadjusted associations between institutional delivery and any third trimester home visits, overall and by education level

Third trimester home visits	All education groups (n=52615)		None / illiterate (n= 30614)		1-5 standards (n=5693)		6-10 standards (n=9895)		>10 standards (n=6413)	
	Unadjusted % (95% CI)	Unadjusted RR (95% CI)	Unadjusted % (95% CI)	Unadjusted RR (95% CI)	Unadjusted % (95% CI)	Unadjusted RR (95% CI)	Unadjusted % (95% CI)	Unadjusted RR (95% CI)	Unadjusted % (95% CI)	Unadjusted RR (95% CI)
Institution (public and private) vs. home										
None (n=23914)	54.2 (53.6, 54.9)	Ref	45.6 (44.8, 46.4)	Ref	56.6 (54.6, 58.5)	Ref	66.0 (64.6, 67.5)	Ref	81.0 (79.5, 82.5)	Ref
Any (n=28701)	71.1 (70.6, 71.6)	1.31 (1.29, 1.33)	64.8 (64.0, 65.5)	1.42 (1.39, 1.45)	72.2 (70.6, 73.7)	1.28 (1.23, 1.33)	78.2 (77.1, 79.3)	1.18 (1.15, 1.22)	86.0 (84.9, 87.1)	1.06 (1.04, 1.09)
Public facility vs. home										
None (n=20997)	47.9 (47.2, 48.6)	Ref	41.0 (40.2, 41.8)	Ref	50.5 (48.3, 52.6)	Ref	59.3 (57.7, 61.0)	Ref	73.2 (71.2, 75.2)	Ref
Any (n=25682)	67.7 (67.1, 68.3)	1.41 (1.39, 1.44)	61.8 (61.0, 62.6)	1.51 (1.47, 1.54)	69.4 (67.7, 71.0)	1.37 (1.31, 1.44)	75.1 (73.9, 76.3)	1.27 (1.23, 1.31)	82.5 (81.1, 83.9)	1.13 (1.09, 1.16)
Private facility vs. home										
None (n=13860)	21.0 (20.4, 21.7)	Ref	12.7 (12.0, 13.4)	Ref	22.0 (19.8, 24.3)	Ref	32.6 (30.6, 34.7)	Ref	60.6 (57.9, 63.2)	Ref
Any (n=11318)	26.7 (25.9, 27.5)	1.27 (1.21, 1.32)	18.3 (17.4, 19.2)	1.44 (1.34, 1.55)	24.8 (22.3, 27.2)	1.12 (0.98, 1.29)	36.4 (34.3, 38.5)	1.11 (1.02, 1.21)	58.8 (56.1, 61.6)	0.97 (0.91, 1.04)

For peer review only

Supplementary table 2: Unadjusted associations between perinatal mortality rate and any third trimester home visits, overall and by education and place of birth

Third trimester home visits	All education groups (n=52588)		None / illiterate (n= 30600)		1-5 standards (n=5689)		6-10 standards (n=9887)		>10 standards (n=6412)	
	Unadjusted PMR (95% CI)	Unadjusted RR (95% CI)	Unadjusted PMR (95% CI)	Unadjusted RR (95% CI)	Unadjusted PMR (95% CI)	Unadjusted RR (95% CI)	Unadjusted PMR (95% CI)	Unadjusted RR (95% CI)	Unadjusted PMR (95% CI)	Unadjusted RR (95% CI)
All births										
Any (n=28686)	40.8 (38.5, 43.1)	Ref	45.4 (42.2, 48.7)	Ref	37.3 (30.8, 43.8)	Ref	35.1 (30.4, 39.9)	Ref	32.7 (27.0, 38.4)	Ref
None (n=23902)	49.5 (46.8, 52.3)	1.21 (1.12, 1.31)	51.3 (47.7, 54.8)	1.13 (1.02, 1.26)	57.1 (47.9, 66.4)	1.53 (1.21, 1.94)	47.1 (40.7, 53.6)	1.34 (1.11, 1.63)	37.0 (29.8, 44.1)	1.13 (87.3, 1.47)
Home delivery										
Any (n=8295)	36.5 (32.5, 40.6)	Ref	39.9 (34.8, 45.1)	Ref	23.1 (13.3, 32.8)	Ref	34.3 (24.2, 44.4)	Ref	28.9 (14.4, 43.0)	Ref
None (n=10935)	40.7 (37.0, 44.4)	1.11 (0.96, 1.29)	42.4 (37.9, 46.8)	1.06 (0.90, 1.25)	36.2 (24.9, 47.5)	1.57 (0.93, 2.65)	39.1 (29.0, 49.3)	1.14 (0.77, 1.69)	27.6 (13.3, 41.8)	0.96 (0.47, 1.97)
Public facility delivery										
Any (n=17426)	38.0 (35.1, 40.8)	Ref	43.5 (39.3, 47.7)	Ref	35.4 (27.4, 43.4)	Ref	31.5 (25.9, 37.1)	Ref	30.0 (23.4, 36.8)	Ref
None (n=10052)	51.3 (47.0, 55.6)	1.35 (1.21, 1.51)	55.2 (49.2, 61.3)	1.27 (1.10, 1.47)	63.6 (48.9, 78.2)	1.80 (1.30, 2.48)	44.8 (35.9, 53.8)	1.42 (1.09, 1.86)	36.0 (26.2, 45.8)	1.20 (0.84, 1.71)
Private facility delivery										
Any (n=3017)	68.6 (59.6, 77.6)	Ref	84.5 (69.1, 99.9)	Ref	93.3 (60.4, 126.3)	Ref	55.9 (39.0, 72.7)	Ref	44.2 (29.4, 58.9)	Ref
None (n=2915)	76.5 (66.9, 86.1)	1.11 (0.92, 1.34)	93.3 (76.5, 110.0)	1.10 (0.85, 1.42)	107.7 (72.5, 143.0)	1.15 (0.71, 1.87)	70.5 (51.3, 89.8)	1.26 (0.84, 1.90)	44.9 (30.3, 59.4)	1.02 (0.64, 1.62)

For peer review only

STROBE 2007 (v4) Statement—Checklist of items that should be included in reports of *cross-sectional studies*

Section/Topic	Item #	Recommendation	Reported on page #
**Title and abstract**	1	(*a*) Indicate the study's design with a commonly used term in the title or the abstract	2
		(*b*) Provide in the abstract an informative and balanced summary of what was done and what was found	2
**Introduction**			
Background/rationale	2	Explain the scientific background and rationale for the investigation being reported	3-4
Objectives	3	State specific objectives, including any prespecified hypotheses	5
**Methods**			
Study design	4	Present key elements of study design early in the paper	5
Setting	5	Describe the setting, locations, and relevant dates, including periods of recruitment, exposure, follow-up, and data collection	5-6
Participants	6	(*a*) Give the eligibility criteria, and the sources and methods of selection of participants	6
Variables	7	Clearly define all outcomes, exposures, predictors, potential confounders, and effect modifiers. Give diagnostic criteria, if applicable	6-8
Data sources/ measurement	8*	For each variable of interest, give sources of data and details of methods of assessment (measurement). Describe comparability of assessment methods if there is more than one group	5-7
Bias	9	Describe any efforts to address potential sources of bias	6, 15
Study size	10	Explain how the study size was arrived at	5-6
Quantitative variables	11	Explain how quantitative variables were handled in the analyses. If applicable, describe which groupings were chosen and why	6-8
Statistical methods	12	(*a*) Describe all statistical methods, including those used to control for confounding	8-9
		(*b*) Describe any methods used to examine subgroups and interactions	8-9
		(*c*) Explain how missing data were addressed	NA
		(*d*) If applicable, describe analytical methods taking account of sampling strategy	NA
		(*e*) Describe any sensitivity analyses	NA
**Results**			

For peer review only

Participants	13*	(a) Report numbers of individuals at each stage of study—eg numbers potentially eligible, examined for eligibility, confirmed eligible, included in the study, completing follow-up, and analysed	6
		(b) Give reasons for non-participation at each stage	6
		(c) Consider use of a flow diagram	NA
Descriptive data	14*	(a) Give characteristics of study participants (eg demographic, clinical, social) and information on exposures and potential confounders	9-10
		(b) Indicate number of participants with missing data for each variable of interest	NA
Outcome data	15*	Report numbers of outcome events or summary measures	9-11, 13
Main results	16	(*a*) Give unadjusted estimates and, if applicable, confounder-adjusted estimates and their precision (eg, 95% confidence interval). Make clear which confounders were adjusted for and why they were included	10-13, Figures 1-2, supplementary Table 1
		(*b*) Report category boundaries when continuous variables were categorized	NA
		(*c*) If relevant, consider translating estimates of relative risk into absolute risk for a meaningful time period	11-14
Other analyses	17	Report other analyses done—eg analyses of subgroups and interactions, and sensitivity analyses	11-14, Figures 1-2
**Discussion**			
Key results	18	Summarise key results with reference to study objectives	15
Limitations	19	Discuss limitations of the study, taking into account sources of potential bias or imprecision. Discuss both direction and magnitude of any potential bias	15
Interpretation	20	Give a cautious overall interpretation of results considering objectives, limitations, multiplicity of analyses, results from similar studies, and other relevant evidence	15-17
Generalisability	21	Discuss the generalisability (external validity) of the study results	15
**Other information**			
Funding	22	Give the source of funding and the role of the funders for the present study and, if applicable, for the original study on which the present article is based	18

*Give information separately for cases and controls in case-control studies and, if applicable, for exposed and unexposed groups in cohort and cross-sectional studies.

Note: An Explanation and Elaboration article discusses each checklist item and gives methodological background and published examples of transparent reporting. The STROBE checklist is best used in conjunction with this article (freely available on the Web sites of PLoS Medicine at http://www.plosmedicine.org/, Annals of Internal Medicine at http://www.annals.org/, and Epidemiology at http://www.epidem.com/). Information on the STROBE Initiative is available at www.strobe-statement.org.

For peer review only

Associations between community health workers' home visits and education-based inequalities in institutional delivery and perinatal mortality in rural Uttar Pradesh, India: a cross-sectional study

Journal:	*BMJ Open*
Manuscript ID	bmjopen-2020-044835.R2
Article Type:	Original research
Date Submitted by the Author:	09-Jun-2021
Complete List of Authors:	Blanchard, Andrea; University of Manitoba Faculty of Health Sciences, Community Health Sciences<br>Colbourn, Timothy; UCL Institute for Global Health,<br>Prost, Audrey; University College London Institute of Child Health,<br>Ramesh, Banadakoppa Manjappa; University of Manitoba Faculty of Health Sciences, Department of Community Health Sciences;<br>Isac, Shajy; India Health Action Trust<br>Anthony, John; University of Manitoba, Department of Community Health Sciences; India Health Action Trust<br>Dehuri, Bidyadhar; India Health Action Trust<br>Houweling, TA; Erasmus Medical Center, Department of Public Health
**Primary Subject Heading**:	Global health
Secondary Subject Heading:	Public health, Epidemiology
Keywords:	EPIDEMIOLOGY, International health services < HEALTH SERVICES ADMINISTRATION & MANAGEMENT, PUBLIC HEALTH, Maternal medicine < OBSTETRICS, PERINATOLOGY

SCHOLARONE™
Manuscripts

I, the Submitting Author has the right to grant and does grant on behalf of all authors of the Work (as defined in the below author licence), an exclusive licence and/or a non-exclusive licence for contributions from authors who are: i) UK Crown employees; ii) where BMJ has agreed a CC-BY licence shall apply, and/or iii) in accordance with the terms applicable for US Federal Government officers or employees acting as part of their official duties; on a worldwide, perpetual, irrevocable, royalty-free basis to BMJ Publishing Group Ltd ("BMJ") its licensees and where the relevant Journal is co-owned by BMJ to the co-owners of the Journal, to publish the Work in this journal and any other BMJ products and to exploit all rights, as set out in our licence.

The Submitting Author accepts and understands that any supply made under these terms is made by BMJ to the Submitting Author unless you are acting as an employee on behalf of your employer or a postgraduate student of an affiliated institution which is paying any applicable article publishing charge ("APC") for Open Access articles. Where the Submitting Author wishes to make the Work available on an Open Access basis (and intends to pay the relevant APC), the terms of reuse of such Open Access shall be governed by a Creative Commons licence – details of these licences and which Creative Commons licence will apply to this Work are set out in our licence referred to above.

Other than as permitted in any relevant BMJ Author's Self Archiving Policies, I confirm this Work has not been accepted for publication elsewhere, is not being considered for publication elsewhere and does not duplicate material already published. I confirm all authors consent to publication of this Work and authorise the granting of this licence.

For peer review only

Associations between community health workers' home visits and education-based inequalities in institutional delivery and perinatal mortality in rural Uttar Pradesh, India: a cross-sectional study

Andrea K. Blanchard[1], Timothy Colbourn[2], Audrey Prost[2], B.M. Ramesh[1], Shajy Isac[1,3], John Anthony[1,3], Bidyadhar Dehury[3], Tanja A.J. Houweling[4]

1. Institute for Global Public Health, University of Manitoba, Winnipeg, Canada
2. Institute for Global Health, University College London, London, United Kingdom
3. India Health Action Trust, New Delhi & Lucknow, India
4. Department of Public Health, Erasmus MC, University Medical Center Rotterdam, The Netherlands

Correspondence to: Andrea Katryn Blanchard, Andrea.Blanchard@umanitoba.ca

Word count: 5075

Number of references: 58

For peer review only - http://bmjopen.bmj.com/site/about/guidelines.xhtml

Abstract

Introduction: India's National Health Mission has trained community health workers called Accredited Social Health Activists (ASHAs) to visit and counsel women before and after birth. Little is known about the extent to which exposure to ASHAs' home visits has reduced perinatal health inequalities as intended. This study aimed to examine whether ASHAs' third trimester home visits may have contributed to equitable improvements in institutional delivery and reductions in perinatal mortality rates between women with varying education levels in Uttar Pradesh state, India.

Methods: Cross-sectional survey data were collected from a representative sample of 52,615 women who gave birth in the preceding two months in rural areas of 25 districts of Uttar Pradesh in 2014-15. We analysed the data using generalised linear modelling to examine the associations between exposure to home visits and education-based inequalities in institutional delivery and perinatal mortality rates.

Results: Third trimester home visits were associated with higher institutional delivery rates, in particular public facility delivery rates (adjusted RR 1.32, 95% CI 1.30-1.34), and to a lesser extent private facility delivery rates (aRR 1.09, 95% CI 1.04-1.13), after adjusting for confounders. Associations were stronger among women with lower education levels. Having no compared to any third trimester home visits was associated with higher perinatal mortality (aRR 1.18, 95% CI: 1.09-1.28). Having any versus no visits was more highly associated with lower perinatal mortality among women with lower education levels than those with the most education, and most notably among public facility births.

Conclusions: The results suggest that ASHAs' home visits in the third trimester contributed to equitable improvements in institutional deliveries and lower perinatal mortality rates, particularly within the public sector. Broader strategies must reinforce the role of ASHAs' home visits in reaching the Sustainable Development Goals of improving maternal and newborn health and leaving no one behind.

Abstract word count: 300

For peer review only

For peer review only - http://bmjopen.bmj.com/site/about/guidelines.xhtml

Article summary

Strengths and limitations

- This study analysed a large population-based survey that allowed the detection of education-based inequalities in institutional delivery and perinatal mortality rates overall and at public and private health facilities separately, as well as analyses of whether these inequalities were lower among women who received community health workers' home visits in the third trimester.
- The survey included women who gave birth within the last 60 days, which likely improved their recall and classification of perinatal compared to late neonatal deaths.
- The cross-sectional survey design precluded conclusions about causality, and the results are not directly generalisable to non-high priority districts in Uttar Pradesh or other states in India.
- The first round of the survey did not include wealth or asset indices, which would have enabled a broader exploration of inequalities in institutional delivery and perinatal mortality rates.
- Survey responses were self-reported, which could lead to recall, social desirability or misclassification biases.

For peer review only

For peer review only - http://bmjopen.bmj.com/site/about/guidelines.xhtml

Background

Since the establishment of the Sustainable Development Goals (SDGs), global health leaders have renewed their pledge to improve maternal and newborn health, with an emphasis on reducing high mortality rates among families who are most socio-economically disadvantaged (1-3). In 2015, 2.7 million infants died in the first month of life, accounting for 45% of child deaths globally. A further 2.6 million were estimated to be stillborn in 2015 (4). Despite considerable progress in the past decades, over one million neonatal deaths and one million stillbirths are estimated to occur every year in South Asia (5). An estimated 28% of neonatal deaths globally occur in India alone (6). The greatest number of deaths happen during the perinatal period, i.e. between 28 completed weeks of gestation and seven days after birth (7, 8). Uttar Pradesh (UP) is India's most populous state and has had the highest perinatal mortality rate (PMR), estimated at 56 per 1000 live births between 2010 to 2015, compared to 36 per 1000 live births nationally (9). Uptake of health services for Reproductive, Maternal, Newborn and Child Health (RMNCH) has historically been low and inequitable between socio-economic groups in many regions of UP. Nearly 68% of women in UP reported delivering in a health facility in 2015/16. This ranged from 56% among women with no education to over 80% for those with 10 or more standards of education overall, while the educational differences were greater at private than public facilities (10, 11).

Community-level interventions are a promising approach to equitably improve maternal and newborn health; most of these rely on Community Health Workers (CHWs) (12-14). CHWs have been defined as people who live and work in their communities, are selected by and accountable to them, and may or may not be part of the health system (15-17). The pursuit of universal health coverage has given CHW programmes renewed importance in public health programmes (3, 18, 19). Global health leaders have endorsed their potential to improve institutional delivery in resource-constrained settings (14, 20, 21). There is also evidence that CHWs can promote safe newborn care practices that prevent perinatal mortality (22-26).

India's National Health Mission (NHM) has recruited and trained a large cadre of CHWs called Accredited Social Health Activists (ASHAs) since 2005. ASHAs are trained to keep a birth register of all pregnant women as well as routinely contact and counsel women and their families on antenatal, delivery, and postnatal care through home visits. During home visits in the third trimester of pregnancy, ASHAs convey the benefits of institutional delivery, review birth plans, and counsel families on essential newborn care practices, all of which are intended to reduce perinatal mortality (27). ASHAs and the women they counsel

For peer review only - http://bmjopen.bmj.com/site/about/guidelines.xhtml

normally receive monetary incentives through the *Janani Suraksha Yojana* scheme for giving birth at a health facility. Services are meant to be free at public health facilities. ASHAs should normally inform women about these services and monetary incentives when preparing a birth plan. They also receive training on how to better reach geographically and socio-economically marginalised families (28). Despite the scale of the ASHA home visiting programme, few studies have examined whether it has improved neonatal health outcomes equitably (as much or more among lower compared to higher socio-economic position groups) (29-31). There is a paucity of research to examine its effects on equity in perinatal mortality overall or comparing between public and private facility births within India's increasingly pluralistic health system.

Our study's overall aim was to examine whether ASHAs' home visits in the third trimester of pregnancy were associated with equitable improvements in institutional delivery and reductions in perinatal mortality rates at public and private facilities between women with lower to higher education levels. The study had three related research questions:

(1) Do women with higher compared to lower education levels report a) higher institutional delivery, and b) lower perinatal mortality rates, overall and by place of birth (home, public, and private facilities)?
(2) Is women's exposure to any third trimester home visits associated with a) higher institutional delivery, and b) lower PMR, overall and by place of birth?
(3) Is exposure to third trimester home visits more strongly associated with a) higher institutional delivery, and b) lower perinatal mortality, for women with lower compared to higher education levels overall and by place of birth?

The conceptual models that guided the study's analyses are shown in Supplementary figures 1 and 2. This study was part of a larger mixed-methods enquiry into the role of ASHAs' home visits in reducing inequities in perinatal health between women of higher and lower socio-economic positions in 25 districts of rural Uttar Pradesh state, India.

Methods

Study design and setting

The Government of UP's branch of the NHM has committed substantial resources towards reducing the burden of maternal and neonatal mortality. In 2013, UP's NHM programme established a partnership with

For peer review only - http://bmjopen.bmj.com/site/about/guidelines.xhtml

the India Health Action Trust and the University of Manitoba in Canada, under the auspices of the Uttar Pradesh Technical Support Unit, funded by the Bill and Melinda Gates Foundation. This partnership was embedded within UP's NHM programmes in its 25 high priority districts (HPDs), covering a population of over 83 million people (32). The government chose the HPDs by ranking districts using a composite health index that combined six health indicators from the state's Annual Health Survey data (33). This study used data from the first round of the Uttar Pradesh Technical Support Unit's community-based, cross-sectional Community Behavioural Tracking Survey (CBTS-1) collected in April 2014 to February 2015, which included 100 blocks (sub-districts) of the 25 HPDs.

Sampling and participants

Sampling was done in two stages, starting with randomly selecting the primary sampling units (PSU), and then inviting all eligible women within them to participate. For the first stage, the survey's PSU was an ASHA area, which covers approximately 1000 people and is the smallest unit of health service delivery. The required sample size in each block to detect a minimum percentage change of 7.5% in the institutional delivery rate within six months (a key indicator expected to change between survey rounds), assuming an estimated non-response rate of 15% and a design effect of 1.5, was 536 women per block or 53,600 across the 100 blocks. This was considered an ideal sample size given the resources for data collection, compared to what was required for a higher or lower minimum detectable percentage change in institutional delivery rate (5% would give n=1209; 7.5%, n=536; 10%, n=300). The average number of PSUs needed to reach a sample size of at least 536 participants per block was estimated to be 110, assuming that there would be five eligible respondents who had completed a pregnancy within the last 60 days per PSU. This assumption was based on the crude birth rate of UP in the most recent available data (29 per 1000 population in the Annual Health Survey in 2011/12). After all PSUs within the 100 blocks of 25 districts were listed, 110 PSUs were chosen in each block (11,000 ASHA areas in total) using systematic random sampling.

For the second stage, every household was enumerated and all eligible women in the household were invited to participate in each of the randomly selected 11,000 PSUs. PSUs were of nearly uniform size and so did not require sample weighting. Eligible participants were women aged 15 to 49 who had completed a pregnancy in the past sixty days. In total, 72,054 women were identified and 57,788 were included in the survey, leading to an 80% response rate. There were no refusals to participate; non-response occurred largely when women were repeatedly unavailable at home when the interviewers visited on different days. In addition, 5,173 participants responded that their pregnancy ended in an abortion, and so were

For peer review only - http://bmjopen.bmj.com/site/about/guidelines.xhtml

not included in this study's sample. We analysed data from 52,615 participants. For analyses on perinatal mortality, the sample used was 52,588: 27 cases (0.05%) could not be included as they had invalid responses. A flow diagram of the sampling process is shown in Supplementary figure 3.

Data collection

Field interviewers' educational background included a minimum of a Bachelor degree in social sciences or related field, and six months or more of related experience. They received ten days of intensive training on data collection methods, including orientation and then hands-on field practice with input from their supervisors. To collect the data, the interviewers first determined the PSU boundary. With a random start, they visited all households and gave each household a number. They used a screening questionnaire in each household to identify women who recently completed a pregnancy, and then described the study to them and asked for their informed consent. Interviews were conducted in Hindi, the local language. Data were collected anonymously using a mobile application, and routinely checked by field supervisors. The survey tool included close-ended questions related to selected background characteristics, pregnancy outcomes, antenatal care, birth preparedness, delivery, postnatal and newborn care, and reproductive health.

Variables

Outcome variables

The outcomes of interest were institutional delivery and perinatal mortality rates. We conducted the analyses with the binary outcome of institutional delivery (public and private) versus home, as well as for institutional delivery at a public facility versus home and a private facility versus home separately. PMR was calculated as the number of stillbirths (occurring between 28 weeks of gestation and delivery) and deaths during the early neonatal period (first seven days of life) out of 1000 births. These were self-reported and not confirmed by verbal autopsies.

Independent variables

Equity in health has been defined as the absence of systematic disparities in health or its social determinants between groups with differing positions in a social hierarchy (34). A number of socio-economic position characteristics are used to describe inequities in health, in terms of wealth or asset indices, education, occupation, caste, ethnicity and religion (34, 35). Wealth or asset-based inequalities would have been valuable to assess, but this indicator was not available in the first round of the CBTS. We

For peer review only - http://bmjopen.bmj.com/site/about/guidelines.xhtml

examined caste group differences, but they were more consistent for institutional delivery rates than PMR. We focused on education as the main indicator of socio-economic position in this study as there were consistent inequalities in both outcomes. Higher maternal education has been associated with child survival and utilisation of maternal health services in previous research (36). Education levels represent socio-economic opportunities that are achieved early in life and remain fairly stable over the lifetime, being relatively uninfluenced by later health status. It is also an ordinal variable and enables the examination of gradients in health outcomes (37). We categorised education levels into four groups from least to most years of study (standards) based on common Indian school system divisions: none or illiterate, 1 to 5 (lower primary school), 6 to 10 (higher primary to lower secondary), or more than 10 standards (upper secondary and college) (37).

Receipt of ASHA home visits in the third trimester of pregnancy was the main intervention exposure variable. We considered exposure to home visits earlier in pregnancy, but only 9.8% of women received a home visit in the first two trimesters of pregnancy and not in the third trimester in the CBTS-1. This suggested that the binary variable of any third trimester home visit was a good single indicator of ASHA home visit coverage. Third trimester visits are also meant to focus substantially on encouraging and preparing women for institutional delivery.

Covariate and confounding variables

Women's age, caste, religion and parity are known to be associated with place of birth, as well as their risk of perinatal loss; all but parity were available in the CBTS-1 data (34-36, 38-40). Having any antenatal care visits can increase the likelihood of birth preparedness and relatedly, having an institutional delivery as well (36, 38). Pregnancy, intrapartum and newborn complications have also been found to lead to higher institutional delivery rates, and all can affect mortality rates (38). Among these, the available covariates in the dataset that were found to be significant at $p<0.05$ in bivariable models were retained in multivariable models.

Maternal age and caste (ordinal categorical variables) were adjusted for as confounders in all multivariable models. Religion and any antenatal check-ups (binary variables) were included for institutional delivery, but not PMR, because they were significantly associated with the former but not the latter in the bivariable models. We also adjusted for the binary variables of experiencing any pregnancy complications in analyses with both outcomes, as well as any intrapartum complications, and then place of delivery for perinatal mortality. Pregnancy complications were defined as experiencing any

For peer review only - http://bmjopen.bmj.com/site/about/guidelines.xhtml

of the following: excessive bleeding before delivery, convulsions, high blood pressure, or sepsis/fever. Intrapartum complications included any of the following: premature labour, preterm/premature rupture of the membrane, prolonged labour of more than 12 hours, obstructed labour, breach/malpresentation, or excessive bleeding immediately after delivery.

Statistical analyses

Data management and analyses were conducted in STATA 13.0. First, we described the independent variables, outcomes and covariates, using tabulations to produce proportions and related confidence intervals for all variables; for perinatal mortality rates, we summarised the mean number of women reporting a perinatal death, and calculated the rate of perinatal deaths per 1000 births.

Our first question was whether women of higher compared to lower education levels reported a) higher institutional delivery, and b) lower perinatal mortality rates, overall and by place of birth. We used cross-tabulations to describe the proportion of women reporting an institutional delivery, in each education group overall and at each place of delivery. Similarly, we calculated the PMR among women in each education group, overall and by place of birth.

To answer our second question, we aimed to assess if women's exposure to any third trimester home visits was associated with a) higher institutional delivery and b) lower PMR, overall and by place of birth. We used bivariable and multivariable generalised linear models to assess whether a) institutional delivery, and then b) PMR, were independently associated with having any third trimester home visits, before and after adjusting for the covariate and confounding factors. Each of the models were run first for all births, and then stratified to compare home, public, and private facility births.

For the final question, we examined whether exposure to third trimester home visits was more strongly associated with a) higher institutional delivery, and b) lower perinatal mortality for women with lower compared to higher education levels, overall and by place of birth. We descriptively assessed whether there was higher institutional delivery and lower PMR, among women with lower compared to higher education if they had any compared to no third trimester home visits, by place of delivery. We then used stratified unadjusted and adjusted generalised linear models to examine whether a) higher institutional delivery, and b) lower PMR outcomes, were more strongly associated with women's exposure to third trimester home visits for women with lower compared to higher education groups, overall and by place of delivery. Interaction terms between exposure to home visits and the outcomes were also initially examined. These were significant for institutional delivery in the adjusted models, but not for PMR; the

For peer review only - http://bmjopen.bmj.com/site/about/guidelines.xhtml

latter showed uneven gradients by education groups that were likely obscured in the interaction term. We therefore focused on the stratified results as they were more interpretable and transparent about where uneven patterns of inequalities and associations existed. Sensitivity analyses were not applicable for the present analysis.

In the analyses to address analytical questions 2 and 3, multivariable models with institutional delivery as outcome were adjusted for maternal age, caste, religion, any pregnancy complications and any antenatal check-ups. The multivariable models with PMR as the outcome were all adjusted for maternal age, caste, any pregnancy complications and any intrapartum complications. After adjusting for these covariates, the models with PMR were also adjusted for place of birth to assess how this affected the relationship between exposure to home visits and PMR. Ordinal variables were indicated as such in the STATA commands to obtain distinct estimates for each category relative to a reference category.

The generalised linear models used the binomial family distribution and a log link function to obtain risk ratios (RR). Some adjusted models with binomial distribution failed to converge for the institutional delivery outcome. Others have found that this can occur when including variables with few data points in some categories, and addressed this by using a Poisson distribution with robust standard errors to produce the RR (41). Using robust Poisson models with a log link function produced estimates that were almost identical to our analyses using a log binomial distribution in models that converged. Therefore, the RR were produced from robust Poisson and log binomial models for institutional delivery and PMR respectively. For both outcomes, we also present the unadjusted and adjusted percentages of institutional delivery and perinatal mortality rates per 1000 live births, alongside the unadjusted and adjusted RR estimates in the results. To aid interpretation, results for all three questions are presented first for institutional delivery, and then for perinatal mortality.

All data collection protocols and analysis plans were approved by the Institutional Review Board of Sigma Research and Consulting in New Delhi [#10040/IRB/D/16-17], and the University College London Research Ethics Committee [#9909/001].

Patient and public involvement statement

No patients or members of the public were involved in this study.

For peer review only - http://bmjopen.bmj.com/site/about/guidelines.xhtml

Results

Table 1 presents participants' characteristics. Eighty-one percent of women were 15 to 29 years old (Table 1). The majority of women belonged to Other Backward Class groups (47%), followed by Scheduled Caste (23%) and General Caste (12%) groups. Also, 58% of women were illiterate or had no formal education. Over half of the participants (55%) received any home visits during the third trimester, and 48% had gone for any ANC check-up. About 18% of women reported having any pregnancy complications, while 39% reported intrapartum complications.

Table 1: Descriptive socio-demographic and health-related characteristics of the sample

Variable	Total, n (%)	Any third trimester home visit (%)	Institutional delivery			Perinatal mortality rate (per 1000 births)[a.]
			Total (%)	Public facility (%)	Private facility (%)	
**Overall**	52615 (100)	55	63	52	11	45
**Age**						
15-24 years	21079 (40)	55	70	56	14	49
25-29 years	21400 (41)	55	62	51	11	39
30+ years	10136 (19)	53	55	47	8	49
**Religion**						
Hindu	42720 (81)	55	64	53	11	45
Muslim/Other	9895 (19)	51	59	46	13	42
**Caste**						
Scheduled Tribe	2216 (4)	40	55	49	6	43
Scheduled Caste	12334 (23)	55	62	54	8	44
Other Backward Class	24579 (47)	55	63	52	11	45
General Caste	6323 (12)	53	72	53	19	48
Don't know	7163 (14)	57	61	49	12	44
**Education**						
None/ illiterate	30614 (58)	52	56	48	8	48
1-5 standards	5693 (11)	57	66	55	11	46
6-10 standards	9895 (19)	58	73	59	14	40
>10 standards	6413 (12)	58	84	60	24	35
**Any pregnancy complications**						
No	43102 (82)	55	63	52	11	38
Yes	9513 (18)	51	67	53	14	76

For peer review only - http://bmjopen.bmj.com/site/about/guidelines.xhtml

Variable	Total, n (%)	Any third trimester home visit (%)	Institutional delivery			Perinatal mortality rate (per 1000 births)[a]
			Total (%)	Public facility (%)	Private facility (%)	
**Any intrapartum complications**						
No	32000 (61)	55	60	51	9	32
Yes	20615 (39)	53	69	54	15	65
**Any ANC check-ups**						
No	27179 (52)	44	54	45	9	45
Yes	25436 (48)	66	73	59	14	45
**Any third trimester home visits**						
No	23914 (45)	NA	54	42	12	50
Yes	28701 (55)	NA	71	61	10	41

[a.] There were 27 missing values (0.05% of 52,615 responses) for the perinatal mortality variable, due to invalid responses.

Institutional delivery

Almost two-thirds (63%) of women gave birth at a health facility (52% in a public facility, 11% in a private facility). To address research question 1 a), institutional delivery was higher for women with the highest compared to lowest education levels (84% versus 56% respectively). Educational inequalities were apparent for both public and private facilities (Table 1).

Relating to the second question, a greater proportion of women who had any versus no third trimester home visits reported giving birth in a public facility (61% vs. 42% respectively). An equal proportion had private facility births without or with any third trimester home visits (12% vs. 10% respectively) (Table 1). Women who had received any third trimester home visit compared to none reported higher institutional delivery overall (71% vs. 54%) (unadjusted RR 1.31 [95%CI: 1.29, 1.33]) (supplementary table 1). This association remained significant after adjusting for confounders (adjusted RR 1.23 [95%CI: 1.22, 1.25]). The association between having any third trimester home visit and an institutional birth was stronger for births at a public facility (aRR 1.32 [95% CI: 1.30-1.34]) than those at a private facility (aRR 1.08 [95% CI: 1.04-1.13]) compared to home (Table 2).

Turning to research question 3 a), Figure 1 indicates that a higher proportion of women gave birth at public facilities versus home when they had any compared to no third trimester visits from an ASHA, and this was most noticeable among the least educated groups.

[Figure 1]

For peer review only - http://bmjopen.bmj.com/site/about/guidelines.xhtml

Similarly, the adjusted associations shown in Table 2 between having an institutional birth (at both public and private facilities) and any third trimester home visit was stronger with each step down the educational gradient. Stratifying by facility type, this was more noticeable among public than private facility births compared to home.

For peer review only

For peer review only - http://bmjopen.bmj.com/site/about/guidelines.xhtml

Table 2: Associations between institutional delivery and any third trimester home visits, overall and by education level

Third trimester home visits	All education groups (n=52615)		None / illiterate (n= 30614)		1-5 standards (n=5693)		6-10 standards (n=9895)		>10 standards (n=6413)	
	Adjusted % (95% CI)	Adjusted RR (95% CI)	Adjusted % (95% CI)	Adjusted RR (95% CI)	Adjusted % (95% CI)	Adjusted RR (95% CI)	Adjusted % (95% CI)	Adjusted RR (95% CI)	Adjusted % (95% CI)	Adjusted RR (95% CI)
Institution (public and private) vs. home										
None (n=23914)	54.2 (53.6, 54.9)	Ref	47.2 (46.4, 48.0)	Ref	57.5 (55.6, 59.5)	Ref	67.2 (65.7, 68.6)	Ref	81.6 (80.1, 83.1)	Ref
Any (n=28701)	71.1 (70.6, 71.6)	1.23 (1.22, 1.25)	62.9 (62.2, 63.6)	1.33 (1.30, 1.36)	71.3 (69.7, 72.8)	1.24 (1.19, 1.29)	77.3 (76.2, 78.4)	1.15 (1.12, 1.18)	85.6 (84.4, 86.7)	1.05 (1.03, 1.07)
Public facility vs. home										
None (n=20997)	47.9 (47.2, 48.9)	Ref	42.4 (41.5, 43.2)	Ref	51.5 (49.4, 53.7)	Ref	60.6 (58.9, 72.9)	Ref	74.1 (72.1, 76.1)	Ref
Any (n=25682)	67.7 (67.1, 68.3)	1.32 (1.30, 1.34)	59.9 (59.2, 60.7)	1.41 (1.38, 1.45)	68.4 (66.7, 70.1)	1.33 (1.26, 1.39)	74.1 (72.9, 75.3)	1.22 (1.18, 1.26)	81.9 (80.5, 83.3)	1.11 (1.07, 1.14)
Private facility vs. home										
None (n=13860)	22.6 (22.0, 23.3)	Ref	13.7 (13.0, 14.4)	Ref	22.8 (20.6, 25.0)	Ref	33.8 (31.8, 35.9)	Ref	61.4 (58.9, 64.0)	Ref
Any (n=11318)	24.6 (23.9, 25.3)	1.09 (1.04, 1.13)	16.6 (15.8, 17.5)	1.21 (1.12, 1.31)	23.9 (21.6, 26.2)	1.05 (0.91, 1.20)	35.1 (33.1, 37.1)	1.04 (0.95, 1.13)	58.0 (55.3, 60.6)	0.94 (0.87, 1.00)

For peer review only - http://bmjopen.bmj.com/site/about/guidelines.xhtml

Perinatal mortality rate

The overall perinatal mortality rate in the CBTS-1 sample was 45 per 1000 births (Table 1). The greatest number of early neonatal deaths occurred on the day of birth, and steadily decreased in number on subsequent days (supplementary Figure 4). The PMR was 39 per 1000 births among women giving birth at home, 43 per 1000 births at public facilities, and 73 per 1000 births at private facilities overall. For question 1 b), the PMR reduced with increasing educational attainment, from 48 to 35 per 1000 births among women with no education compared to more than ten standards respectively. Education inequalities in PMR were evident among home, public, and private facility births.

On question 2 b), the PMR was higher among women without compared to with any third trimester home visit, at 50 compared to 41 per 1000 births respectively (unadjusted RR 1.21 [95% CI: 1.12, 1.31]) (supplementary Table 2). The association remained after adjustment for socio-demographic characteristics, any pregnancy and intrapartum complications (aRR 1.17 [95% CI: 1.98, 1.27]), and after additional adjustment for place of birth (aRR 1.18 [95% CI: 1.09, 1.28]) (Table 3). Comparing between places of birth, the inverse association between any third trimester home visits and PMR was strong for births at public facilities (aRR 1.31 [95% CI: 1.17, 1.47]). There was no association for births at private facilities (aRR 1.12 [95% CI: 0.93, 1.34]) or for home births (aRR 1.04 [95% CI: 0.91, 1.21]).

In relation to question 3 b), Figure 2 descriptively suggests that for women who had any versus no third trimester home visits, educational differences in PMR were smaller among public facility births and to a lesser extent private facility births. The adjusted models showed that the association between having no third trimester home visits and higher PMR was stronger for those with one to five standards of education (aRR 1.53 [95% CI: 1.20, 1.94]), and to a lesser extent 5 to 10 standards (aRR 1.27 [95% CI: 1.05, 1.54]). The association appeared somewhat weaker for women with no education (aRR 1.13 [95% CI: 1.02, 1.25]), and absent among women who had an education over ten standards (Table 3).

[Figure 2]

When stratifying the adjusted models by place of birth, PMR was significantly higher among those without third trimester home visits compared to those with any visits for public facility births for all except the most educated group (Table 3). However, differences in PMR between women with and without third trimester home visits were not significant for those who delivered at home or in private facilities, across all education groups. Confidence intervals were wide in some of the comparisons based on lower numbers of perinatal deaths.

For peer review only - http://bmjopen.bmj.com/site/about/guidelines.xhtml

Table 3: Adjusted associations between perinatal mortality rate and any third trimester home visits, overall and by education and place of birth

Third trimester home visits	All education groups (n=52588)		None / illiterate (n= 30600)		1-5 standards (n=5689)		6-10 standards (n=9887)		>10 standards (n=6412)	
	Adjusted PMR (95% CI)	Adjusted RR (95% CI)	Adjusted PMR (95% CI)	Adjusted RR (95% CI)	Adjusted PMR (95% CI)	Adjusted RR (95% CI)	Adjusted PMR (95% CI)	Adjusted RR (95% CI)	Adjusted PMR (95% CI)	Adjusted RR (95% CI)
All births, not adjusting for place of birth										
Any (n=28686)	41.5 (39.2, 43.8)	Ref	45.9 (42.7, 49.2)	Ref	37.8 (21.2, 44.3)	Ref	35.6, 30.8, 40.4)	Ref	33.1 (27.3, 38.9)	Ref
None (n=23902)	48.6 (45.9, 51.3)	1.17 (1.08, 1.27)	50.7 (47.2, 54.2)	1.10 (1.00, 1.22)	56.1 (47.0, 65.1)	1.48 (1.17, 1.88)	46.3 (40.0, 52.6)	1.30 (1.07, 1.58)	36.3 (29.3, 43.3)	1.10 (0.84, 1.42)
All births, adjusting for place of birth										
Any (n=28686)	41.3 (39.0, 43.6)	Ref	45.5 (42.2, 48.7)	Ref	37.3 (30.8, 43.8)	Ref	36.0 (31.1, 40.9)	Ref	33.3 (27.5, 39.2)	Ref
None (n=23902)	48.9 (46.1, 51.6)	1.18 (1.09, 1.28)	51.2 (47.7, 54.8)	1.13 (1.02, 1.25)	56.9 (47.7, 66.2)	1.53 (1.20, 1.94)	45.7 (39.4, 52.0)	1.27 (1.05, 1.54)	35.9 (28.9, 42.9)	1.07 (0.83, 1.40)
Home delivery										
Any (n=8295)	37.8 (33.7, 42.0)	Ref	41.1 (35.8, 46.4)	Ref	23.7 (13.7, 33.7)	Ref	35.5 (25.1, 45.9)	Ref	30.0 (14.9, 45.1)	Ref
None (n=10935)	39.7 (36.1, 43.3)	1.04 (0.91, 1.21)	41.6 (37.2, 45.9)	1.01 (0.86, 1.19)	35.4 (24.4, 46.5)	1.50 (0.88, 2.53)	38.0 (28.2, 47.8)	1.07 (0.72, 1.58)	26.3 (12.6, 40.0)	0.88 (0.42, 1.82)
Public facility delivery										
Any (n=17426)	38.4 (35.5, 41.3)	Ref	43.8 (39.6, 48.0)	Ref	35.6 (27.6, 43.6)	Ref	31.8 (26.2, 37.4)	Ref	30.4 (23.6, 37.2)	Ref
None (n=10052)	50.4 (46.2, 54.6)	1.31 (1.17, 1.47)	54.5 (48.5, 60.4)	1.24 (1.08, 1.44)	62.7 (48.3, 43.6)	1.76 (1.28, 2.43)	44.0 (35.3 (52.8)	1.38 (1.06, 1.81)	35.3 (25.7, 44.9)	1.16 (0.82, 1.65)
Private facility delivery										
Any (n=3017)	68.5 (59.6, 77.4)	Ref	84.8 (69.6, 100.0)	Ref	96.0 (62.4, 129.6)	Ref	56.9 (40.0, 73.8)	Ref	43.5 (29.0, 58.0)	Ref
None (n=2915)	76.5 (67.0, 86.1)	1.12 (0.93, 1.34)	93.1 (76.6, 109.5)	1.10 (0.85, 1.41)	104.5 (70.8, 138.8)	1.09 (0.68, 1.76)	69.4 (50.7, 88.0)	1.22 (0.82, 1.82)	45.5 (30.8, 60.2)	1.05 (0.66, 1.66)

For peer review only - http://bmjopen.bmj.com/site/about/guidelines.xhtml

Discussion

This study sought to examine the associations between women's exposure to ASHAs' third trimester home visits and education-based inequalities in institutional delivery and perinatal mortality rates in 25 districts of Uttar Pradesh, India. The results indicate that there were education-based inequalities in institutional delivery and PMR, overall and at each delivery place. Women's receipt of any ASHA home visits during women's third trimester of pregnancy was associated with giving birth in public facilities, and this association was increasingly strong for decreasing education levels. Women's receipt of any ASHA home visit in the third trimester was also significantly associated with lower PMR, and most strongly for women giving birth in public facilities among all but the most educated.

There were some limitations to the analyses. CBTS-1 survey responses were self-reported, and therefore prone to recall, misclassification and social desirability biases. The survey included women who delivered in the past 60 days to improve recall. However, the response rate was reduced somewhat because some women were not yet home from the facility or went to their maternal home in this period. The measure of an 'early' neonatal death could have been over- or under-reported, though combining stillbirths and early neonatal deaths to measure PMR can avoid the common misclassification of early neonatal deaths as stillbirths. We did not observe heaping of reported neonatal deaths within the first six days after birth, and the proportion of neonatal deaths at various times after delivery was similar across education groups. The CBTS-1 offered a sufficiently large population-based sample to measure PMR, and to stratify the analyses by education and place of delivery. However, its purpose as a programme monitoring methodology resulted in lack of data on some relevant factors or confounders, such as wealth, parity, mode of previous deliveries, newborn's sex, or having a singleton or twin birth that might have attenuated the strength of observed associations if included (42). The cross-sectional nature of the analysis precluded conclusions about causality. Moreover, we assumed that the associations between exposure and outcome variables could involve reverse or multidirectional, rather than linear, pathways of causation (43). For example, ASHAs may have more often chosen to visit women who preferred public facility births as a way to receive their incentives, while women with lower education may more often opt for the less expensive public services. The results may not be directly generalisable to non-HPD districts of UP, where institutional delivery was likely higher and PMR lower, and possibly less unequal.

Our results suggested that having any third trimester home visits from an ASHA was associated with a greater chance of women having an institutional delivery, and particularly at public facilities. Further,

For peer review only - http://bmjopen.bmj.com/site/about/guidelines.xhtml

having any third trimester home visits was more greatly associated with having an institutional delivery for mothers with lower education levels. Other studies have reported positive associations between exposure to community-based interventions and lower socio-economic inequalities in institutional delivery and skilled birth attendance in UP. Another cross-sectional study found that having any ASHA home visit was associated with higher institutional delivery among poorer and illiterate women, but not among richer or more educated women (30). A quasi-experimental study of an intervention involving NGO technical support to the ASHA programme in two districts of UP found that skilled birth attendance became more equitable between wealth groups in the intervention district but not the comparison district (29). The role of ASHAs' home visits in increasing the proportion of institutional deliveries, and particularly at public facilities, among women with less education and to a lesser extent more education may be due to their role in promoting the free public health services and incentives within the National Health Mission. Studies have found that ASHAs have generally had a strong role in encouraging women to attend public antenatal and delivery care, in part by promoting the *Janani Suraksha Yojana* incentives, and that this has occurred alongside shifting social norms in favour of facility-based maternity care in India (44-47). Our findings seem to support evidence indicating that having at least one home visit in the third trimester may contribute to improving institutional delivery not only overall but equitably, by facilitating access to the more affordable care in the public health system in UP. It would be valuable for future research to examine whether having multiple home visits leads to even better outcomes, and to compare this for women of lower and higher age and birth order who tend to be at greater risk of perinatal loss.

This quantitative study indicated that ASHAs' home visits played the strongest role in supporting women of lower education groups to give birth at public facilities. Yet women from the lower, and even higher, education groups who delivered at private facilities still reported high perinatal mortality, though there were fewer cases leading to wider confidence intervals. Relatedly, the qualitative component of our wider mixed-methods study suggested that ASHAs' home visits encouraged more poorer and less educated women to deliver at public community health centres (48). Those who had to be referred to higher-level, often private facilities, to receive emergency obstetric care faced delays and economic barriers that increased their risk of perinatal loss and thereby perpetuated inequities. It is therefore important for programmes to consider that improvements in institutional delivery have not always translated into reductions in mortality among mothers and their babies where there has been lower quality of services and poor referral mechanisms, particularly at primary health care facilities or unregulated private services where people of lower socio-economic positions often attend first (49, 50). More research is needed to

For peer review only - http://bmjopen.bmj.com/site/about/guidelines.xhtml

understand the processes by which inequitably high perinatal mortality rates continue for women of lower socio-economic positions, especially those who face pregnancy and intrapartum complications in different contexts.

In 2018, the Government of India launched the *Ayushman Bharat* Programme to enhance the availability of free integrated primary health care services through village-level Health and Wellness Centers (51). The programme aims to attach five ASHAs to each centre, where they will work with new mid-level health providers. ASHAs are to continue home visits to promote utilisation and safe health behaviours for RMNCH, among a range of other health services (51). A systematic review of previous studies in India and elsewhere found that lower neonatal mortality was associated with CHW home visits, particularly when they included both preventive (e.g. counselling on care seeking or behaviours) and curative roles (e.g. provision of injectable antibiotics) (52). Another showed that the effects of CHW home visits on care-seeking for institutional delivery can be equal or greater for lower compared to higher wealth or education groups (53). It will be crucial to support ASHAs to continue providing counselling on institutional delivery, essential newborn care, as well as timely identification, treatment or referrals for women that also face greater vulnerability due to socio-economic disadvantage (3, 51, 54, 55).

Building on the NHM, the *Ayushman Bharat* policies are also explicitly based on the premise that the health system functions effectively (51). The programme includes public as well as empanelled private first referral units to be covered by the insurance scheme called *Pradhan Mantri Jan Arogya Yojana*, which should remove financial barriers for women of lower socio-economic groups facing birth complications (56). As more women and their families are giving birth at public and private health facilities, it is particularly important to consider the pluralistic health system in India holistically when addressing maternal and perinatal health inequities (50, 56-58). This would necessitate targeting the causes of differential gaps in accessibility, quality and integration of the private and public health sectors, and between community and facility-based services, so as to strategically address persistent inequalities in perinatal mortality.

Conclusions

This study's findings suggested that ASHAs' contacts and counselling for pregnant women through home visits in the third trimester may have influenced them to give birth at a public facility, and particularly those with lower compared to higher education levels. Further, less educated women who had any compared to no home visits that delivered at a public facility appeared to have the lowest rates of

For peer review only - http://bmjopen.bmj.com/site/about/guidelines.xhtml

perinatal mortality. Future research, policies, and programmes should seek to further understand the processes by which community health workers' home visits can contribute to improving equity in perinatal health outcomes, and how more integrated community and health system efforts can strategically support these efforts.

Funding

This work was supported by the Bill & Melinda Gates Foundation [OPP1161429]. AKB's time was funded by University College London's Overseas and Graduate Research Scholarships. TAJH's time was funded by a Research Excellence Initiative grant from Erasmus University Rotterdam, The Netherlands. The funders had no role in the design of the study, data collection and analysis, planning or writing of the manuscript.

Contributor statement

AKB planned and conducted the analyses, and wrote the draft manuscript. TAJH, TC and AP provided guidance on the design of the study and support throughout the analyses. BMR, SI, JA, and BD designed the CBTS methodology, managed the data collection and quality assurance, and gave technical input on the analyses. All authors read and gave input on the manuscript.

Competing interests

The authors declare no competing interests.

Data sharing statement

The data are hosted on the University of Manitoba server, and a de-identified data cut along with the questionnaires and data dictionary may be made available upon reasonable request (contact e-mail: Ramesh.BanadakoppaManjappa@umanitoba.ca).

Figures

Figure 1: Proportion of women in each education group giving birth at home, public facilities, or private facilities, by exposure to any third trimester home visits

Figure 2: Perinatal mortality rate among women in each education group, by exposure to any third trimester home visits and place of birth

For peer review only - http://bmjopen.bmj.com/site/about/guidelines.xhtml

Bibliography

1. Houweling TAJ, Morrison J, Azad K, Manandhar DS, Alcock G, Shende S, Tripathy P, Costello A. How to reach every newborn: Three key messages. The Lancet Global Health. 2014;2(8):e436-e7. Available from: doi:10.1016/S2214-109X(14)70271-2.

2. Requejo JH, Bryce J, Barros AJ, Berman P, Bhutta Z, et al. Countdown to 2015 and beyond: fulfilling the health agenda for women and children. Lancet. 2015;385(9966):466-76. Available from: doi:10.1016/S0140-6736(14)60925-9.

3. Boerma T, Requejo J, Victora CG, Amouzou A, George A, Agyepong I, et al. Countdown to 2030: tracking progress towards universal coverage for reproductive, maternal, newborn, and child health. The Lancet. 2018;391(10129):1538-48. Available from: doi:10.1016/S0140-6736(18)30104-1.

4. You D, Hug, L, Ejdemyr, S, Beise, J. Levels and Trends in Child Mortality Report 2015. Geneva: Inter-Agency Group for Child Mortality Estimation, 2015.

5. Akseer N, Kamali M, Arifeen SE, Malik A, Bhatti Z, Thacker N, et al. Progress in maternal and child health: how has South Asia fared? Bmj. 2017;357:j1608. Available from: doi:10.1136/bmj.j1608.

6. Black RE, R. Laxminarayan, M. Temmerman, and N. Walker. (eds.) Reproductive, Maternal, Newborn, and Child Health. Disease Control Priorities. 3 ed. Washington, DC: World Bank; 2016.

7. United Nations Children Fund. Neonatal Mortality. Geneva: UNICEF; 2018 [cited 2018 March 12]. Available from: https://data.unicef.org/topic/child-survival/neonatal-mortality/

8. World Health Organization. Maternal and perinatal health. Geneva: World Health Organization; 2018 [cited 2018 March 12]. Available from: http://www.who.int/maternal_child_adolescent/topics/maternal/maternal_perinatal/en/.

9. International Institute for Population Sciences (IIPS) and ICF. National Family Health Survey (NFHS-4), 2015-16: India. Mumbai: Ministry of Health and Family Welfare, 2017.

10. Dettrick Z, Jimenez-Soto E, Hodge A. Socioeconomic and geographical disparities in under-five and neonatal mortality in Uttar Pradesh, India. Maternal and Child Health Journal. 2014;18(4):960-9. Available from: doi:10.1007/s10995-013-1324-8.

11. International Institute for Population Sciences (IIPS) and ICF. National Family Health Survey (NFHS-4), India, 2015-16: Uttar Pradesh. Mumbai: 2017.

12. Bhutta ZA, Das JK, Rizvi A, Gaffey MF, Walker N, Horton S, et al. Evidence-based interventions for improvement of maternal and child nutrition: what can be done and at what cost? Lancet. 2013;382(9890):452-77. Available from: doi:10.1016/s0140-6736(13)60996-4.

13. Ekman B, Pathmanathan I, Liljestrand J. Integrating health interventions for women, newborn babies, and children: a framework for action. Lancet. 2008;372(9642):990-1000. Available from: doi:10.1016/S0140-6736(08)61408-7.

For peer review only

For peer review only - http://bmjopen.bmj.com/site/about/guidelines.xhtml

14. Bhutta ZA, Black RE. Global maternal, newborn, and child health - So near and yet so far. New England Journal of Medicine. 2013;369(23):2226-35. Available from: doi:10.1056/NEJMra1111853.

15. Olaniran A, Smith H, Unkels R, Bar-Zeev S, van den Broek N. Who is a community health worker? – a systematic review of definitions. Glob Health Action. 2017;10(1):1-13. Available from: doi:10.1080/16549716.2017.1272223.

16. World Health Organization. Community health workers: a strategy to ensure access to primary health care services. Cairo: Regional Office for the Eastern Mediterranean, 2016.

17. Tulenko K, Mogedal S, Afzal MM, Frymus D, Oshin A, Pate M, et al. Community health workers for universal health-care coverage: from fragmentation to synergy. Bull World Health Organ. 2013;91(11):847-52. Available from: doi:10.2471/BLT.13.118745.

18. Perry H. A Brief History of Community Health Worker Programs. In: Perry H, Crigler L. (eds.) Developing and Strengthening Community Health Worker Programs at Scale - A Reference Guide and Case Studies for Program Managers and Policymakers. Washington: United States Agency for International Development; 2014. p. 1-14.

19. The Lancet. The Astana Declaration: the future of primary health care? The Lancet. 2018;392(10156):1369. Available from: doi:10.1016/S0140-6736(18)32478-4.

20. Victora CG, Wagstaff A, Schellenberg JA, Gwatkin D, Claeson M, Habicht J-P. Applying an equity lens to child health and mortality: more of the same is not enough. The Lancet. 2003;362(9379):233-41. Available from: doi:https://doi.org/10.1016/S0140-6736(03)13917-7.

21. Bhutta ZA, Reddy KS. Achieving equity in global health: So near and yet so far. JAMA - Journal of the American Medical Association. 2012;307(19):2035-6. Available from: doi:10.1001/jama.2012.4659.

22. Gilmore B, McAuliffe E. Effectiveness of community health workers delivering preventive interventions for maternal and child health in low- and middle-income countries: a systematic review. BMC public health. 2013;13(847):1-14.

23. Gogia S, Sachdev HS. Home visits by community health workers to prevent neonatal deaths in developing countries: a systematic review. Bulletin of the World Health Organization. 2010;88(9):658-66B. Available from: doi:http://dx.doi.org/10.2471/BLT.09.069369.

24. Prost A, Pagel C, Costello A. Community interventions to reduce maternal and child mortality in lowincome countries. Maternal and Infant Deaths: Chasing Millennium Development Goals 4 and 52010. p. 205-16.

25. Haines A, Sanders D, Lehmann U, Rowe AK, Lawn JE, Jan S, et al. Achieving child survival goals: potential contribution of community health workers. Lancet. 2007;369(9579):2121-31. Available from: doi:10.1016/s0140-6736(07)60325-0.

26. Lassi ZS, Bhutta ZA. Community-based intervention packages for reducing maternal and neonatal morbidity and mortality and improving neonatal outcomes. Cochrane Database Syst Rev. 2015(3):1-78. Available from: doi:10.1002/14651858.CD007754.pub3.

For peer review only - http://bmjopen.bmj.com/site/about/guidelines.xhtml

27. Ministry of Health and Family Welfare. Guidelines for Community Processes - National Rural Health Mission. New Delhi: Government of India, 2014.

28. Ministry of Health and Family Welfare. ASHA Training Modules - National Health Mission. Delhi: Government of India; 2014 [cited 2017 March 28]. Available from: http://nrhm.gov.in/communitisation/asha/resources/asha-training-modules.html.

29. Baqui AH, Rosecrans AM, Williams EK, Agrawal PK, Ahmed S, Darmstadt GL, et al. NGO facilitation of a government community-based maternal and neonatal health programme in rural India: improvements in equity. Health Policy Plan. 2008;23(4):234-43. Available from: doi:http://dx.doi.org/10.1093/heapol/czn012.

30. Seth A, Tomar S, Singh K, Chandurkar D, Chakraverty A, Dey A, et al. Differential effects of community health worker visits across social and economic groups in Uttar Pradesh, India: a link between social inequities and health disparities. International Journal for Equity in Health. 2017;16(46):1-9. Available from: doi:10.1186/s12939-017-0538-6.

31. Rajbhandari S, Hodgins S, Sanghvi H, McPherson R, Pradhan YV, Baqui AH, Misoprostol Study Group. Expanding uterotonic protection following childbirth through community-based distribution of misoprostol: operations research study in Nepal. Int J Gynaecol Obstet. 2010;108(3):282-8. Available from: doi:http://dx.doi.org/10.1016/j.ijgo.2009.11.006.

32. Bill and Melinda Gates Foundation. Maternal, Newborn and Child Health. Seattle: Bill and Melinda Gates Foundation; 2014 [cited 2017 December 12]. Available from: http://www.gatesfoundation.org/What-We-Do/Global-Development/Maternal-Newborn-and-Child-Health.

33. Ministry of Health and Family Welfare. Guidance note for implementation of RMNCH+A interventions in High Priority Districts. New Delhi: Government of India, 2013.

34. Braveman P, Gruskin S. Defining equity in health. J Epidemiol Commun Health. 2003;57:254-8. Available from: doi:10.1136/jech.57.4.254.

35. Marmot M. Closing the health gap in a generation: the work of the Commission on Social Determinants of Health and its recommendations. Glob Health Promot. 2009;Suppl 1:23-7. Available from: doi:10.1177/1757975909103742.

36. Gabrysch S, Campbell OM. Still too far to walk: literature review of the determinants of delivery service use. BMC Pregnancy Childbirth. 2009;9(34). Available from: doi:10.1186/1471-2393-9-34.

37. Solar O, Irwin A. A conceptual framework for action on the social determinants of health. Geneva: World Health Organization, 2010.

38. Lawn JE, Blencowe H, Oza S, You D, Lee AC, Waiswa P, et al. Every Newborn: progress, priorities, and potential beyond survival. Lancet. 2014;384(9938):189-205. Available from: doi:10.1016/S0140-6736(14)60496-7.

39. Ministry of Health and Family Welfare. India Newborn Action Plan. New Delhi: 2014.

For peer review only - http://bmjopen.bmj.com/site/about/guidelines.xhtml

40. Sankar MJ, Neogi SB, Sharma J, Chauhan M, Srivastava R, Prabhakar PK, et al. State of newborn health in India. Journal of Perinatology. 2016;36(Suppl 3):S3-S8. Available from: doi:10.1038/jp.2016.183.

41. Zou G. A Modified Poisson Regression Approach to Prospective Studies with Binary Data. American Journal of Epidemiology. 2004;159(7):702-6. Available from: doi:10.1093/aje/kwh090.

42. Barros AJD, Victora CG. Measuring Coverage in MNCH: Determining and Interpreting Inequalities in Coverage of Maternal, Newborn, and Child Health Interventions. PLoS Med. 2013;10(5):e1001390. Available from: doi:10.1371/journal.pmed.1001390.

43. VanderWeele TJ. Mediation Analysis: A Practitioner's Guide. Annu Rev Public Health. 2016;37:17-32. Available from: doi:10.1146/annurev-publhealth-032315-021402.

44. Scott K, George AS, Ved RR. Taking stock of 10 years of published research on the ASHA programme: examining India's national community health worker programme from a health systems perspective. Health Research Policy and Systems. 2019;17(1):29. Available from: doi:10.1186/s12961-019-0427-0.

45. Sidney K, Tolhurst R, Jehan K, Diwan V, De Costa A. 'The money is important but all women anyway go to hospital for childbirth nowadays' - a qualitative exploration of why women participate in a conditional cash transfer program to promote institutional deliveries in Madhya Pradesh, India. BMC Pregnancy and Childbirth. 2016;16(1):47. Available from: doi:10.1186/s12884-016-0834-y.

46. Bruce SG, Blanchard AK, Gurav K, Roy A, Jayanna K, Mohan HL, et al. Preferences for infant delivery site among pregnant women and new mothers in Northern Karnataka, India. BMC Pregnancy Childbirth. 2015;15(49):1-10. Available from: doi:10.1186/s12884-015-0481-8.

47. Lim SS, Dandona L, Hoisington JA, James SL, Hogan MC, Gakidou E. India's Janani Suraksha Yojana, a conditional cash transfer programme to increase births in health facilities: an impact evaluation. Lancet. 2009;375(9730):2009-23. Available from: doi:10.1016/s0140-6736(10)60744-1.

48. Blanchard AK, Ansari S, Rajput R, Colbourn T, Houweling TAJ, Isac S, et al. Understanding the roles of community health workers in improving perinatal health equity in rural Uttar Pradesh, India: a qualitative study. Int J Equity Health. 2021;20(1):63. Available from: doi:10.1186/s12939-021-01406-5.

49. Sharma J, Leslie HH, Regan M, Nambiar D, Kruk ME. Can India's primary care facilities deliver? A cross-sectional assessment of the Indian public health system's capacity for basic delivery and newborn services. BMJ Open. 2018;8(6):e020532. Available from: doi:10.1136/bmjopen-2017-020532.

50. McPake B, Hanson K. Managing the public & private mix to achieve universal health coverage. The Lancet. 2016;388(10044):622-30. Available from: doi:10.1016/S0140-6736(16)00344-5.

51. Ministry of Health and Family Welfare. Ayushman Bharat: Comprehensive Primary Health Care through Health and Wellness Centers Operational Guidelines. New Delhi: Government of India, 2018.

For peer review only

52. Gogia S, Sachdev HS. Home visits by community health workers to prevent neonatal deaths in developing countries: A systematic review. Bulletin of the World Health Organization. 2010;88(9):658-66. Available from: doi:10.2471/BLT.09.069369.

53. Blanchard AK, Prost A, Houweling TAJ. Effects of community health worker interventions on socioeconomic inequities in maternal and newborn health in low-income and middle-income countries: a mixed-methods systematic review. Bmj Global Health. 2019;4(3). Available from: doi:10.1136/bmjgh-2018-001308.

54. Barros AJ, Ronsmans C, Axelson H, Loaiza E, Bertoldi AD, Franca GV, et al. Equity in maternal, newborn, and child health interventions in Countdown to 2015: a retrospective review of survey data from 54 countries. Lancet. 2012;379(9822):1225-33. Available from: doi:10.1016/S0140-6736(12)60113-5.

55. Black RE, Levin C, Walker N, Chou D, Liu L, Temmerman M. Reproductive, maternal, newborn, and child health: key messages from Disease Control Priorities 3rd Edition. The Lancet. 2016;388(10061):2811-24. Available from: doi:http://dx.doi.org/10.1016/S0140-6736(16)00738-8.

56. Angell BJ, Prinja S, Gupt A, Jha V, Jan S. The Ayushman Bharat Pradhan Mantri Jan Arogya Yojana and the path to universal health coverage in India: Overcoming the challenges of stewardship and governance. PLOS Medicine. 2019;16(3):e1002759. Available from: doi:10.1371/journal.pmed.1002759.

57. Upadhyay RP, Krishnan A, Rai SK, Chinnakali P, Odukoya O. Need to Focus Beyond the Medical Causes: a Systematic Review of the Social Factors Affecting Neonatal Deaths. Paediatr Perinat Epidemiol. 2014;28(2):127-37. Available from: doi:10.1111/ppe.12098.

58. Wall SN, Lee AC, Carlo W, Goldenberg R, Niermeyer S, Darmstadt GL, Keenan W, Bhutta ZA, Perlman J, Lawn JE. Reducing intrapartum-related neonatal deaths in low- and middle-income countries-what works? Semin Perinatol. 2010;34(6):395-407. Available from: doi:10.1053/j.semperi.2010.09.009.

For peer review only

For peer review only - http://bmjopen.bmj.com/site/about/guidelines.xhtml

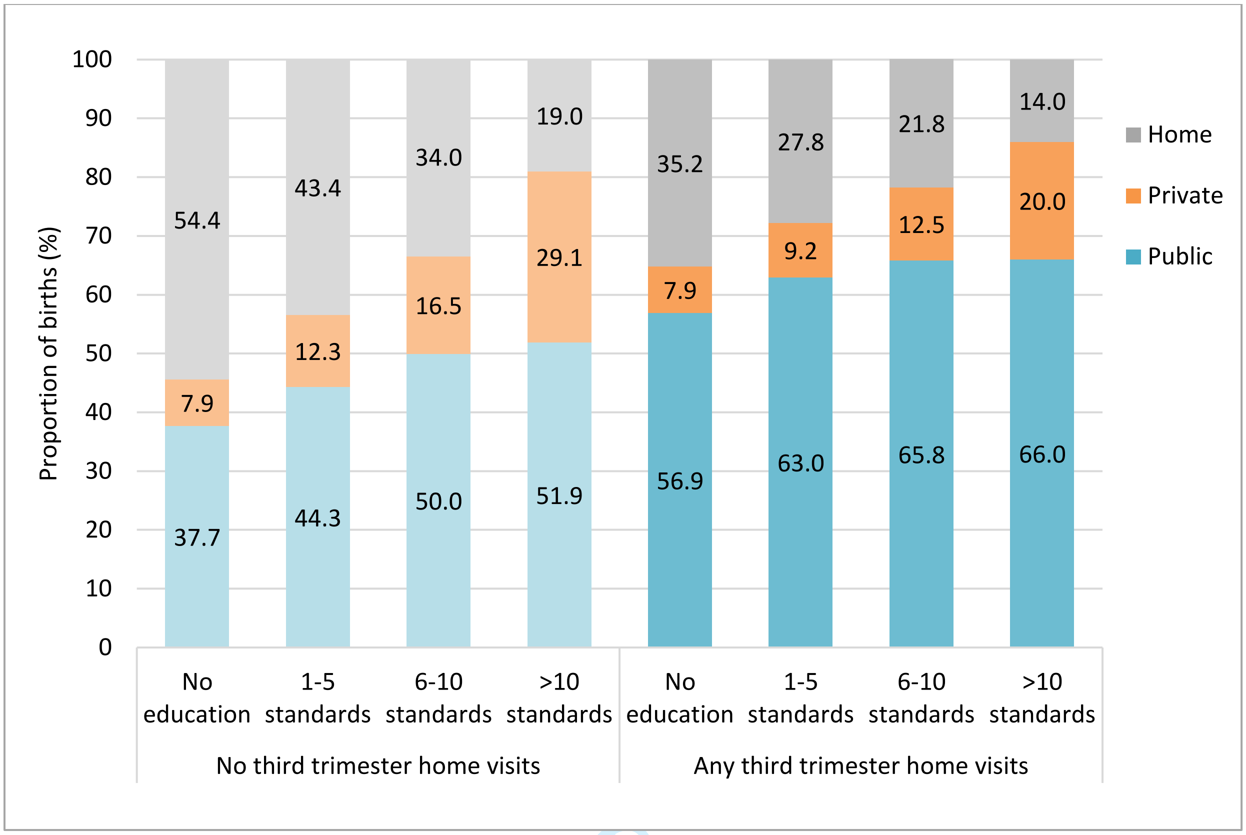

Figure 1: Proportion of women in each education group giving birth at home, public facilities, or private facilities, by exposure to any third trimester home visits

For peer review only - http://bmjopen.bmj.com/site/about/guidelines.xhtml

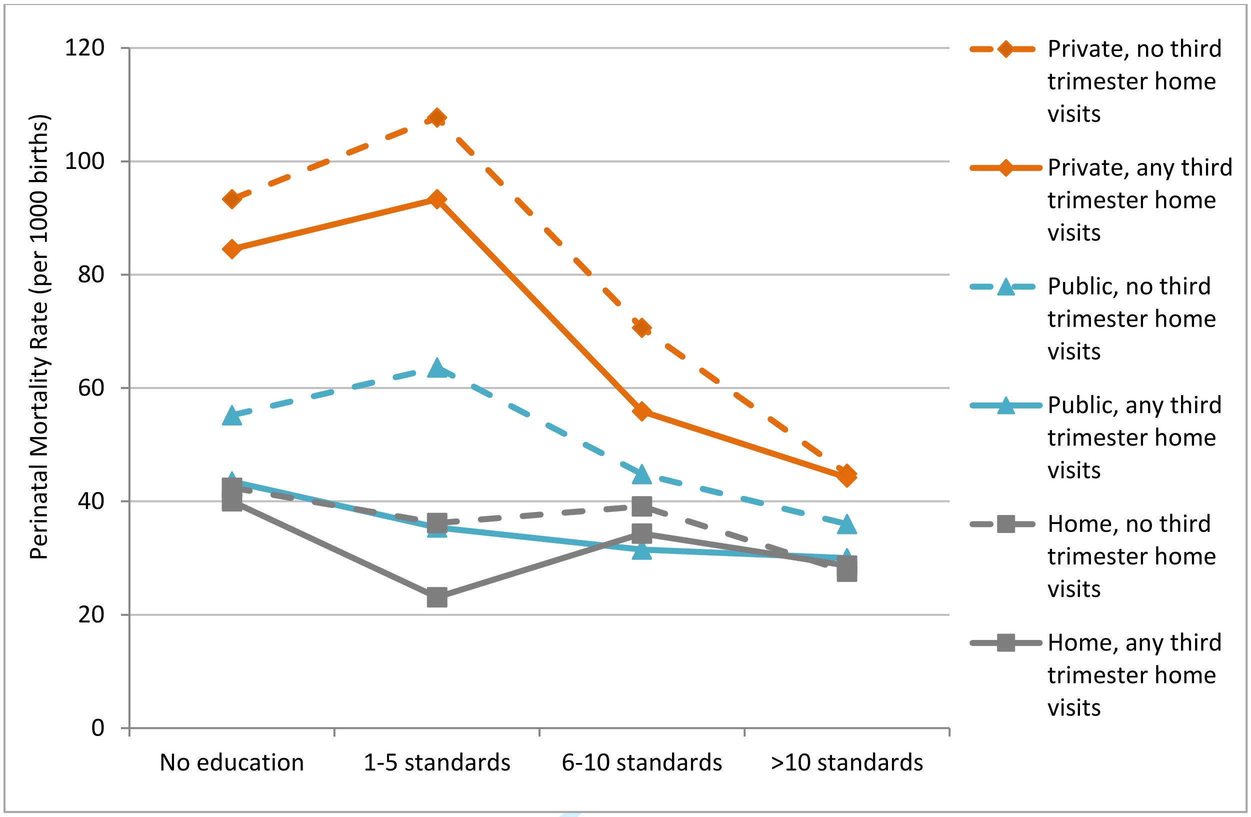

Figure 2: Perinatal mortality rate among women in each education group, by exposure to any third trimester home visits and place of birth

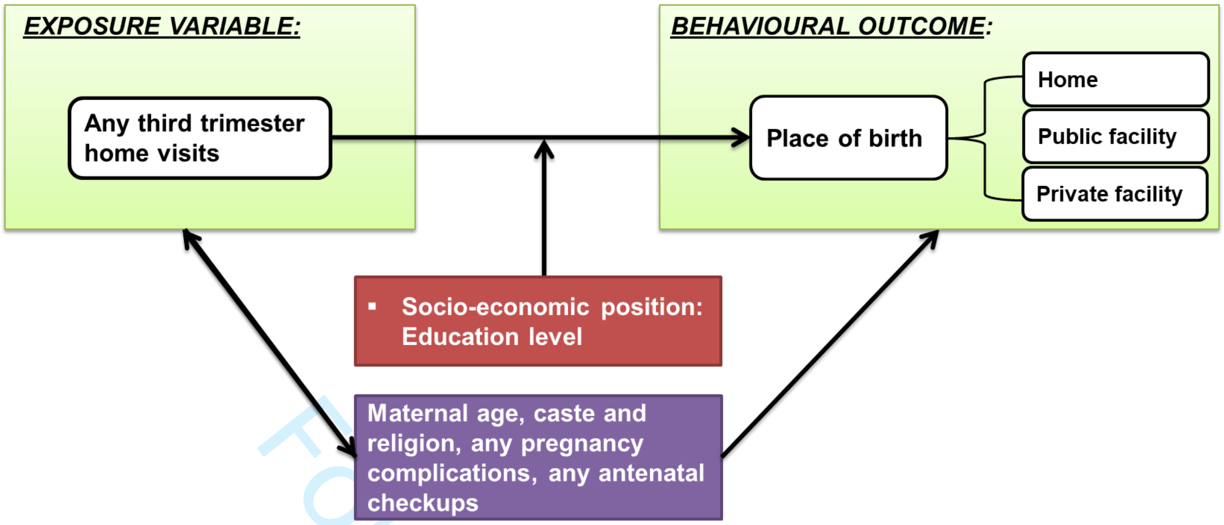

Supplementary figure 1: Conceptual model of the associations between women's exposure to third trimester home visits and place of birth

For peer review only

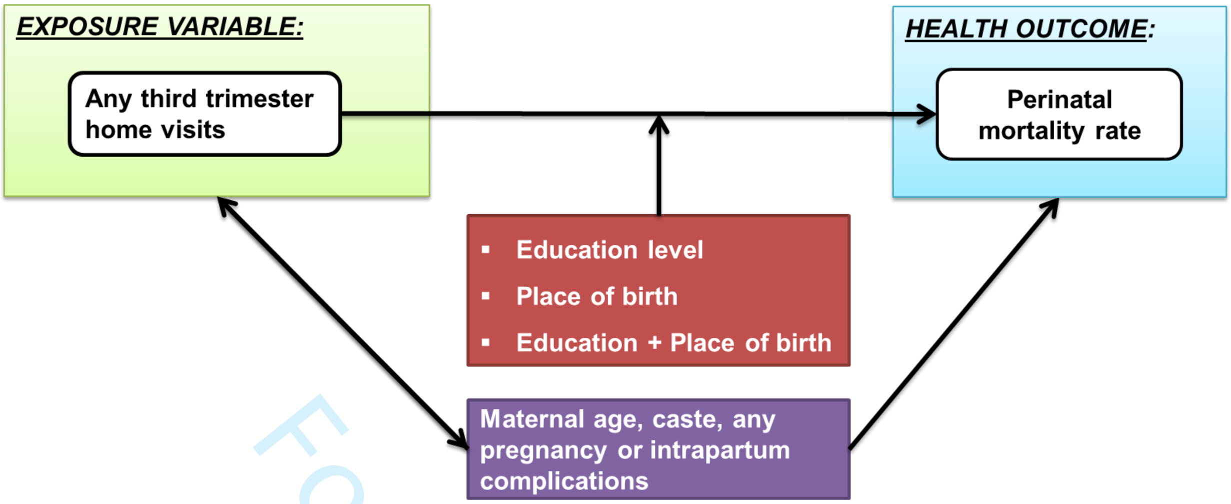

Supplementary figure 2: Conceptual model of the associations between women's exposure to third trimester home visits and perinatal mortality

For peer review only

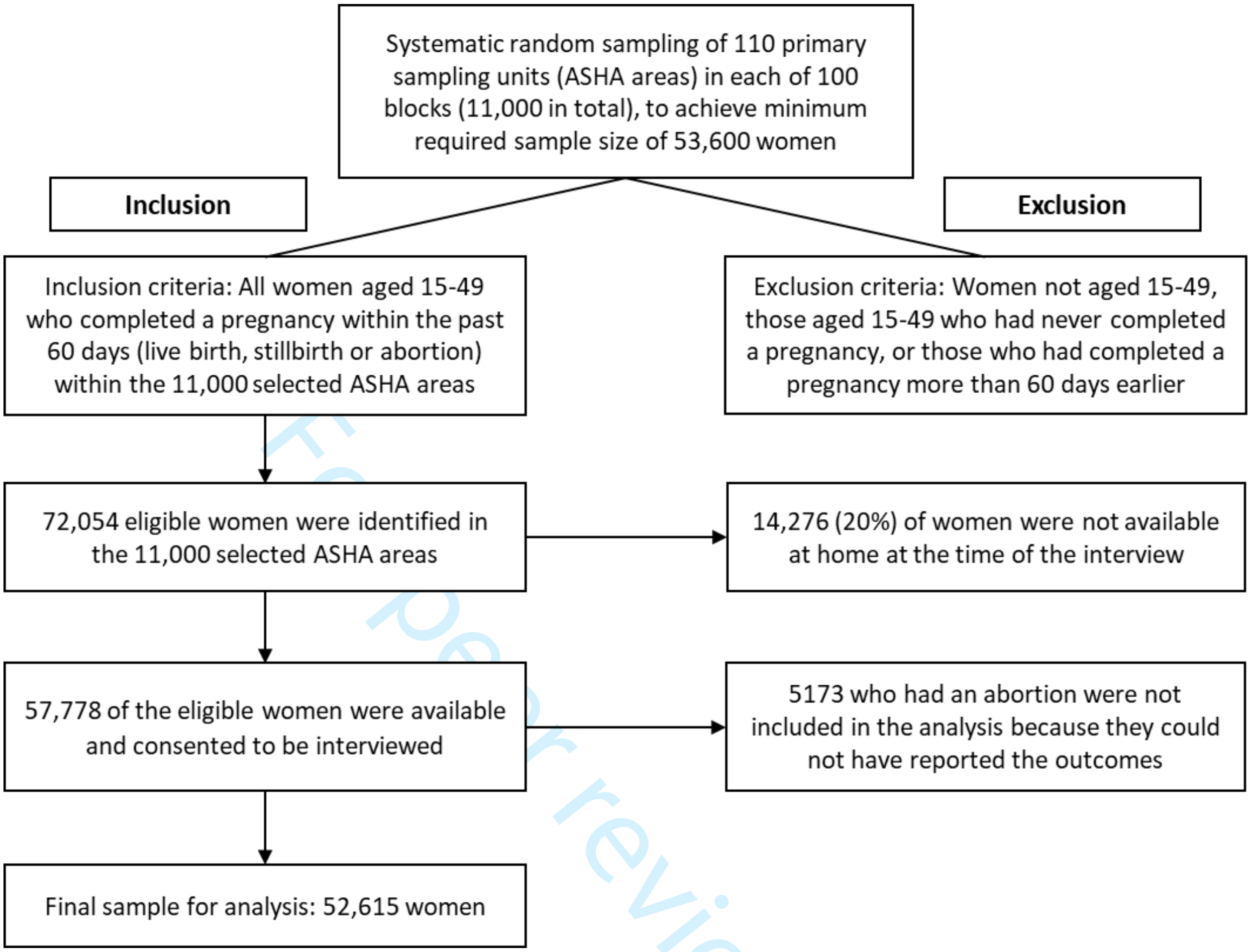

Supplementary figure 3: Flow diagram of the CBTS-1 sampling process

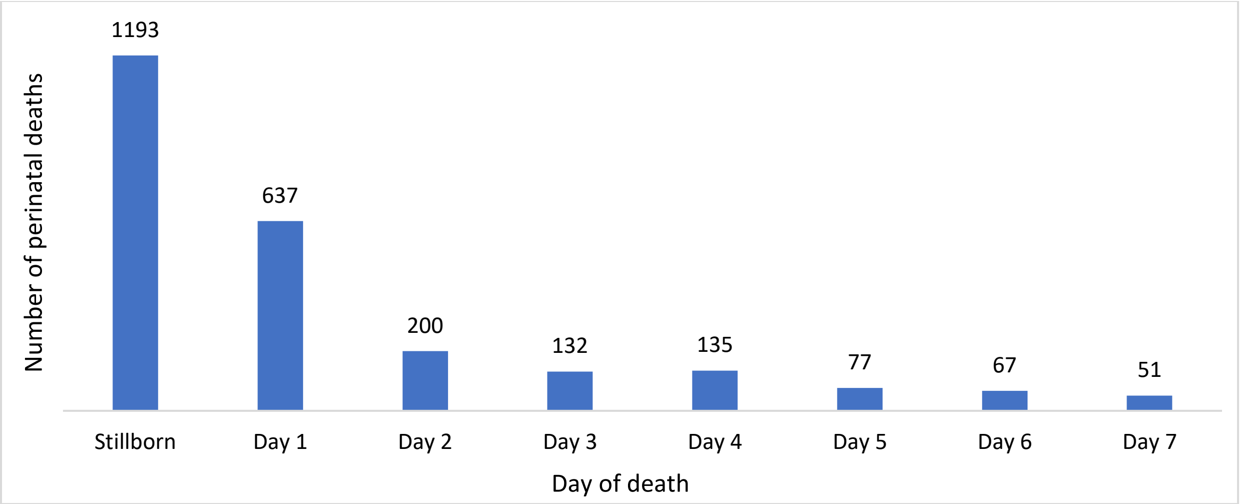

Supplementary figure 4: Distribution and timing of perinatal deaths among women who completed a pregnancy in the past two months

Supplementary table 1: Unadjusted associations between institutional delivery and any third trimester home visits, overall and by education level

Third trimester home visits	All education groups (n=52615)		None / illiterate (n= 30614)		1-5 standards (n=5693)		6-10 standards (n=9895)		>10 standards (n=6413)	
	Unadjusted % (95% CI)	Unadjusted RR (95% CI)	Unadjusted % (95% CI)	Unadjusted RR (95% CI)	Unadjusted % (95% CI)	Unadjusted RR (95% CI)	Unadjusted % (95% CI)	Unadjusted RR (95% CI)	Unadjusted % (95% CI)	Unadjusted RR (95% CI)
Institution (public and private) vs. home										
None (n=23914)	54.2 (53.6, 54.9)	Ref	45.6 (44.8, 46.4)	Ref	56.6 (54.6, 58.5)	Ref	66.0 (64.6, 67.5)	Ref	81.0 (79.5, 82.5)	Ref
Any (n=28701)	71.1 (70.6, 71.6)	1.31 (1.29, 1.33)	64.8 (64.0, 65.5)	1.42 (1.39, 1.45)	72.2 (70.6, 73.7)	1.28 (1.23, 1.33)	78.2 (77.1, 79.3)	1.18 (1.15, 1.22)	86.0 (84.9, 87.1)	1.06 (1.04, 1.09)
Public facility vs. home										
None (n=20997)	47.9 (47.2, 48.6)	Ref	41.0 (40.2, 41.8)	Ref	50.5 (48.3, 52.6)	Ref	59.3 (57.7, 61.0)	Ref	73.2 (71.2, 75.2)	Ref
Any (n=25682)	67.7 (67.1, 68.3)	1.41 (1.39, 1.44)	61.8 (61.0, 62.6)	1.51 (1.47, 1.54)	69.4 (67.7, 71.0)	1.37 (1.31, 1.44)	75.1 (73.9, 76.3)	1.27 (1.23, 1.31)	82.5 (81.1, 83.9)	1.13 (1.09, 1.16)
Private facility vs. home										
None (n=13860)	21.0 (20.4, 21.7)	Ref	12.7 (12.0, 13.4)	Ref	22.0 (19.8, 24.3)	Ref	32.6 (30.6, 34.7)	Ref	60.6 (57.9, 63.2)	Ref
Any (n=11318)	26.7 (25.9, 27.5)	1.27 (1.21, 1.32)	18.3 (17.4, 19.2)	1.44 (1.34, 1.55)	24.8 (22.3, 27.2)	1.12 (0.98, 1.29)	36.4 (34.3, 38.5)	1.11 (1.02, 1.21)	58.8 (56.1, 61.6)	0.97 (0.91, 1.04)

For peer review only

Supplementary table 2: Unadjusted associations between perinatal mortality rate and any third trimester home visits, overall and by education and place of birth

Third trimester home visits	All education groups (n=52588)		None / illiterate (n= 30600)		1-5 standards (n=5689)		6-10 standards (n=9887)		>10 standards (n=6412)	
	Unadjusted PMR (95% CI)	Unadjusted RR (95% CI)	Unadjusted PMR (95% CI)	Unadjusted RR (95% CI)	Unadjusted PMR (95% CI)	Unadjusted RR (95% CI)	Unadjusted PMR (95% CI)	Unadjusted RR (95% CI)	Unadjusted PMR (95% CI)	Unadjusted RR (95% CI)
All births										
Any (n=28686)	40.8 (38.5, 43.1)	Ref	45.4 (42.2, 48.7)	Ref	37.3 (30.8, 43.8)	Ref	35.1 (30.4, 39.9)	Ref	32.7 (27.0, 38.4)	Ref
None (n=23902)	49.5 (46.8, 52.3)	1.21 (1.12, 1.31)	51.3 (47.7, 54.8)	1.13 (1.02, 1.26)	57.1 (47.9, 66.4)	1.53 (1.21, 1.94)	47.1 (40.7, 53.6)	1.34 (1.11, 1.63)	37.0 (29.8, 44.1)	1.13 (87.3, 1.47)
Home delivery										
Any (n=8295)	36.5 (32.5, 40.6)	Ref	39.9 (34.8, 45.1)	Ref	23.1 (13.3, 32.8)	Ref	34.3 (24.2, 44.4)	Ref	28.9 (14.4, 43.0)	Ref
None (n=10935)	40.7 (37.0, 44.4)	1.11 (0.96, 1.29)	42.4 (37.9, 46.8)	1.06 (0.90, 1.25)	36.2 (24.9, 47.5)	1.57 (0.93, 2.65)	39.1 (29.0, 49.3)	1.14 (0.77, 1.69)	27.6 (13.3, 41.8)	0.96 (0.47, 1.97)
Public facility delivery										
Any (n=17426)	38.0 (35.1, 40.8)	Ref	43.5 (39.3, 47.7)	Ref	35.4 (27.4, 43.4)	Ref	31.5 (25.9, 37.1)	Ref	30.0 (23.4, 36.8)	Ref
None (n=10052)	51.3 (47.0, 55.6)	1.35 (1.21, 1.51)	55.2 (49.2, 61.3)	1.27 (1.10, 1.47)	63.6 (48.9, 78.2)	1.80 (1.30, 2.48)	44.8 (35.9, 53.8)	1.42 (1.09, 1.86)	36.0 (26.2, 45.8)	1.20 (0.84, 1.71)
Private facility delivery										
Any (n=3017)	68.6 (59.6, 77.6)	Ref	84.5 (69.1, 99.9)	Ref	93.3 (60.4, 126.3)	Ref	55.9 (39.0, 72.7)	Ref	44.2 (29.4, 58.9)	Ref
None (n=2915)	76.5 (66.9, 86.1)	1.11 (0.92, 1.34)	93.3 (76.5, 110.0)	1.10 (0.85, 1.42)	107.7 (72.5, 143.0)	1.15 (0.71, 1.87)	70.5 (51.3, 89.8)	1.26 (0.84, 1.90)	44.9 (30.3, 59.4)	1.02 (0.64, 1.62)

For peer review only

STROBE 2007 (v4) Statement—Checklist of items that should be included in reports of *cross-sectional studies*

Section/Topic	Item #	Recommendation	Reported on page #
**Title and abstract**	1	(*a*) Indicate the study's design with a commonly used term in the title or the abstract	1-2
		(*b*) Provide in the abstract an informative and balanced summary of what was done and what was found	2
**Introduction**			
Background/rationale	2	Explain the scientific background and rationale for the investigation being reported	4-5
Objectives	3	State specific objectives, including any prespecified hypotheses	5
**Methods**			
Study design	4	Present key elements of study design early in the paper	5-6
Setting	5	Describe the setting, locations, and relevant dates, including periods of recruitment, exposure, follow-up, and data collection	5-7
Participants	6	(*a*) Give the eligibility criteria, and the sources and methods of selection of participants	6
Variables	7	Clearly define all outcomes, exposures, predictors, potential confounders, and effect modifiers. Give diagnostic criteria, if applicable	7-9
Data sources/ measurement	8*	For each variable of interest, give sources of data and details of methods of assessment (measurement). Describe comparability of assessment methods if there is more than one group	7-9
Bias	9	Describe any efforts to address potential sources of bias	7, 17
Study size	10	Explain how the study size was arrived at	6-7
Quantitative variables	11	Explain how quantitative variables were handled in the analyses. If applicable, describe which groupings were chosen and why	7-10
Statistical methods	12	(*a*) Describe all statistical methods, including those used to control for confounding	9-10
		(*b*) Describe any methods used to examine subgroups and interactions	9-10
		(*c*) Explain how missing data were addressed	7
		(*d*) If applicable, describe analytical methods taking account of sampling strategy	6
		(*e*) Describe any sensitivity analyses	10
**Results**			

For peer review only

Participants	13*	(a) Report numbers of individuals at each stage of study—eg numbers potentially eligible, examined for eligibility, confirmed eligible, included in the study, completing follow-up, and analysed	6-7
		(b) Give reasons for non-participation at each stage	6
		(c) Consider use of a flow diagram	Supplementary figure 3
Descriptive data	14*	(a) Give characteristics of study participants (eg demographic, clinical, social) and information on exposures and potential confounders	11-12
		(b) Indicate number of participants with missing data for each variable of interest	12 (Table 1 footnote a)
Outcome data	15*	Report numbers of outcome events or summary measures	11-12, 15
Main results	16	(*a*) Give unadjusted estimates and, if applicable, confounder-adjusted estimates and their precision (eg, 95% confidence interval). Make clear which confounders were adjusted for and why they were included	12-16, supplementary tables 1-2
		(*b*) Report category boundaries when continuous variables were categorized	NA
		(*c*) If relevant, consider translating estimates of relative risk into absolute risk for a meaningful time period	12-16
Other analyses	17	Report other analyses done—eg analyses of subgroups and interactions, and sensitivity analyses	12-16, Figures 1-2
**Discussion**			
Key results	18	Summarise key results with reference to study objectives	17
Limitations	19	Discuss limitations of the study, taking into account sources of potential bias or imprecision. Discuss both direction and magnitude of any potential bias	17
Interpretation	20	Give a cautious overall interpretation of results considering objectives, limitations, multiplicity of analyses, results from similar studies, and other relevant evidence	17-20
Generalisability	21	Discuss the generalisability (external validity) of the study results	17
**Other information**			
Funding	22	Give the source of funding and the role of the funders for the present study and, if applicable, for the original study on which the present article is based	20

*Give information separately for cases and controls in case-control studies and, if applicable, for exposed and unexposed groups in cohort and cross-sectional studies.

For peer review only

Note: An Explanation and Elaboration article discusses each checklist item and gives methodological background and published examples of transparent reporting. The STROBE checklist is best used in conjunction with this article (freely available on the Web sites of PLoS Medicine at http://www.plosmedicine.org/, Annals of Internal Medicine at http://www.annals.org/, and Epidemiology at http://www.epidem.com/). Information on the STROBE Initiative is available at www.strobe-statement.org.

For peer review only
